# Fluorescence‐Lifetime Imaging and Super‐Resolution Microscopies Shed Light on the Directed‐ and Self‐Assembly of Functional Porphyrins onto Carbon Nanotubes and Flat Surfaces

**DOI:** 10.1002/chem.201605232

**Published:** 2017-05-19

**Authors:** Boyang Mao, David G. Calatayud, Vincenzo Mirabello, Navaratnarajah Kuganathan, Haobo Ge, Robert M. J. Jacobs, Ashley M. Shepherd, José A. Ribeiro Martins, Jorge Bernardino De La Serna, Benjamin J. Hodges, Stanley W. Botchway, Sofia I. Pascu

**Affiliations:** ^1^ Department of Chemistry University of Bath Claverton Down BA2 7AY, Bath UK; ^2^ National Graphene Institute and School of Physics and Astronomy The University of Manchester Booth Street East Manchester M13 9PL UK; ^3^ Department of Electroceramics Instituto de Ceramica y Vidrio - CSIC Madrid 28049 Spain; ^4^ Department of Materials Imperial College London, South Kensington London SW7 2AZ UK; ^5^ Department of Chemistry Chemistry Research Laboratory University of Oxford Mansfield Road Oxford OX1 3TA UK; ^6^ Centro de Engenharia Biológica and Departamento de Química Universidade do Minho Campus de Gualtar 4710-057 Braga Portugal; ^7^ Central Laser Facility Rutherford Appleton Laboratory Research Complex at Harwell STFC Didcot OX11 0QX UK

**Keywords:** carbon nanotubes, nanostructures, optically active materials, self-assembly, super-resolution STED imaging

## Abstract

Functional porphyrins have attracted intense attention due to their remarkably high extinction coefficients in the visible region and potential for optical and energy‐related applications. Two new routes to functionalised SWNTs have been established using a bulky Zn^II^‐porphyrin featuring thiolate groups at the periphery. We probed the optical properties of this zinc(II)‐substituted, bulky aryl porphyrin and those of the corresponding new nano‐composites with single walled carbon nanotube (SWNTs) and coronene, as a model for graphene. We report hereby on: i) the supramolecular interactions between the pristine SWNTs and Zn^II^‐porphyrin by virtue of π–π stacking, and ii) a novel covalent binding strategy based on the Bingel reaction. The functional porphyrins used acted as dispersing agent for the SWNTs and the resulting nanohybrids showed improved dispersibility in common organic solvents. The synthesized hybrid materials were probed by various characterisation techniques, leading to the prediction that supramolecular polymerisation and host–guest functionalities control the fluorescence emission intensity and fluorescence lifetime properties. For the first time, XPS studies highlighted the differences in covalent versus non‐covalent attachments of functional metalloporphyrins to SWNTs. Gas‐phase DFT calculations indicated that the Zn^II^‐porphyrin interacts non‐covalently with SWNTs to form a donor–acceptor complex. The covalent attachment of the porphyrin chromophore to the surface of SWNTs affects the absorption and emission properties of the hybrid system to a greater extent than in the case of the supramolecular functionalisation of the SWNTs. This represents a synthetic challenge as well as an opportunity in the design of functional nanohybrids for future sensing and optoelectronic applications.

## Introduction

Since the discovery of single‐walled carbon nanotubes (SWNTs)^1^ there has been a sustained interest in this material from both academic and industrial research perspectives. This is due to the fact that its rigid rod‐like tubular structure, coupled with a highly delocalised extended π‐electron system, confers its unique mechanical and electronic properties, thus offering promises of technological advances.^2^ Ongoing studies show that, upon functionalisation of SWNTs with small molecules, it is possible to develop prototype optoelectronic and photovoltaic devices.^3^ Thus, the interactions between fluorescent molecules, as well as semiconductive polymers, and the aromatic surface of SWNTs have attracted significant interest due to the potential application of these new nanohybrids, particularly in solar cells.^4^

One of most attractive properties of SWNTs is the possibility to chemically modify their surfaces by two general approaches; covalent or non‐covalent synthetic methodologies. The intrinsic sp^2^ nature of the C−C bond of the SWNT allows reaction with organic molecules bearing specific functional groups that react with the outer walls of the tubular material. Porphyrins have been used as versatile chromophores, having the ability to act as dispersing agents for carbon nanomaterials and to decorate C_60_,^5^ carbon nanotubes,^6^ graphene oxide^7^ or nanohorns^8^ in artificial photosynthetic devices.^9^ SWNTs can interact via aromatic stacking with electron‐rich planar molecules resulting in supramolecular donor–acceptor complexes. Such molecules, including porphyrins (planar, electron‐rich, aromatic species), are characterised by remarkably high extinction coefficients in the visible region. Tailor‐made porphyrins designed to include thiol units suitable for dynamic covalent chemistry have been designed and their ability to recognise fullerenes or small molecular acceptors such as naphtyldiimides in a controlled way has been demonstrated.^5^, ^10^ Such nanohybrids have the potential to offer valuable new technologies in the field of nano‐optoelectronic devices for energy conversion, sensing and biological applications.4c, 11 The recent work by Strano and collaborators has demonstrated the potential of non‐covalent sensors based on SWNTs in biology.^13^ Thiol‐derivatised functional Zn^II^ porphyrin‐based systems^7^ have not been investigated thus far in the context of generating new SWNTs adducts with targeted optical properties. The combination of the unique electron transport properties of the SWNTs together with the promising photochemical properties of tailor‐made functional porphyrins could render their new adducts as potential candidates for dispersible solid state scaffolds in nano‐optoelectronic devices and bio‐sensing. Crucially, using X‐ray photoelectron spectroscopy (XPS), as well as fluorescence imaging techniques, we also demonstrated here that the formation of the porphyrin–SWNTs supramolecular networks can be controlled through directed‐ and self‐assembly, giving rise to extended nanostructures with interesting optical properties.

We characterised these materials in a thin film environment using fluorescence lifetime measurements via time‐correlated single photon counting (TCSPC) combined with multi‐photon laser scanning confocal microscopy; this allows us to gain detailed information on the environment of the fluorophore upon immobilisation within the nanomaterial hybrid and estimate the influence of the different linking strategies on the emerging materials′ optical properties by observing their quenching characteristics in solution and in the solid state.^7^, ^12^

Characterisation approaches rely on several different efficient and novel physical measurement methods coupled with multi‐scale microscopies to identify unequivocally the existence of a covalent and non‐covalent linkage between small organic molecules and carbon nanomaterials. The approach reported herein can become a general methodology for achieving control of photochemistry and sensing capabilities of a wide range of optical and energy materials by tailoring the nature of the interactions between components using directed‐ and self‐assembly processes in solution and thin film.

## Results and Discussion

### Synthesis and structural investigations: Single‐crystal X‐ray diffraction and super‐resolution imaging

We report on the synthesis of novel SWNT–porphyrin nanohybrids. The porphyrin of choice is a member of a family of substituted aryl porphyrins known to be soluble in most common solvents, for example, toluene, dichloromethane and ethanol, and easily derivatised (compound **1**). New functional materials were synthesised by directed‐ and self‐assembly methods; the synthetic strategies involve, respectively, a new dynamic covalent route (based on the thiol‐disulfide exchange involving a Zn^II^‐porphyrin dithiolated synthon and a thiolated SWNTs surface) and a simple and straight forward non‐covalent linking strategy.

**Figure 1:**
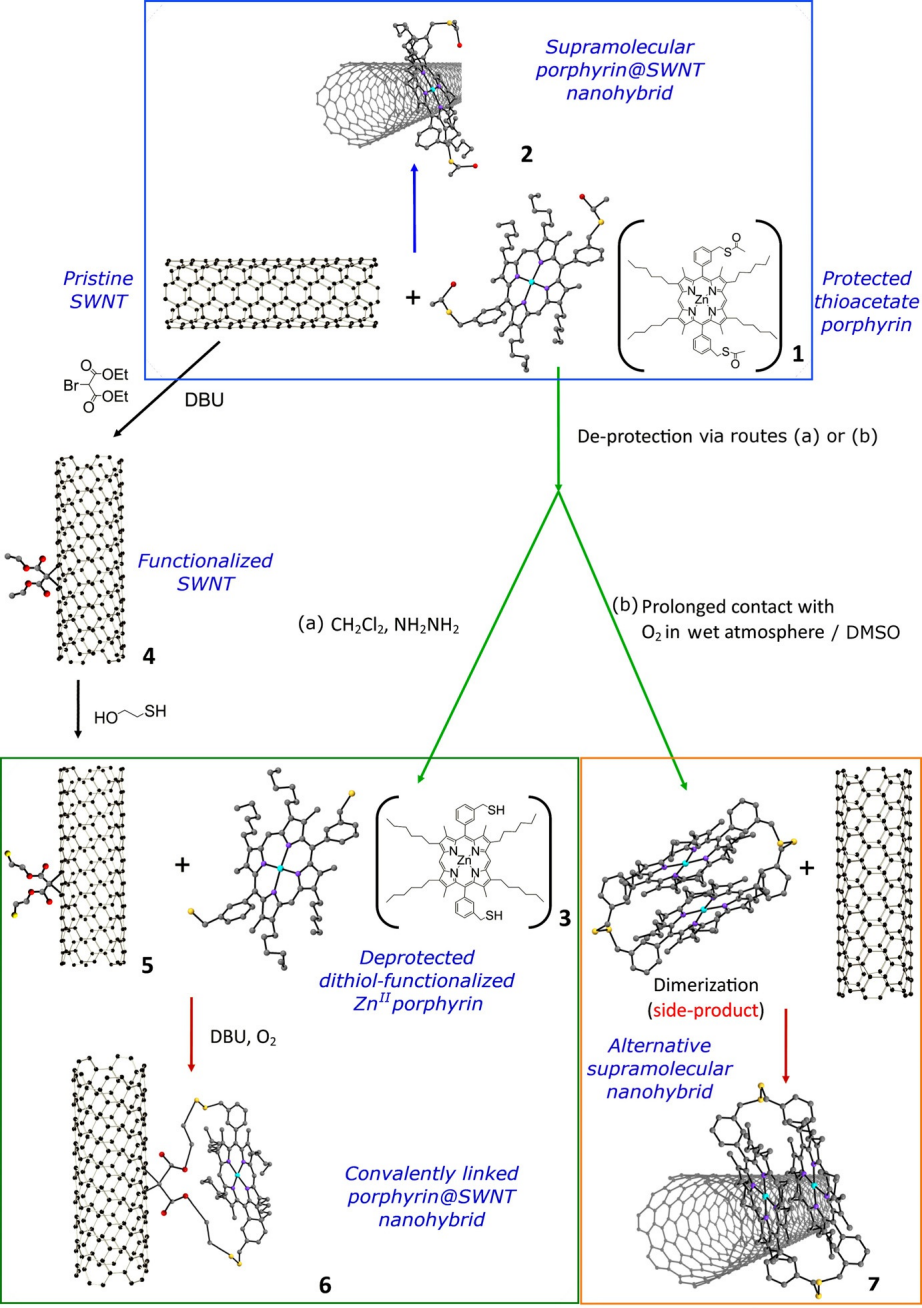
Schematic diagram of the formation of covalently and non‐covalently linked Zn^II^‐porphyrin@SWNTs hybrids **2** and **6**. Element colour: oxygen–red, sulfur–yellow, nitrogen–purple, light blue–zinc, grey–carbon. The structure of the disulfide‐linked side‐product was obtained by single‐crystal X‐ray diffraction; a ball and stick representation is used for clarity.

The non‐covalent, outer surface modification of SWNTs was carried out via supramolecular π–π interaction of the aryl thioacetate‐functionalised hexyl‐substituted Zn^II^‐porphyrin (**1**) (Figure 1) in a one‐step process, leading to the formation of complex **2**.

For the generation of the covalently linked system, the surface of the SWNTs was first modified to incorporate a thiol group capable of reacting with the ‐SH groups of **3** and generating S−S disulfide bridges. The Bingel reaction was used to generate thiol groups on the surface of the nanotubes, which in turn react with the SH‐deprotected Zn^II^‐porphyrin (**3**), generating a covalent bond^14^ at the SWNTs surface (Figure 1) via thiol‐disulfide exchange.

The Zn^II^‐porphyrin (**1**) has been specifically chosen to incorporate four hexyl chains on the exocyclic positions of the macrocycle ring to enhance its solubility in common organic solvents. Moreover, two aryl thioacetate side groups have been included with the intention to help direct the dynamic covalent linkage of the porphyrin to the thiol‐functionalised outer surface of the SWNTs. For the synthesis of the free porphyrin and zinc(II) porphyrin on a milligram scale, suitable for further synthetic manipulations, the general reaction methodology proposed by Twyman and Sanders was adapted.^15^ This method involved 1‐iodohexane, pentane‐2,4‐dione and benzyl 3‐oxobutanonate as starting materials, and is described elsewhere.^16^ The hexyl‐derived pyrrole acts as the basic building unit from which the functional porphyrins are assembled.^17^ This synthetic method allowed the incorporation of a versatile range of peripheral groups at the porphyrin core, including extended alkyl chains or bulky substituents. The nature of the metal centre can be further modified to tune their ability in host–guest recognition of relevance for photovoltaic applications.

**Figure 2:**
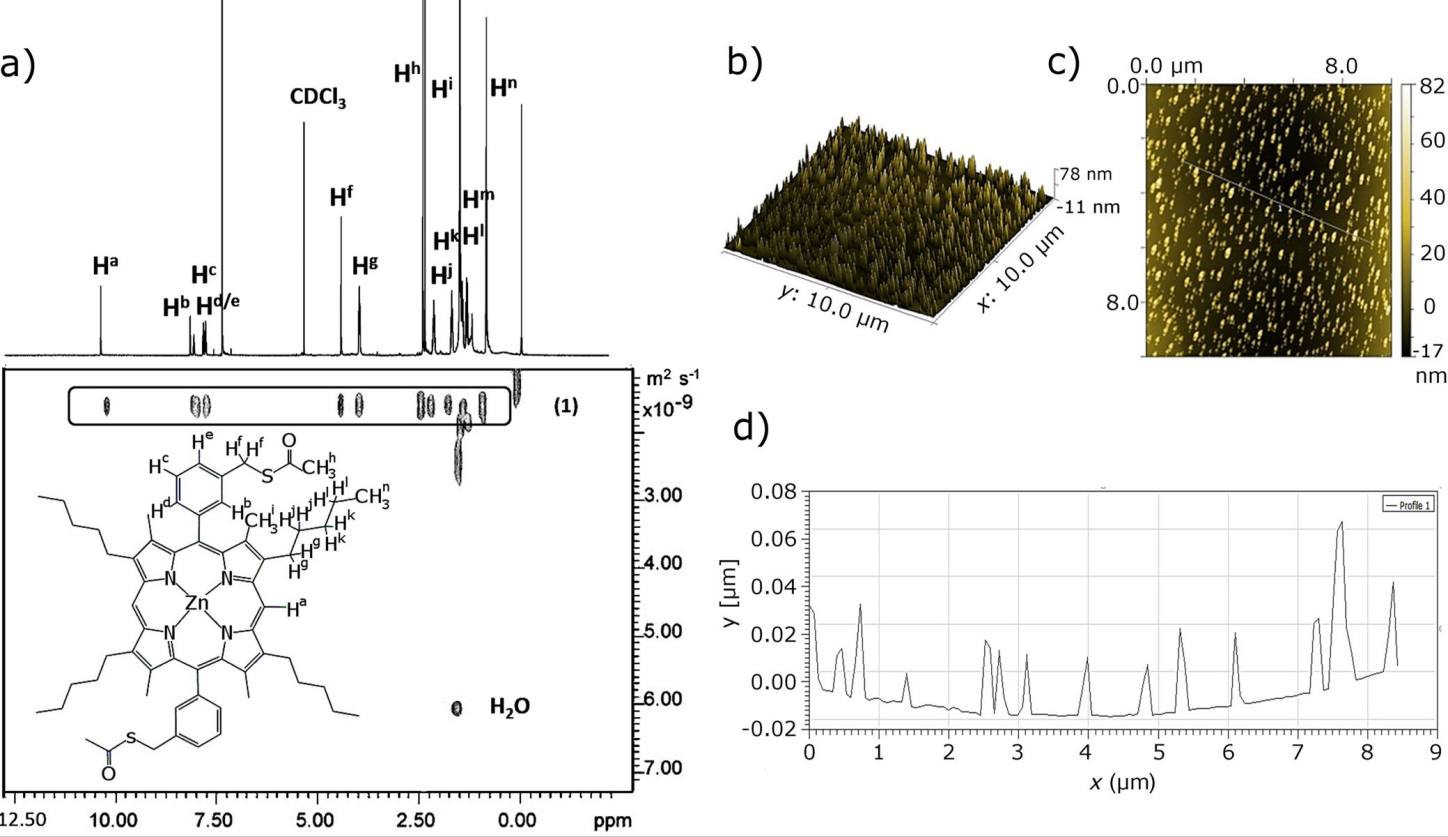
a) ^1^H DOSY NMR spectrum (500 MHz, 298 K, CDCl_3_) of compound **1**. The diffusion coefficient of 1.62 10^−9^ m^2^ s^−1^ limited the ability of this Zn^II^‐porphyrin to aggregate in solution even at roughly 5 mm (needed to record a spectrum). b) 3D tapping‐mode (TM) AFM image of Zn^II^‐porphyrin (**1**) self‐assembly on mica. For the TM AFM measurement, the Zn^II^‐porphyrin sample was prepared by spin coating at 3000 rpm of the Zn^II^‐porphyrin solution (18.3 μm in CHCl_3_) onto a 2 cm^2^ mica substrate. c) TM AFM image of a Zn^II^‐porphyrin self‐assembled crystal. d) Profile analysis showing the heights of line.

The resulting porphyrins were fully characterised in solution and in the solid state, and X‐ray diffraction studies showed that indeed, under conditions specific for dynamic covalent chemistry, the thiol groups lead to the formation of disulfide‐bridged dimers. This new compound could be isolated and fully characterised (see the Supporting Information for details). Diffusion‐ordered spectroscopy (DOSY) was employed to explore the possibility that porphyrins of type **1** self‐aggregate in the solutions used to perform experiments on the NMR scale. This method was deemed a convenient spectroscopic tool as it has been already applied in supramolecular chemistry to identify inter‐ and intramolecular self‐assembled aggregates and aided the understanding of host–guest interactions in solution.^18^ Here, we estimated the experimental diffusion coefficients (*D*_exp_) to predict the tendency of porphyrin molecules of type (**1**) and of its free‐base precursor to self‐aggregate.^19^ DOSY spectra of Zn^II^‐porphyrin (**1**) and of its free‐based porphyrin precursor were acquired at 298 K and are shown in Figure 2 and in the Supporting Information (Figure S**7**). The *D*_exp_ for Zn^II^‐porphyrin (**1**) and metal‐free porphyrin precursor were estimated to be 1.62×10^−9^ m^2^ s^−1^ and 4.57×10^−10^ m^2^ s^−1^, respectively. These findings indicate the low tendency of these porphyrins to self‐aggregate in solution at the concentration needed to obtain an NMR spectrum (5 mm). This is consistent with previous reports for simpler porphyrin systems such as tetraphenylporphyrin (TPP).^19^ It has already been shown that Zn‐porphyrins can be functionalised and their self‐assembly can generate nanorings of 5–60 porphyrin units.17a, 20 Earlier observations by AFM or STM measurements performed on drop‐casted solutions show that such supramolecular architectures can be described as nanocrystalline aggregates of porphyrins, having a shape of a ring or tower, with sizes varying in the range of hundreds of nanometers, depending on the imaging method used to evaluate the size.^7^, 11c, 21

Confocal fluorescence imaging of such films formed on borosilicate glass (when a solution of the chromophore was allowed to dry slowly on a glass surface, see below and the Supporting Information) suggests that a self‐assembly process is likely to occur, but the precise shape or size could not be asserted due to the low resolution. We were intrigued by the objects formed on insulating surfaces (e.g., mica or borosilicate glass) for the Zn^II^‐porphyrins and by their optical emissive properties.

**Figure 3:**
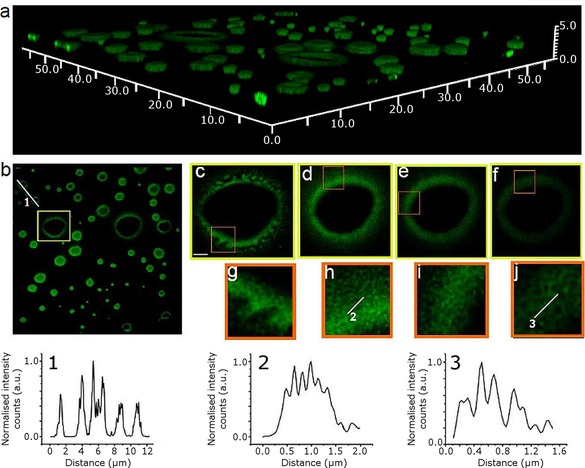
Stimulated emission depletion (STED) microscopy of a Zn^II^‐porphyrin (**1**) thin film (CHCl_3_ solution drying on a borosilicate glass surface); *λ*_ex_=488 nm, STED=775 nm, laser power intensity=179 mW, *λ*_em_=580 nm. Top image a) Deconvolved image of a 3D reconstruction obtained from stacked individual STED images. The image is shown in perspective for better appreciation of the different heights of the tubular structures. X, Y and Z scale bars: 50×50×5 μm). Below, from right to left, deconvolved image of the 3D reconstruction as projected in the XY plane (b). The line profile crossing 3 different tubes is shown in the graph below (1). The highlighted squared region in image (b) is magnified to the right; each image from left to right (c–f), is showing the same tubular structure at different *z*‐planes, with a difference between planes of 300 nm, c) being the lowermost region in contact with the surface, and f) the uppermost. Zoom‐in images of the squared regions can be found below each image (g–j). The lines profiles drawn in (h) and (j) are represented in the graphs (2) and (3), respectively.

Super‐resolution imaging, additionally to single‐ and multi‐photon confocal fluorescence imaging techniques, was employed to further characterise the sub‐structure morphologies of the tower‐like aggregates observed for **1** on surfaces, and thus aided our understanding thereof. These optical imaging experiments on thin films were crucially necessary, since with solution experiments by DOSY at a concentration of roughly 5 mm (needed to record NMR spectra in CDCl_3_) little convincing evidence for aggregation was found. Stimulated emission depletion (STED) microscopy with a 775 nm depletion laser was used to assess the morphology and the emission properties of the solid aggregates of the Zn^II^‐porphyrin (**1**) previously observed by confocal fluorescence as well as AFM (Figure 3). Interestingly, STED deconvolution and surface 3D‐rendered imaging reveals that such Zn^II^‐porphyrin (**1**) microcrystalline cylinders are roughly 2 μm in height. The larger and smaller rings of the tubular structures have a thickness of 611.3±3.7 and 323.5±2.7 nm, respectively. The tubular sub‐structure is formed of smaller concatenated rings, which grow in size from the bottom to the top. The smallest ones observed were of 50 nm diameter and the largest of 130 nm, found both at the top and towards the periphery of the basal plane (however, this method does not allow the exploration of aggregates smaller than ca 30 nm). Interestingly, the free‐base porphyrin precursor only displayed a very weak STED effect, and the size of the porphyrin stacks could not be fully interpreted by this method, whereas the SWNTs‐functionalised porphyrins (synthesised as described below) did not yield such an effect at all, and therefore could not be imaged by super resolution under these conditions. The presence of a Zn^II^ centre, together with the thiol‐protected aryl groups and the ability to form aromatically stacked aggregates, play a crucial role in mediating the nature of the self‐assembly of these porphyrins on insulating surfaces. The application of super resolution, STED, has allowed the observation of these structures on nanoscale (<100 nm). As yet, the effect of such “tower”‐like structure formations on the optical properties of the nanomaterial emerging remained unexplored.

As stated above, the pristine strands of SWNTs were subsequently used as templates in the attempt to control the formation of such cylindrical supramolecular assemblies of porphyrins, offering an alternative synthetic route to the covalent functionalisation of SWNTs, which has already attracted broad attention in the scientific community.9a, 22

Figure 1 shows the reaction scheme of the Zn^II^‐porphyrin@SWNTs non‐covalent complex (**2**). A dispersion of SWNTs in chloroform and a Zn^II^‐porphyrin solution in the same solvent were mixed and stirred for 24 h at room temperature and the solid nanocomposites obtained were collected by nanofiltration and washing with ethanol.

**Figure 4:**
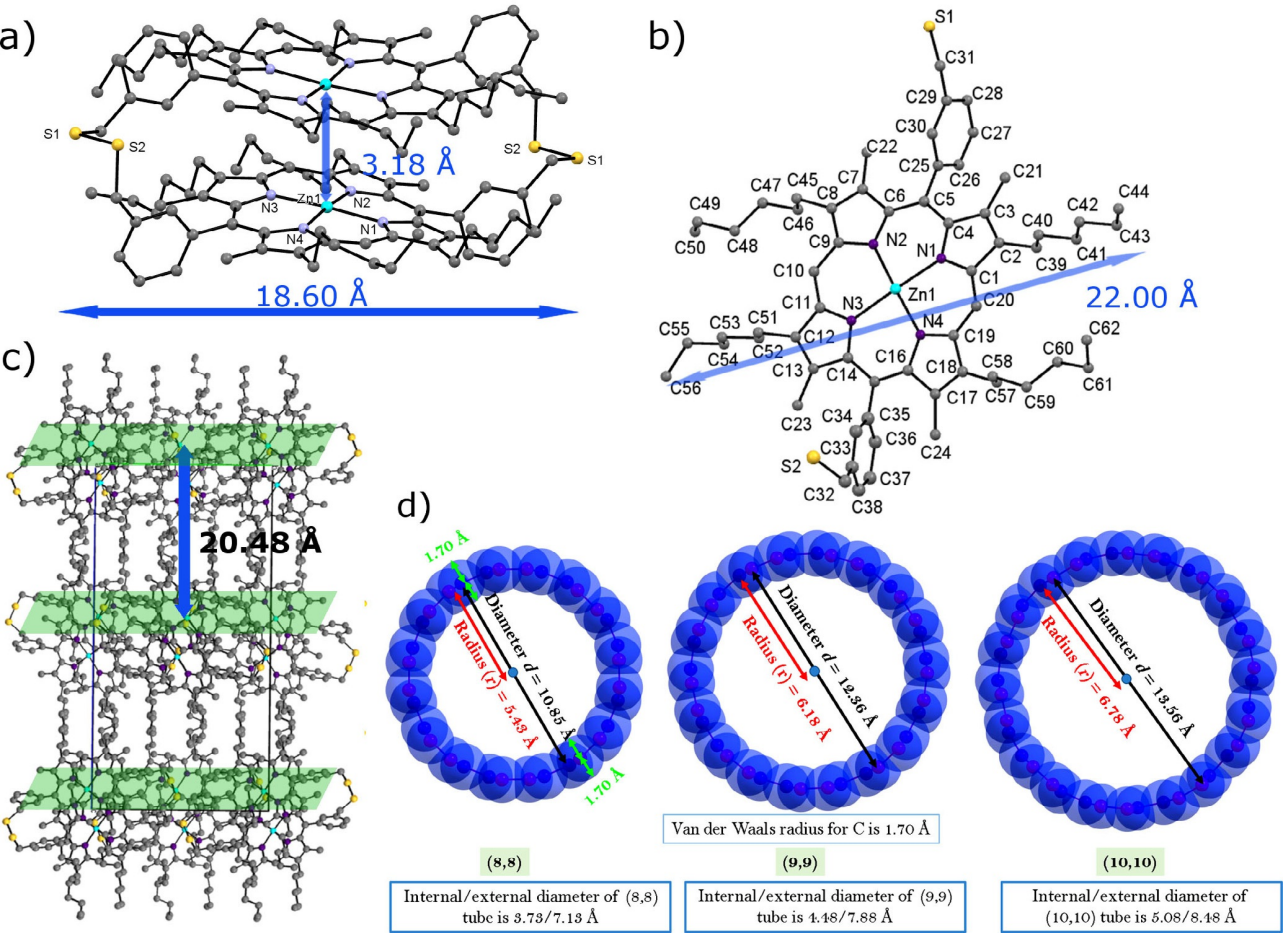
Molecular structure determinations by synchrotron single‐crystal X‐ray diffraction. a) The emerging side‐product Zn^II^‐porphyrin as a disulfide‐bridge dimer. b) Cell packing diagram of the Zn^II^‐porphyrin disulfide dimer, view over axis *a*. Hydrogen atoms were removed for clarity, c) DFT‐optimised structures of the SWNTs models (8,8), (9,9) and (10,10).

The self‐assembly approach provided a simple one‐step reaction method leading to the supramolecular adduct (**2**); the formation of the Zn^II^‐porphyrin@SWNTs adduct **2** was likely due to the aromatic stacking between the pyrrole rings of the Zn^II^‐porphyrin and the SWNT framework. During the synthesis, it emerged that the formation of disulfide‐bridged porphyrin oligomers is favourable over time in the presence of oxygen and in aqueous environments. A disulfide dimer side product featuring two S−S bridges and aromatically stacked porphyrins was isolated and fully characterised (Figure 4 and Supporting Information). Therefore, in the non‐covalent linked synthesis strategy, the thioacetate‐protected Zn^II^‐porphyrin was prepared and used in fresh solutions.

The directed assembly, leading to the disulfide‐bridged covalent linkages, was achieved as described in Figure 1. Spectroscopic investigations were carried out to probe whether or not this route can lead to a change of the carbon hybridisation in the outer wall of the carbon‐based material. The subsequent loss of the electronic conjugation, which affects the electron‐acceptor or the electron‐transport properties, was an expected feature of this approach.22i To achieve a covalent linkage between SWNTs and the complex **3**, a four‐step path reliant on the dynamic exchange, known to occur between thiol‐disulfides, was devised (Figure 1).

Chemical modifications of both compound **3** and SWNTs were introduced prior to the thiol‐disulfide reaction, which aimed to allow the control of the porphyrin position on the surface of the SWNTs (Figure 1); i) SWNT cyclopropanation reaction was achieved in the presence of diethyl bromomalonate and 1,8‐diazabicyclo[5.4.0]undecene (DBU). The resulting mixture led to (outer‐surface) modified SWNTs (**4**). ii) SWNT strands were further derivatised by treating **4** with 2‐mercaptoethanol, which acts as both linear spacer and thiol‐precursor linker. iii) Zn^II^‐porphyrin (**1**) was deprotected by treatment with excess hydrazine, to obtain the thioacetyl‐deprotected Zn^II^‐porphyrin (**3**). iv) Compounds **3** and **5** were assembled, aiming to covalently couple their thiol appendages in the presence of DBU, thus yielding the novel nanohybrid disulfide‐bridged adduct **6**.

### Microscopic investigation of the SWNT hybrids by AFM and TEM

**Figure 5:**
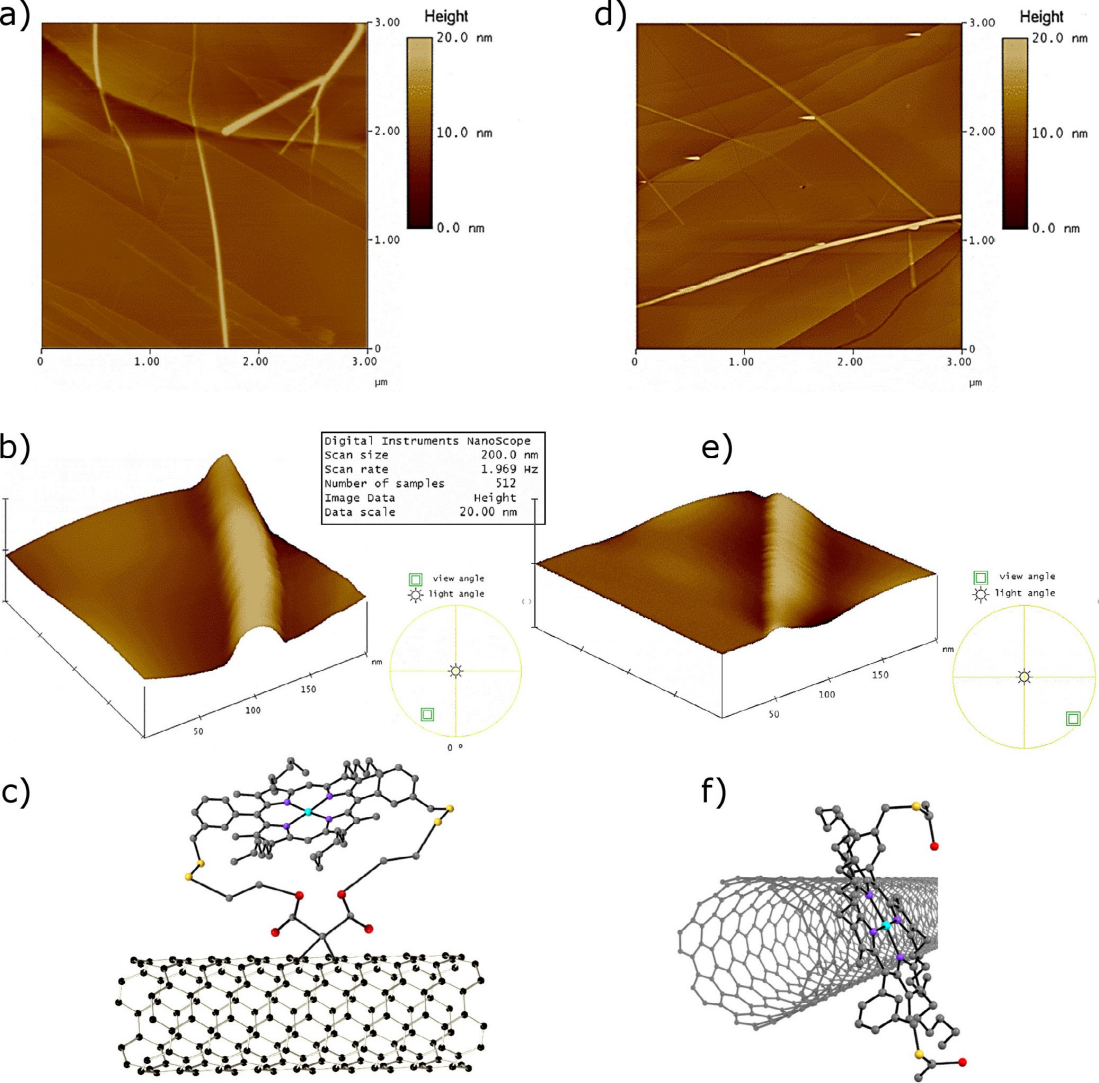
TM AFM imaging on mica surfaces. a, b) Image and 3D representation of AFM image of covalent Zn^II^‐porphyrin@SWNTs nanohybrids (**6**). c) Schematic diagram of compound **6**; d, e) Image and 3D representation of non‐covalent Zn^II^‐porphyrin@SWNTs nanohybrids (**2**). f) Schematic diagram of compound **2**.

The morphological properties of the two products, denoted as complexes **2** and **6**, were evaluated and compared by tapping mode AFM (TM AFM) and TEM. To investigate the different electron‐acceptor or electron‐transport properties in covalently and non‐covalently linked Zn^II^‐porphyrin@SWNTs complexes, UV/Vis, steady‐state time‐resolved fluorescence emission studies, together with Raman spectroscopy, were performed. TM AFM measurements were carried out to further study the SWNT nanohybrids and compare their aggregation with that observed in Zn^II^‐porphyrins. Interestingly, for SWNTs, HOPG (highly‐ordered pyrolytic graphite) worked best as the substrate of choice for AFM images (see the Supporting Information for details). The AFM images in Figure 5 a, b show that the covalent Zn^II^‐porphyrin@SWNTs nanohybrid is relatively well dispersed in solution with a diameter of roughly 11 nm. The AFM images in Figure 5 d, e indicate that the non‐covalent Zn^II^‐porphyrin@SWNTs nanohybrids are roughly 9 nm in diameter. The slight difference in diameter between the non‐covalently linked and covalently linked nanohybrids is likely a direct consequence of the nature of the bond between the two components of the complex (porphyrin and SWNTs), as well as the presence or absence of a linker. This generated a substantial morphologic variation, highlighting the significance of choosing a covalent or supramolecular approach for SWNT functionalisation. Such differences observed in the estimated diameters of the nanohybrids (**2** vs. **6**) could be assigned to the following: i) the different surface arrangement of the Zn^II^‐porphyrin on the surface of SWNTs in the non‐covalently linked complex, ii) the plane of the porphyrin containing the four pyrroles rings is parallel to the principal axis of the SWNT and iii) the aromatic ring‐stacking interactions maintain the porphyrin linked to the SWNT. Within the covalently linked complex **6**, the plane of the porphyrin is likely held at a longer distance with respect to the principal axis of the SWNT compared to the case of **2**. However, the diameter differences observed between the two specimens may also be due to several SWNTs assembled together and thus forming bundles.

**Figure 6:**
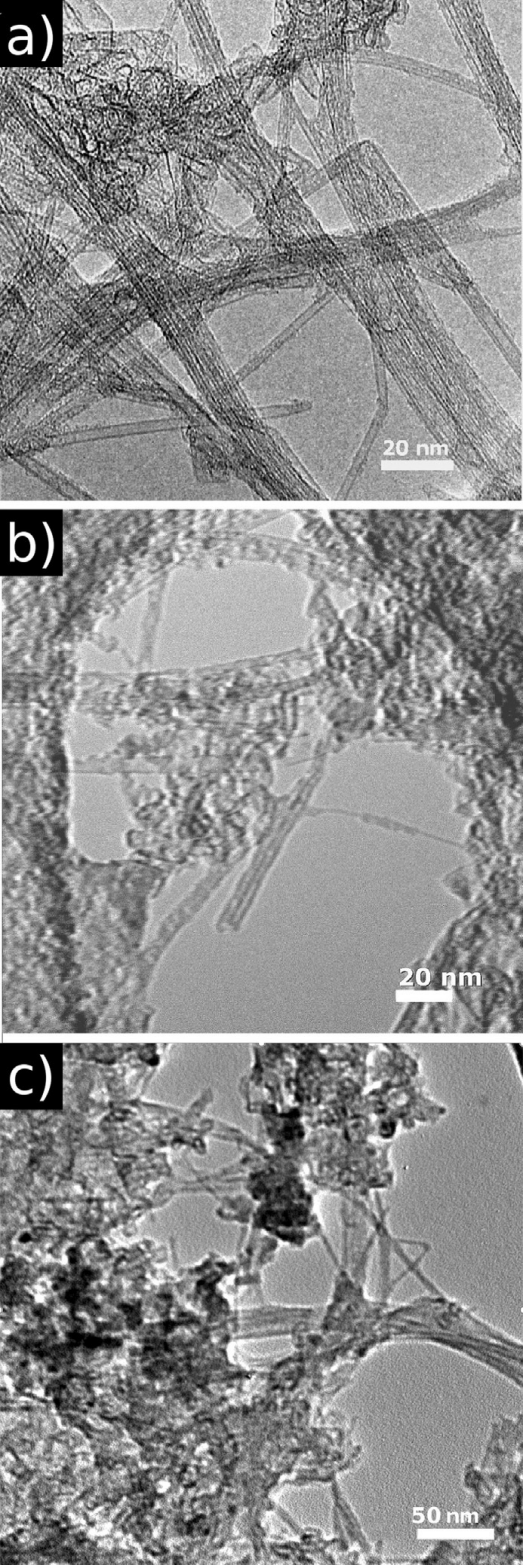
TEM microscopy of a) the free pristine SWNTs, b) non‐covalent Zn^II^‐porphyrin@SWNTs nanohybrids (**2**) and c) covalent Zn^II^‐porphyrin@SWNTs nanohybrids (**6**).

TEM measurements were employed to further characterise the morphology of SWNTs. Figure 6 b shows a TEM image of the non‐covalent Zn^II^‐porphyrin@SWNTs complex **2**. Dark areas with stronger contrast arise due to the presence of zinc, while the brightest sites of interest correspond to areas with only lighter elements such as hydrogen, carbon and nitrogen, consistent with the EDS analyses of different areas of interest. Figure 6 b shows that the SWNT have a rough surface as well as aggregates formed by porphyrin nano‐crystallization. The significant presence of a surface‐roughened SWNTs indicates coverage of the surface, suggesting the presence of the Zn^II^‐porphyrin on the nanotube surface.^23^

The TEM image of the covalent Zn^II^‐porphyrin@SWNTs complex **6** (Figure 6 c) shows a rather homogenous distribution of the complex over the surface of the SWNTs with regular aggregation points (areas with stronger contrast) and a lower agglomeration of the SWNTs was observed for **6** versus **2**. These subtle differences in morphology observed in the micrographs of **2** and **6** might be explained by the covalent bond of the Zn^II^‐porphyrin to the SWNTs, which strongly immobilises the molecules, reducing their agglomeration and improving their homogenous distribution over the surface of the SWNTs. In complex **2**, the donor–acceptor nature of the non‐covalent bond between Zn^II^‐porphyrin and SWNTs allows for a higher degree of porphyrin mobility onto the sp^2^ surface. Therefore, a non‐homogeneous distribution of the donor molecules onto the acceptor surface can facilitate the aggregation of porphyrin molecules. AFM and TEM (both deemed reliable techniques to assess the morphology of materials produced by chemical modification of the surface of SWNTs) confirm the formation of isolated individual SWNTs from bundled ropes upon derivatisation.22i

Both covalently and non‐covalently linked Zn^II^‐porphyrin@SWNTs nanohybrids were found to be relatively well dispersed in organic solvents upon specimen preparation for microscopies. Pristine SWNTs were found to be more difficult to disperse in solution even after prolonged treatment of sonication, and also the intact SWNTs were observed to be aggregated on the highly ordered pyrolytic graphite (HOPG) substrate. A similar conclusion regarding aggregation behaviour can be derived from absorption spectroscopy in the dispersed phase (see below).

### Surface derivatisation studies on SWNT hybrids by Raman and XRD spectroscopy

Raman spectroscopy was applied to evaluate the bulk properties and probe the degree of crystallinity of the graphitic carbon structure, including whether or not this structure remained intact following extensive synthetic chemical manipulations.^24^

**Figure 7:**
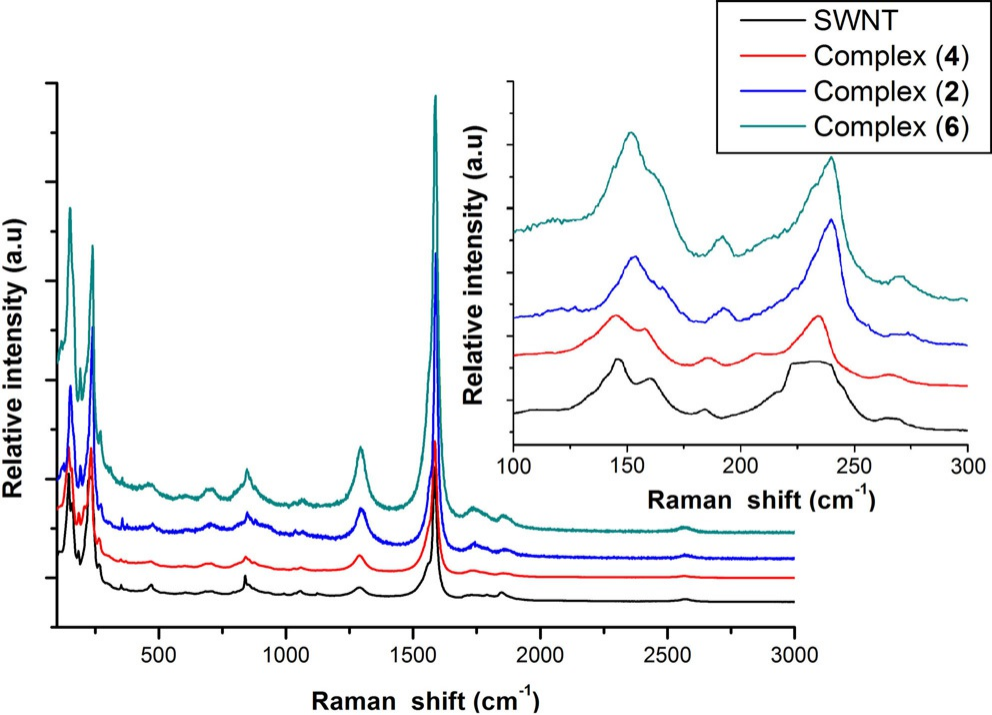
Solid state Raman spectroscopy of SWNT (830 nm), Bingel reaction purified SWNT (**4**), non‐covalently linked Zn^II^‐porphyrin@SWNTs complex (**2**) and covalently linked Zn^II^‐porphyrin@SWNTs complex (**6**); Inset: Raman spectrum RBM (radial breathing modes).

The Raman spectra shown in Figure 7 were recorded by dispersing samples in CHCl_3_/EtOH (1:1) using the excitation wavelength *λ*_ex_=830 nm. Spectra showed the G band at roughly 1581 cm^−1^ (primary graphitic mode), corresponding to a splitting of the E_2g_ stretching mode of graphite, reflecting the presence of sp^2^‐hybridised carbons. The Raman spectra of SWNTs **4** exhibit a G band at 1585 cm^−1^ and a D band at 1289 cm^−1^. The *I*_D_/*I*_G_ band intensity ratio of covalently functionalised SWNTs **4** (0.16) increased somewhat compared to the *I*_D_/*I*_G_ band intensity ratio of primitive SWNTs (0.11), indicating that the Bingel reaction introduced surface defects and disordered graphite structures onto the SWNTs. It can be seen that the Raman spectra of complex **2** shows a G band at 1588 cm^−1^ and D band at 1292 cm^−1^. A 0.17 *I*_D_/*I*_G_ band intensity ratio was also observed form the Raman spectra of complex **2**. Compared to complex **2**, the Raman spectra of the covalent Zn^II^‐porphyrin@SWNTs complex **6** exhibits G and D bands at 1588 cm^−1^ and 1293 cm^−1^, respectively. Moreover, the *I*_D_/*I*_G_ band intensity ratio slightly increases to 0.19. Compared to the *I*_D_/*I*_G_ band intensity ratio of free SWNTs, the ratio of both complexes **2** and **6** shows an increase, which suggests that a supramolecular interaction between the SWNTs and **1** has occurred. Raman spectroscopy also showed peaks located between 140–300 cm^−1^, which correspond to radial breathing modes (RBM; Figure 7 inset). The free SWNTs contain tubes with a broad diameter ranging 0.8–1.8 nm, while there are two significant and sharp RBM peaks at 234 cm^−1^ and 146 cm^−1^, which indicate that there are tubes with diameters of 1.02 nm and 1.64 nm. The spectra recorded for the covalently functionalised SWNTs in complex **4** remained largely unchanged with respect to free SWNTs, which exhibit two maxima RBM peaks at 234 cm^−1^ and 145 cm^−1^, suggesting that the diameters of the nanotubes are between 1.02 nm and 1.64 nm.^25^ The non‐covalent Zn^II^‐porphyrin@SWNTs complex **2** shows maximum RBM peaks at 240 cm^−1^ and 154 cm^−1^. This indicates that dispersions of porphyrin **2** in a mixture of CHCl_3_/EtOH (1:1) contains tubes with diameters of 0.99 nm and 1.55 nm. The RBM frequencies of **6** (240 cm^−1^ and 152 cm^−1^) suggest that carbon nanotubes with a diameter of 0.99 nm and 1.57 nm are mainly present.

To verify the nature of the species adsorbed onto the surface of the SWNTs and the nature of their possible binding modes, XPS measurements were carried out. In particular, the C1s, O1s and N1s core levels were evaluated for both Zn^II^‐porphyrin@SWNTs nanohybrids and pristine SWNTs.

**Figure 8:**
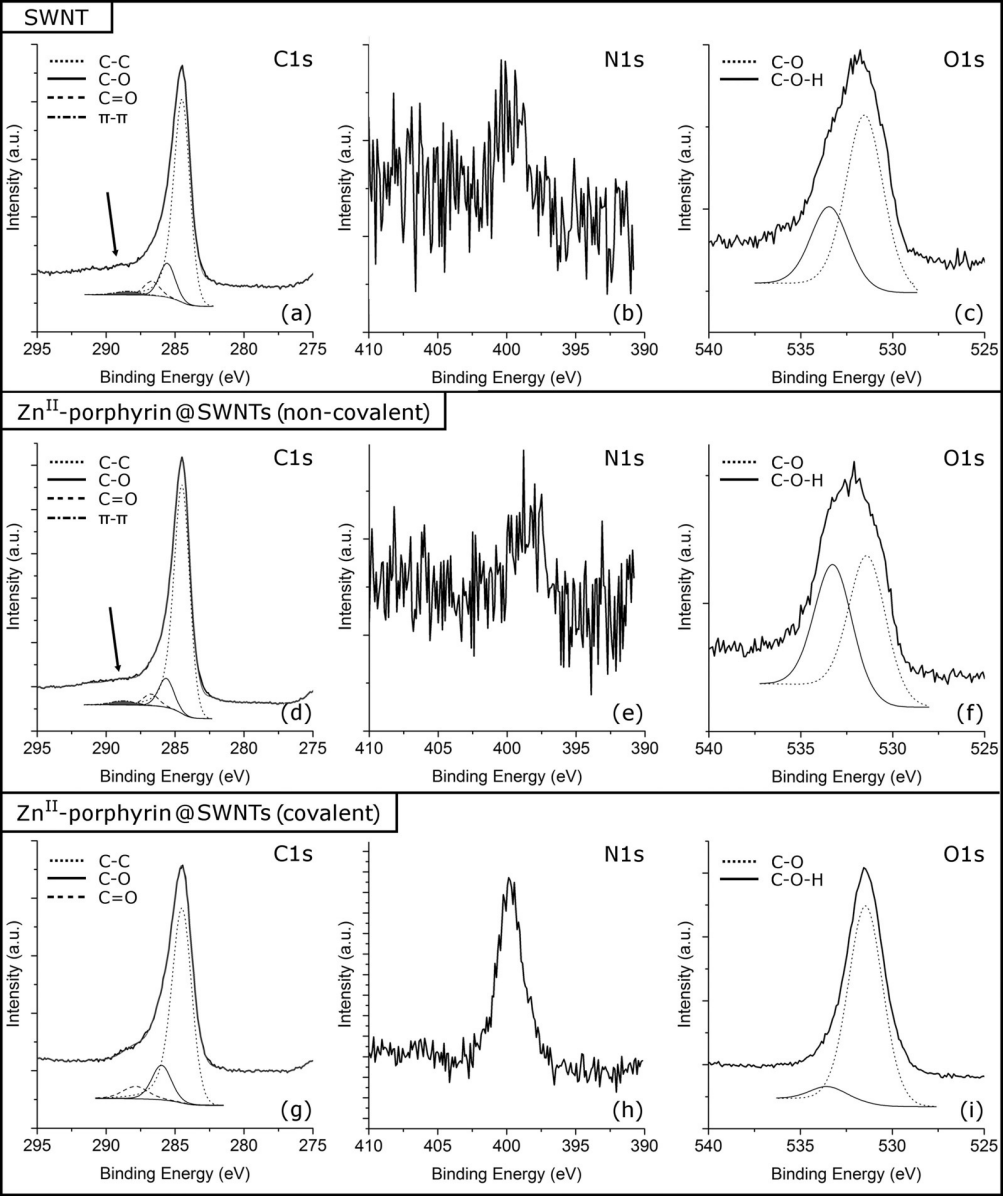
XPS spectra corresponding to: a) C1s, b) N1s and c) O1s regions for the SWNT; d) C1s, e) N1s and f) O1s regions for the non‐covalent Zn^II^‐porphyrin@SWNTs hybrid; g) C1s, h) N1s and i) O1s regions for the covalent Zn^II^‐porphyrin@SWNTs hybrid.

Figure 8 a, d, g show the XPS spectra corresponding to the C1s level and, as observed, C−C, C−O and C=O convoluted components can be observed in SWNTs and both non‐covalent Zn^II^‐porphyrin@SWNTs **2** and covalent Zn^II^‐porphyrin@SWNTs **6** (in SWNTs the C−O and C=O signals come from the carbonated surface; in the Zn^II^‐porphyrin@SWNT samples they arise from both the carbonates and the C−O groups). However, there is one additional signal in the SWNT and non‐covalent Zn^II^‐porphyrin@SWNT **2** systems, a weak shoulder located at a binding energy slightly above that of C=O (arrow marked in Figure 8 a, d). This signal can be associated with the existence of π–π interactions between the SWNTs and/or the Zn^II^‐porphyrins, for example, non‐covalent bonding. The lack of this component in the Zn^II^‐porphyrin@SWNT (covalent) suggests that the presence of Zn^II^‐porphyrin systems covalently linked to the surface of the SWNTs avoid the π–π interactions between the SWNTs. Figure 8 **b**, **e, h** show the XPS spectra corresponding to the N1s core level of the samples. Only in the case of the Zn^II^‐porphyrin@SWNT (covalent) system, an additional signal can be observed at 399.7 eV, which can be ascribed to an N−C chemical environment. This is indicative of the presence of Zn^II^‐porphyrin at the SWNTs surface, as expected. However, the absence of this signal in the spectrum of Zn^II^‐porphyrin@SWNT (non‐covalent) Figure 8 e and the similarity between the C1s spectra of pristine SWNTs and Zn^II^‐porphyrin@SWNT (non‐covalent) can be ascribed to the formation of Zn^II^‐porphyrin aggregates and the heterogeneous distribution of the Zn^II^‐porphyrin molecules. This last feature is in strong agreement with the TEM and AFM results. Finally, Figure 8 c, f, i show the O1s spectral decomposition. In all cases, C−O and C−O−H convoluted components can be observed, showing that the covalent Zn^II^‐porphyrin@SWNT sample has less C−O−H species at the SWNT surface with respect to the C−O species. On the other hand, the non‐covalent Zn^II^‐porphyrin@SWNT presents the highest level of this species indicating a more hydroxylated surface of the SWNTs.

### DFT calculations

To understand further the geometry adopted by an isolated Zn^II^ porphyrin molecule bound non‐covalently onto a single model SWNT strand and the nature of its interaction with this aromatic “guest”, DFT calculations using CASTEP^26^ code were performed. A model fragment of a single‐strand‐capped [10,10] nanotube was selected (as this is 1.6 nm wide), consistent with the expectations from Raman spectroscopy, and its ability to bind supramolecularly with the porphyrin molecule **1** was estimated using a gas phase modelling approach that has been successfully applied in previous studies.11c, 27 One of our previous models considered a composite formed between a tripodal porphyrin trimer unit and a capped [10,10] SWNT11c, whereby two of the three porphyrin units were found to interact closely with the surface of the SWNT. In that initial model, the binding energy was calculated to be −2.046 eV (−1.023 eV per porphyrin unit) and the charge transfer was estimated to be 0.003 electrons, clearly indicating the non‐covalent nature of the binding. In a related “host–guest” approach, but involving a different chromophore, a napthalenediimide molecule was allowed to bind supramolecularly onto the middle part and tip of a [10,10]‐capped SWNT and their binding energies were −0.84 eV and −0.66 eV, respectively.^27^

**Figure 9:**
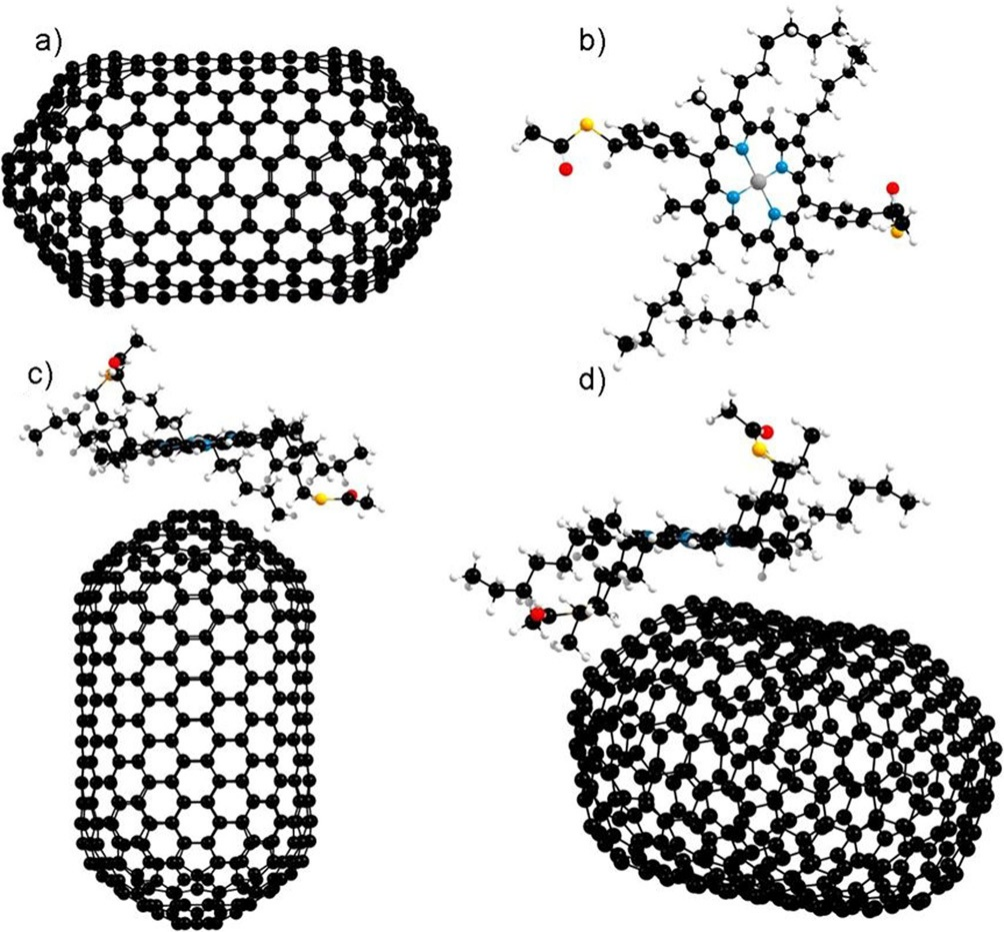
DFT‐optimised structures of model compounds. a) Short [10,10]‐capped SWNT; b) zinc(II)‐centred porphyrin molecule; c) porphyrin molecule bound supramolecularly onto the tip of a [10,10]‐capped SWNT (configuration **A**); d) porphyrin molecule bound supramolecularly onto the middle part of a [10,10]‐capped SWNT (configuration **B**). Black carbon, red oxygen, blue nitrogen, yellow sulfur, white hydrogen, zinc grey.

The magnitude of the binding energy depends on the type of the small molecule involved and the level of theory adopted. The amount of charge transferred in the porphyrin–SWNT model used hereby was calculated to be 0.044 electrons in the tip‐binding configuration and 0.086 electrons in the middle‐binding configuration, again showing the non‐covalent interaction. In the model reported hereby, the occurrence of van der Waals (vdW) interactions was considered. As this interaction is attractive, our calculated binding energy is slightly higher as expected. However, calculated binding energies and the amount of charge transferred in the model reported hereby are consistent with our previous models. In the literature a very few computational studies are available on single porphyrin units absorbed on SWNTs.^28^ Basiuk et al. have studied the non‐covalent functionalisation of carbon nanotubes with porphyrin theoretically.^29^ Figure 9 shows the DFT‐.relaxed geometries of model compounds used in this study. It is likely that the aromatic stacking of the porphyrin–SWNTs alters the optical properties of the emerging nanocomposite and ensures the preservation of a non‐covalent monomeric porphyrin unit ready for a controlled donor–acceptor interlayer association with SWNTs. From the dimer structure determinations by synchrotron single‐crystal X‐ray diffraction, it can be seen that the size of the Zn^II^‐porphyrin measured between the methyl groups of the phenyl rings is 16.55 Å. This value is larger than the calculated inner space for [8,8], [9,9] and [10,10] SWNTs (Figure 9 d). Therefore, all of the porphyrin molecules will bind to the outside of the SWNTs and interact with their outer surface.

The calculated binding energies for the porphyrin molecule interaction with the tip of the SWNT (configuration **A**) and the sidewall of the SWNT (configuration **B**) are −0.69 eV and −1.55 eV, respectively, indicating a π‐orbital interaction between the porphyrin molecule and the SWNT. The negative binding energies indicate that both complexes are thermodynamically stable. Total energies calculated for each system and the methodology we adopted to calculate the binding energy are given in the Supporting Information. Mulliken population charge analysis was carried out to estimate the charge transfer between porphyrin and SWNT. Calculations showed that for a model composite with configuration **A** (tip‐binding geometry), 0.04 electrons were transferred between a molecule of porphyrin and the [10,10] SWNT. For configuration **B** (in which a porphyrin molecule is bound to the middle of the SWNT), 0.08 electrons were transferred locally between one porphyrin unit and the small model of a carbon nanotube considered.

The DFT‐relaxed model used for computational studies considered only a very short length for the SWNT modelled (200 nm); this allows a maximum of four porphyrin molecules to bind in configuration **A** (coating the tip of the SWNTs) and a further six porphyrin molecules to bind in configuration **B** (to the middle part of the SWNT). This DFT gas‐phase model estimates that approximately two electrons are transferred between ten molecules of porphyrin absorbed onto the outer surface of a 500 nm length of a capped SWNT fragment. This electron transfer can be attributed to the donor–acceptor interaction between a porphyrin molecule and SWNT. DFT calculations were employed to obtain a direct view of the equilibrium geometry and the electronic structures for the frontier orbitals of the Zn^II^‐porphyrins. The crystal structures of the above complexes were used as an input geometry for gas phase optimisation by B3LYP 6‐31G(d,p), whereby a restricted spin was used for the optimisation of the diamagnetic zinc(II) porphyrin complex.

**Figure 10:**
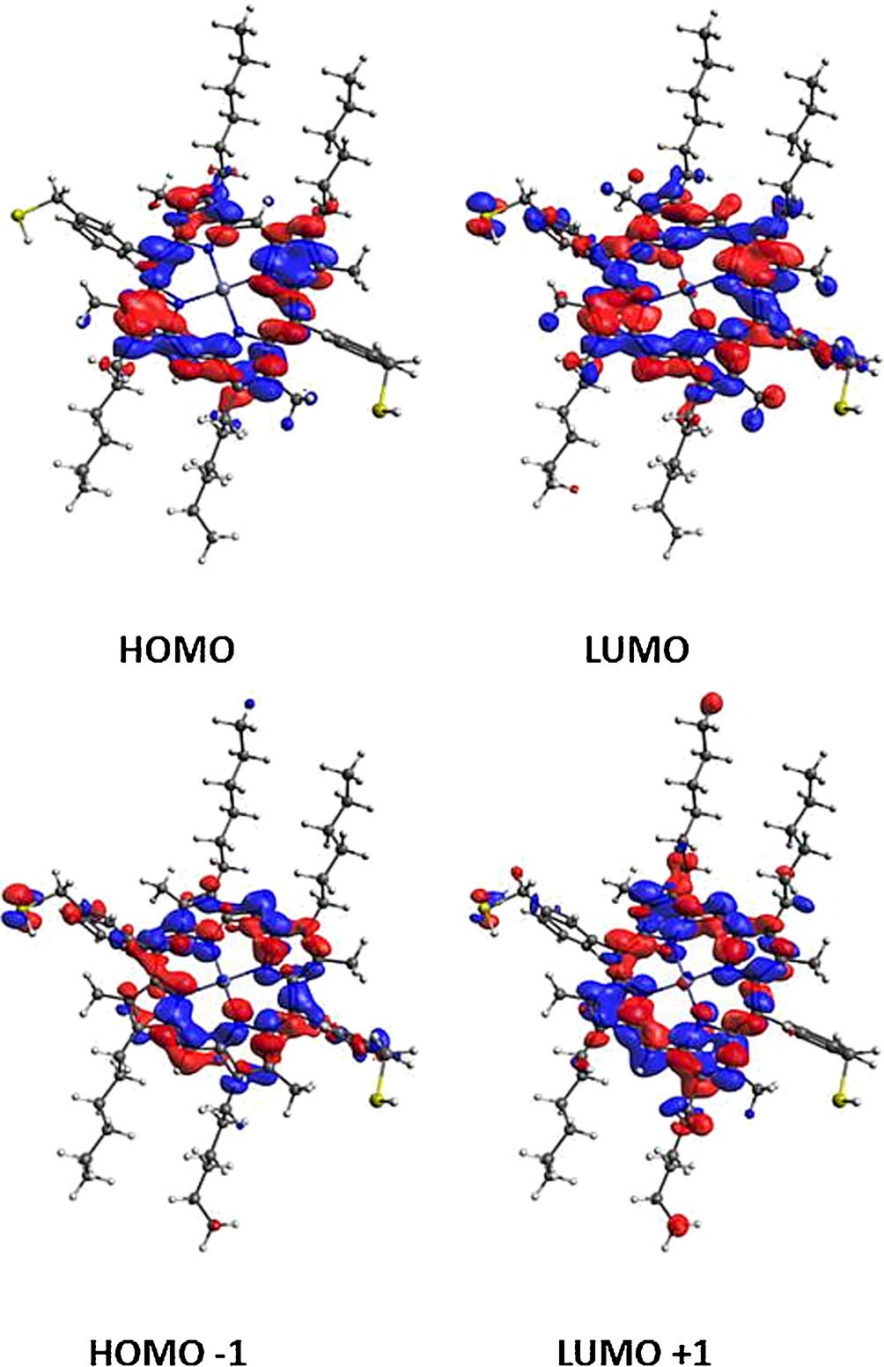
a) Two highest occupied and lowest unoccupied molecular orbital of de‐protected Zn^II^‐porphyrin (**3**) from gas phase DFT calculations.

The calculated electronic structures did not show negative frequencies, indicating that the optimised geometries are in the inclusive energy minima.^30^ Porphyrins with *D*_4*h*_ symmetry generally show energetically degenerate two lowest unoccupied molecular orbitals (LUMO+1, LUMO) and nearly degenerate two highest occupied molecular orbitals (HOMO, HOMO−1), as shown in Figure 10. Table S3 (Supporting Information) shows the energy level of each molecular orbital. The calculated energy band gap for Zn^II^‐porphyrin was 3 eV, which indicates a visible light absorption and promising potential photovoltaic properties that could be applied in the design of solar cells. The energy gap is in accordance with results recently reported for Zn^II^‐porphyrin species.^31^ Together with the molecular orbital structure and the calculated energy level, it can be found that, after modification at both *meso*‐and β‐positions with aryl thioacetate‐functionalised side groups and hexyl chains, respectively, the areas of high electron‐density were moved from the macrocyclic ring (shown in HOMO) to *meso*‐ (as shown in LUMO) and β‐positions (as shown in LUMO+1). These results show that the substitutions at *meso*‐and β‐positions of the Zn^II^‐porphyrin mainly affect the energy levels for unoccupied orbitals. Since these molecular orbitals are involved in the interaction between ligand (including the phenyl substituent) and metal, it is highly likely that both contribute to the UV/Vis light absorption properties.

This observation may explain the observed similarity between the UV/Vis absorption bands in this family of compounds as well as the small metal dependency. Modelling results also explain the observation that the substitutions of the side groups and hexyl chains may play a dominant role in determining fast electron injection from the excited singlet state of Zn^II^‐porphyrin to other coordinate materials such as carbon nanotubes, graphene oxide or reduced graphene oxide. Such interactions may be of relevance in the current quest for new sustainable chemistry approaches to enhance the performances of solar cells and bio‐sensing devices.

UV/Vis spectroscopy was carried out to further investigate the optical absorption behaviour of nanocomposites **2** and **6** in dispersed phases. The supramolecular assembly of Zn^II^‐porphyrin and SWNTs was studied by using optical spectroscopy and relevant competition experiments (Figure S50 and corresponding experimental details are given in the Supporting Information). Figures S51 a, b (Supporting Information) shows the UV/Vis absorption spectra of a 1:1 orthodichlorobenzene (*o*‐DCB)/chloroform solution of Zn^II^‐porphyrin (3 mL, 1 μm) titrated with a 1:1 (*o*‐DCB)/chloroform mixture of SWNTs with the addition of Zn^II^‐porphyrin (**1**; 0.5 μm).

**Figure 11:**
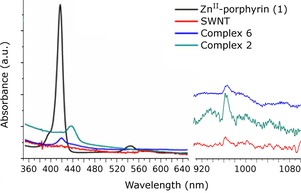
UV/Vis and UV/Vis‐NIR spectroscopy of Zn^II^‐porphyrin, intact SWNTs, non‐covalent Zn^II^‐porphyrin@SWNTs nanohybrids (**2**) and covalent Zn^II^‐porphyrin@SWNTs nanohybrids (**6**).

Figure 11 shows the optical absorption spectra of Zn^II^‐porphyrin, SWNTs, non‐covalent Zn^II^‐porphyrin@SWNTs complex (**2**) and covalent Zn^II^‐porphyrin@SWNTs complex (**6**). Zn^II^‐porphyrin (**1**) shows a main absorption peak at 417 nm, while the intact SWNTs do not show any obvious absorption band in UV/Vis spectral range. As the SWNTs are covalently linked to Zn^II^‐porphyrin, the resulting complex **6** shows a peak at 420 nm, slightly red‐shifted compared with the intact Zn^II^‐porphyrin (**1**). The Zn^II^‐porphyrin@SWNTs (non‐covalent) complex (**2**) shows a red‐shifted main absorption peak at 436 nm. These results suggest that the chromatic shifts of complex **2** and complex **6** are due to the nature of the interaction of Zn^II^‐porphyrin with the SWNTs. The covalent interaction between SWNTs and Zn^II^‐porphyrin seems to minimise the extent of the red shift observed. The UV/Vis‐NIR spectra of Zn^II^‐porphyrin@SWNT (recorded in pure CHCl_3_) shows the typical *S*_22_ transitions in the region around 960 nm. The *S*_22_ and metallic *M*_11_ transitions characteristic to SWNTs can be seen at 500–1000 nm in the spectrum of Zn^II^‐porphyrin@SWNTs. Since the transition energies are related to the size of the SWNT bundles, and the presence of isolated tubes in solution have been reported to give rise to sharp, well‐resolved peaks,^27^ UV/Vis‐NIR spectroscopy of the Zn^II^‐porphyrin@SWNT composite suggests that Zn^II^‐porphyrins are capable to efficiently attach to the surface of SWTNs. Due to the enhanced dispersibility caused by the attachment of Zn^II^‐porphyrin, this absorbance band may well constitute a further indication of the de‐bundling of the SWNT strands in solution.

**Scheme 1:**
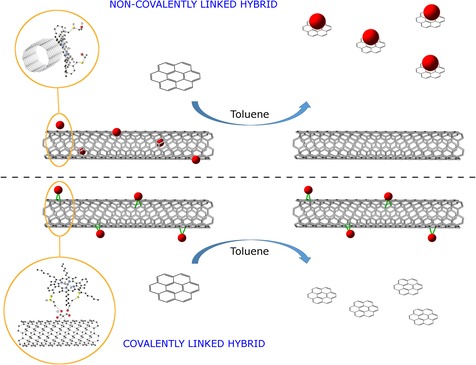
Addition of coronene (1 μm solution in toluene) and washing process of the suspensions of the non‐covalent and covalent nanohybrids Zn^II^‐porphyrin@SWNTs (**2**) and (**6**) in 1:1 toluene/chloroform.

The extent of a supramolecular interaction between aromatic molecules and carbon nanostructures has been previously studied by using a smaller fraction of a sp^2^ carbon system.^32^ To demonstrate how the covalent and non‐covalent linking strategies may differ in their effectiveness to connect the porphyrins to SWNTs, a small aromatic competitor molecule, coronene, was introduced in suspensions of covalent and non‐covalent Zn^II^‐porphyrin systems **2** and **6**. This was expected to induce the dissociation of the donor–acceptor complexes and to allow the monitoring of this process in the dispersed phase by absorbance spectroscopy. While the high affinity (binding constant) of coronene for the Zn^II^‐porphyrin molecules induces a competitive equilibrium that facilitates the dissociation of the non‐covalent Zn^II^‐porphyrin@SWNTs system and the formation of a new Zn^II^‐porphyrin@coronene complex, this type of complex cannot be generated when Zn^II^‐porphyrin molecules are covalently attached to the surface of the SWNTs. Coronene was chosen deliberately as the competitor molecule as it can act as a simple model for the larger carbon‐based materials, whilst providing an insight into the possible π–π interactions between porphyrin and CWNTs. It would also facilitate the evaluation of the degree of supramolecular aggregation (Scheme 1). Data fitting involved a 1:1 binding isotherm model and the association constant (*K*_1:1_) was estimated to be 4.62×10^4^ m ^−1^ (Supporting Information). Therefore, coronene is indeed capable of acting as a competitive candidate likely to disrupt the interactions between SWNTs and Zn^II^‐porphyrin (Scheme 1).

A recent report^12^ described the complexity of the non‐covalent interactions between aromatic electron‐poor molecules, such as functional naphthalenediimides (NDI), and planar carbon‐based materials, such as thermally reduced graphene oxide (TRGO), as well as the use of coronene to elucidate the intimate nature of the donor–acceptor system, but, to the best of our knowledge, such competitive experiment involving functional zinc porphyrins and SWNTs has not been reported thus far.

### Fluorescence spectroscopy in solution and fluorescence lifetime imaging microscopy (FLIM) on thin films

**Figure 12:**
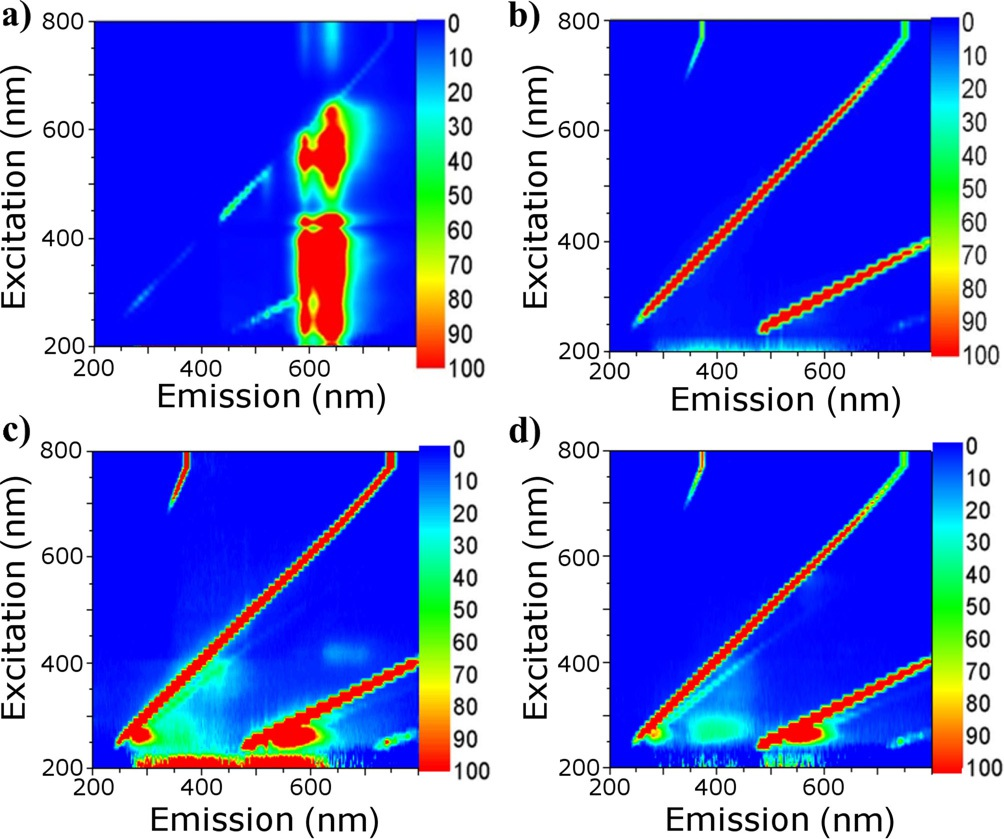
Two‐dimensional fluorescence contour plotting of a) Zn^II^‐porphyrin (1 μm, 1:1 ethanol/chloroform), b) SWNT (0.5 mg mL^−1^, 1:1 ethanol/chloroform), c) non‐covalent Zn^II^‐porphyrin@SWNTs complex (**2**, 1 mg mL^−1^, 1:1 ethanol/chloroform) and d) Zn^II^‐porphyrin@SWNTs (covalent) complex (**6**, 1 mg mL^−1^, 1:1 ethanol/chloroform).

To investigate the fluorescence emission behaviour of Zn^II^‐porphyrin and its covalent and non‐covalent hybrid of SWNTs, 2D fluorescence spectroscopy was performed. 2D fluorescence contour plotting of free Zn^II^‐porphyrin, SWNTs, non‐covalent Zn^II^‐porphyrin@SWNTs complex (**2**) and covalent Zn^II^‐porphyrin@SWNTs complex (**6**) were carried out in a 200–800 nm excitation–emission range. Free Zn^II^‐porphyrin (**1**) shows a strong emission peak between 570 nm and 670 nm (Figure 12 a). A strong Rayleigh scattering line is shown in the 2D contour plot of SWNTs (Figure 12 b), but no detectable fluorescence emission is observed from the SWNTs suspension. After porphyrin **1** was non‐covalently linked to the SWNTs surface by π–π stacking, the excitation–emission, observed for a Zn^II^‐porphyrin (**1**) solution, was remarkably quenched. Moreover, a blue shift of the wavelength of maximum emission can be observed from 570–670 nm to 530–610 nm (Figure 12 c). Such quenching behaviour and associated blue shift (from 570–670 nm to 520–590 nm) can also be found in the 2D fluorescence spectra of the covalently linked complex **6** (Figure 12 d). The intensity of the emission maximum for the covalently linked complex was observed to slightly decrease in comparison to the non‐covalently linked complex. The fluorescence quenching of the wavelength of maximum emission indicates that an energy transfer process from the Zn^II^‐porphyrin to the SWNTs has occurred, while the blue shift is generated due to the non‐covalent interaction of porphyrin with the carbon surface. These results confirm the non‐covalent adsorption interaction between Zn^II^‐porphyrin and SWNTs and, at the same time, the covalent nature of the porphyrin–SWNTs bond in the nanohybrid **6**.

TCSPC measurements were carried out to further investigate the excited state characteristics of Zn^II^‐porphyrin (Table 1 and Figure S46 in the Supporting Information). In solution studies, the TCSPC fluorescence decay data of compound **1** (two‐photon excitation at 810 nm) shows the presence of a major component with a short lifetime decay (*a*_1_=93.2 %, *t*_1_=39 ps) and a minor longer lived component (*a*_2_=6.8 %, *t*_2_=1483 ps, Table 1).

**Table 1:** Time‐correlated single‐photon counting (TCSPC) (two‐photon excitation, *λ*_ex_=810 nm) of Zn^II^‐porphyrin (1 μm in 1:1 DMF/toluene).^[a]^

	*χ* ^2^	*τ*_1_ [ps]	*a*_1_ [%]	*τ*_2_ [ps]	*a*_2_ [%]	*τ*_m_ [ps]
**1**	1.50	39±5.0	93.2	1483.5±2.8	6.8	136.67

[a] Laser power 5.4 mW.

**Figure 13:**
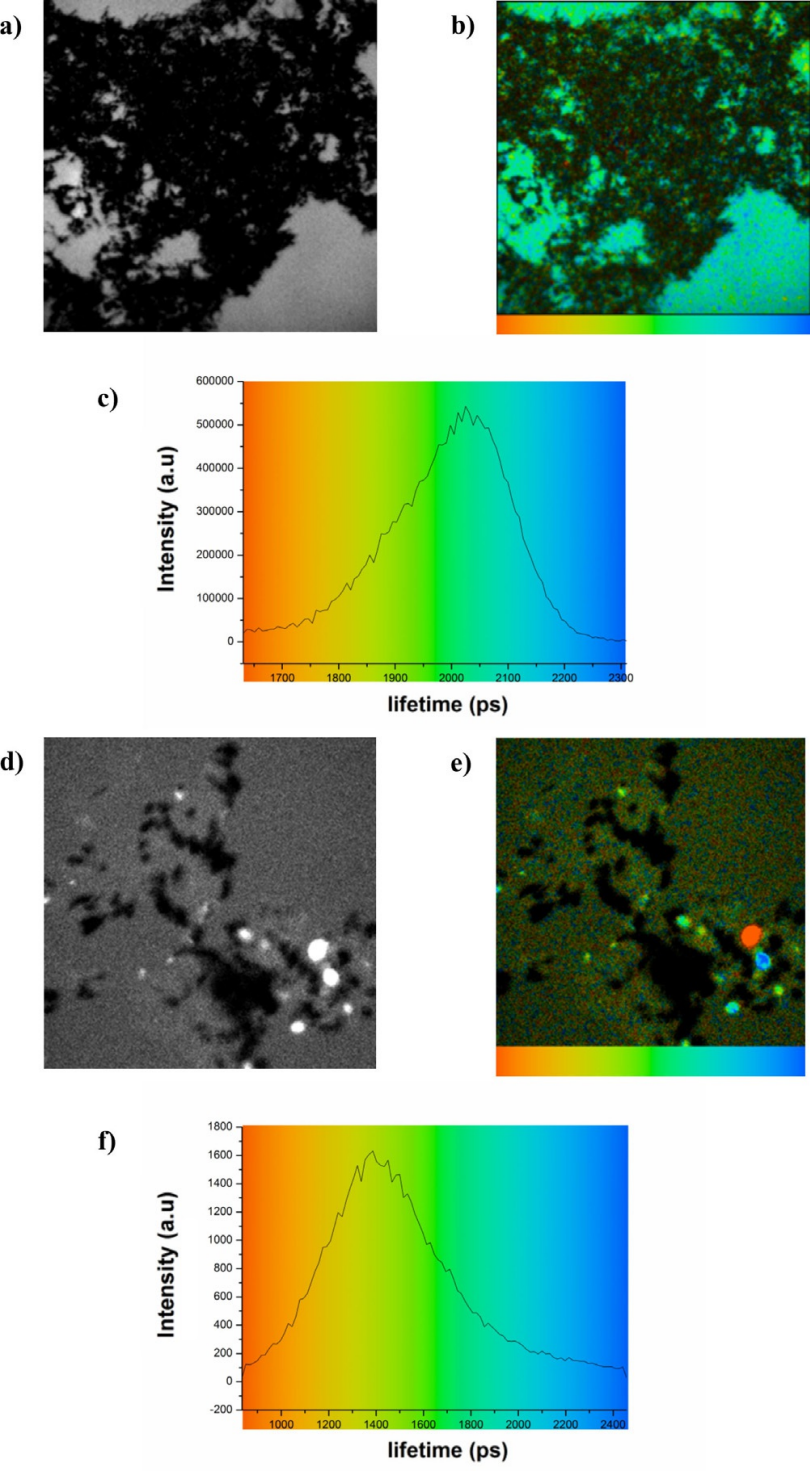
Single‐photon fluorescence lifetime map (*λ*_ex_=473 nm) and corresponding intensity images of a, b) solid non‐covalent Zn^II^‐porphyrin@SWNTs complex (**2**) and corresponding lifetime distribution curve (c) and d, e) solid covalent Zn^II^‐porphyrin@SWNTs (**6**) complex and corresponding lifetime distribution curve (f). Images (a, d) represent intensity images on thin films. b, e) A colour‐coded bar is provided for a direct correlation between the lifetime colour map (b, e) and lifetime histograms (c, f).

The interactions and consequent energy transfer between SWNTs and Zn^II^‐porphyrin were further investigated using laser‐induced single‐photon excitation and fluorescence emission excited state lifetime measurements When in solid state, the fluorescence decay of Zn^II^‐porphyrin@SWNTs could be fitted to a single component with a single lifetime of approximately 2000±100 ps following excitation at 473 nm (Figure 13 a, b). An additional relatively broad maximum in the lifetime distribution curve (Figure 13 c) was also observed. Such broad full width at half maximum (FWHM) suggests the presence of a heterogeneous fluorescence decay population and also confirmed the formation of various crystalline supramolecular assemblies and hybrid structures. Figures 13 d, e show lifetime emission maps of the solid‐state covalent Zn^II^‐porphyrin@SWNTs complex (**6**) using similar conditions to the measurement carried out for the non‐covalently linked complex **2**. Shown in Figure 13 f, the corresponding lifetime distribution curve proved a shorter lifetime measured in complex **6** (1400±100 ps). A broad FWHM of the corresponding lifetime distribution curve was also observed. The difference in major lifetime between the covalent complex **6** and the non‐covalent complex **2** also indicates that the two types of synthetic strategies result in different electronic interactions between the Zn^II^‐porphyrin and the SWNTs and different degrees of quenching.

**Figure 14:**
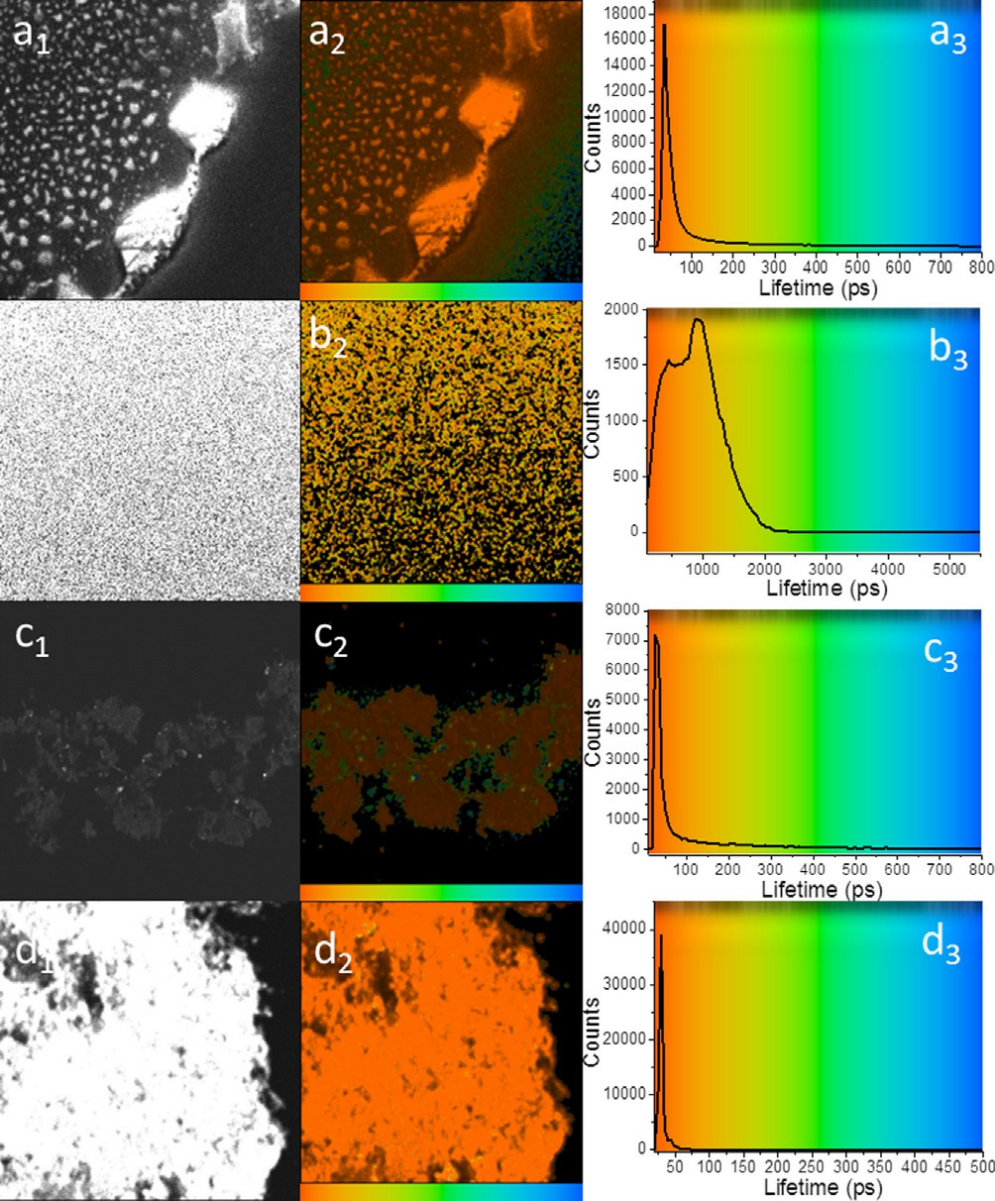
Two‐photon fluorescence lifetime map (*λ*_ex_=810 nm) of a_1_, a_2_) microcrystalline free‐base porphyrin and associated lifetime profile distribution across the image (a_3_), b_1_, b_2_) microcrystalline Zn^II^‐porphyrin complex and associated lifetime profile distribution (b_3_), c_1_, c_2_) non‐covalent solid Zn^II^‐porphyrin@SWNTs complex (**2**) and corresponding lifetime distribution curve (c_3_) and d_1_, d_2_) solid covalent Zn^II^‐porphyrin@SWNTs complex (**6**) and corresponding lifetime distribution curve (d_3_). Images (a_1_, b_1_, c_1_, d_1_) are two‐photon‐intensity images. A colour‐coded bar is provided for direct correlation between the lifetime colour maps (a_2_, b_2_, c_2_, d_2_) and lifetime histograms (a_3_, b_3_, c_3_, d_3_).

To further investigate the role of SWNT as a quencher of zinc and free‐base porphyrins, two‐photon FLIM for free‐base porphyrin and Zn^II^‐porphyrin, as well as solid complexes **2** and **6**, were carried out (Figure 14 a–d). For such measurements, two component systems (*τ*_1_ and *τ*_2_) were more accurate models of the decay profiles (Table 2 and Figure S38–S41 in the Supporting Information). Micro‐crystallites of free‐base porphyrin and Zn^II^‐porphyrin (**1**) imaged on borosilicate glass showed similar fluorescence decay profiles with *τ*_m_ (average lifetime) of 742±155.9 and 765±96.6 ps, respectively. Interestingly, the major component for the non‐covalent Zn^II^‐porphyrin@SWNTs complex (**2**) was significantly reduced (from 554±55.0 to 35±4.5 ps). The same shortening in lifetime can also be seen for the first component of the covalent complex **6**. In this instance, however, the extent of shortening is even more pronounced. In agreement to the observations for the single‐photon excitation mechanism, the covalent functionalisation of SWNTs results in a more significant lifetime shortening. The appearance of a second longer minor component suggests instead the presence of unquenched species free in solid state (free‐based or zinc porphyrins) or their supramolecular self‐assembled structures.^12^

**Table 2:** Solid state FLIM (fluorescence lifetime imaging microscopy) data for free‐base porphyrin precursor (denoted prec. in table), Zn^II^‐porphyrin (**1**), (non‐covalent) Zn^II^‐porphyrin@SWNTs complex (**2**) and (covalent) Zn^II^‐porphyrin@SWNTs complex (**6**).

	*χ* ^2^	*a*_1_ [%]	*τ*_1_ [ps]	*a*_2_ [%]	*τ*_2_ [ps]	*τ*_m_ [ps]
prec.	1.3	84.1	244±26.0	15.9	3369.7±460.1	742.0±155.9
**1**	1.17	87.3	554±55.0	12.7	1715.6±231.6	765.3±96.6
**2**	1.49	98.8	35±4.5	1.2	870.8±75.1	44.9±6.1
**6**	1.15	98.7	28±11.8	1.3	6880.1±228.0	120.5±67.1

We have recently reported that these changes in lifetime upon complexation, particularly the presence of a shorter lifetime component, may account for a photo‐induced excited‐state energy transfer occurring between the closely bound donor–acceptor aromatic chromophores and planar carbon‐based materials.^12^ Therefore, it is reasonable to conclude that a Förster resonance energy transfer (FRET) occurs in these systems, in which the porphyrins act as FRET donors and the SWNTs as the corresponding FRET acceptors.^12^, ^33^

## Conclusions

A multi‐pronged characterisation protocol involving imaging and spectroscopy methodologies probed the supramolecular properties of a zinc(II)‐substituted bulky aryl porphyrin. This tailored Zn^II^‐porphyrin synthon and pristine SWNTs were used as building blocks for new Zn^II^‐porphyrin@SWNTs adducts, which were either covalently or non‐covalently linked. The new nanocomposites materials have been synthesised by following either supramolecular self‐assembly by π–π stacking or by a disulfide linker bridging covalently the two components. The morphology and photochemical behaviour were thoroughly analysed by AFM, STED microscopy, TEM, XPS, Raman, UV/Vis and fluorescence spectroscopy, including two‐photon excitation techniques. Experiments showed that the Zn^II^‐porphyrin functionalisation is uniform and the two linking strategies also led to different morphologic characteristics for the functionalised SWNTs, as observed by AFM. The DFT calculations confirmed that the Zn^II^‐porphyrin of choice and its carbon monohybrids show potential to be applied in photovoltaics or/and bio‐sensing devices. For the first time, XPS was used to evaluate the presence of π–π interactions as an evidence of non‐covalent interaction between the porphyrin and the SWNT. UV/Vis and fluorescence spectroscopy and multi‐photon confocal fluorescence imaging were carried out to shed light into the nature of the interactions between the porphyrins and SWNTs scaffolds both in solution and in the solid state. The UV/Vis studies confirmed that the different synthetic strategies produced different light absorption properties. The fluorescence quenching and florescence lifetime suggests that a FRET mechanism between the Zn^II^‐porphyrin self‐assembled tubular aggregates (viewed as the FRET donor) and the SWNTs (FRET acceptor) occurs in the excited state. The donor–acceptor system designed hereby is predicted to show possible applications in the construction of novel optoelectronic devices using SWNTs as an electron acceptor. Interestingly, the non‐covalent linking strategies could help to mediate the absorption and emission properties of the original porphyrin. New kinetically stable complex systems emerged and this property is crucial for bio‐sensing applications. The directed, covalent linking strategy provided a strong and stable method to link small molecules and SWNTs, whereas in the case of the non‐covalently linked strategy, albeit very convenient and rapid, the porphyrin coating is detachable in the presence of other flat aromatic organic molecules capable of acting as competitors. This represents a challenge as well as an opportunity in designing functional nanohybrids for future sensing applications.

## Experimental Section

**Synthesis of Zn^II^‐porphyrin@SWNTs (non‐covalent) complex (2)**: Purified single‐walled carbon nanotubes (5 mg) were added to 30 mL of ethanol. The mixture was sonication for 10 min, three times with 5 min intervals, to make sure that the majority of the purified SWNTs were dispersed in the solution. The suspension was added to a 50 mL centrifuge tube and centrifuged at 3500 rpm for 30 min. The top‐layer supernatant was carefully separated and collected to afford a SWNTs ethanol solution. Afterwards, Zn^II^‐porphyrin (1 mg) was dissolved in chloroform (30 mL) and combined with the SWNTs solution. The mixture was left stirring for 24 h at room temperature, filtrated using a membrane and the remaining solids were rinsed with excess chloroform. The solid nanohybrids were collected and fully dried at 60 °C overnight.

**Bingel reaction for functionalised SWNTs**: For a typical reaction, the purified SWNTs (30 mg) were transferred to a quartz tube, which was afterwards annealed using a furnace under vacuum at 10^−3^ mbar at 1000 °C for 3 h under nitrogen. The annealed SWNTs were suspended in dry *ortho*‐dichlorobenzene (*o*‐DCB, 15 mL), by an ultrasonic treatment in a 80 W sonication bath for 15 min. Afterwards, diethyl bromomalonate (1.8 mmol) and 1,8‐diazabicyclo[5.4.0]undecene (DBU, 3.3 mmol) were added to the carbon nanotube suspension and the mixture was left stirring for 15 h under nitrogen at room temperature, followed by addition of trifluoroacetic acid (3.9 mmol) to quench the reaction. Afterwards, ethanol (35 mL) was added and the mixture transferred to a 50 mL centrifuge tube. The SWNTs were separated by centrifugation at 3500 rpm for 30 min. The pellet of SWNTs was washed several times with ethanol by repeating this centrifugation process. The SWNTs were then collected by a nano‐filtrate system and rinsed with more excess ethanol to fully remove the organic phase. The sample was dried at 60 °C overnight.

**Thiol‐SH deprotection of Zn^II^‐porphyrin**: Zn^II^‐porphyrin (3 mg) was dissolved in degassed CH_2_Cl_2_ (5 mL), then hydrazine monohydrate (200 μL) was added. This solution was stirred under nitrogen overnight and the solvent removed under reduced pressure. The product was re‐dissolved in degassed CH_2_Cl_2_ (10 mL ) and washed twice with degassed water (20 mL) under nitrogen. The organic phase was dried over sodium sulfate and the solvent was removed under reduced pressure to afford SH‐deprotected Zn^II^‐porphyrin.

**Synthesis of Zn^II^‐porphyrin@SWNTs (covalent) complex (6)**: Bingel reaction functionalised SWNTs were trans‐esterified by prolonged stirring in excess 2‐mercaptoethanol (5 mL) for 72 h under nitrogen. Afterwards, the products [(COOC_2_H_4_SH)_2_C<SWNTs] were extensively washed with diethyl ether (80 mL) and separated by filtration. The trans‐esterified SWNTs (1 mg) were added to a solution of SH‐deprotected porphyrins in chloroform (5 mm) and then a 10 mL of a 2:98 DBU/chloroform mixture was added dropwise (0.2 %). The solution was stirred for 72 h at room temperature under nitrogen. Afterwards, the mixture was filtrated and the solids were rinsed with excess ethanol. The resulting nanohybrid (black powder) was collected, transferred to a 30 mL glass vial and dried at 60 °C, overnight.

**DFT calculations**: The calculations to study the equilibrium geometry and electronic structures for the frontier orbitals of the Zn^II^‐porphyrins were first performed using DFT as employed in Gaussian09, using B3LYP 6‐31G(d,p).^34^ This was carried out in the High Performance Computing Facility, Aquila, University of Bath. To determine the structure of the complex formed and the nature of the interaction between the Zn^II^‐porphyrin and a carbon nanotube, DFT calculations were performed using CASTEP code,^26^ which solves the standard Kohn–Sham (KS) equations using plane wave basis sets. For the exchange correlation term the generalised gradient approximation (GGA) in the Perdew–Burke–Ernzerhof (PBE) parameterisation was employed.^35^ Ultrasoft pseudopotentials were generated using the “on the fly” formalism in CASTEP. A plane wave basis set with the energy cut‐off of 500 eV was used to expand the wave function. As the inclusion of van der Waals (vdW) interactions can improve the binding energy, in this work we have applied vdW corrections as implemented by Grimme^36^ in the CASTEP package. Structure optimisations were performed using BFGS algorithm and the forces on the atoms were obtained from the Hellman–Feynman theorem including Pulay corrections. In all optimised structures, forces on the atoms were smaller than 0.05 eV Å^−1^ and the stress tensor was less than 0.01 GPa. In all calculations, we used super cells with 40 Å, 40 Å and 40 Å vacuum spaces along *x*, *y* and *z* directions, respectively. This modelling method makes sure that the nanotubes plus functional groups do not interact with their periodic images. A single k point (*Γ*) was used in all calculations.

**Crystal structure determination**: Crystals of Zn^II^‐porphyrin dimer were grown from CHCl_3_ and MeOH mixtures. Crystal and structure refinement data are summarised in Table S1 in the Supporting Information. Data were collected at 180 K on a Nonius Kappa CCD with graphite‐monochromated Mo_Kα_ radiation (*λ*=0.71073 Å). Also, measurements on the same compound were performed at the synchrotron radiation source at Station 9.8, Daresbury SRS, UK, on a Bruker SMART CCD diffractometer (*λ*=0.69040 Å), which provided a higher quality data set. In both cases, the same cellular parameters were obtained and the structures were solved by direct methods using the program SIR92.^37^ The refinement and graphical calculations were performed on the synchrotron data set using the CRYSTALS^38^ and CAMERON software packages. The structures were refined by full‐matrix least‐squares procedure on *F*. All non‐hydrogen atoms were refined with anisotropic displacement parameters. Hydrogen atoms were located in Fourier maps and their positions adjusted geometrically (after each cycle of refinement) with isotropic thermal parameters. Chebychev weighting schemes and empirical absorption corrections were applied.^39^

**Synchrotron crystal data**: C_124_H_156_N_8_S_4_Zn_2_ *M*=2017.59; *Z*=4; orthorhombic; space group *Pbca*; *a*=17.629(5) Å, *b*=17.624(5) Å, *c*=34.458(5) Å; *α*, *β*, *γ*=90 ^o^; *V=*10706(5) Å^3^; *T*=150(2) K; *μ*=0.535 mm^−1^; 31 004 reflections measured; 6174 unique reflections (*R*_int_=0.047); 20 768 reflections with *I*>3 s (*I*); *R*=0.1587 and *wR*=0.1892. CCDC 1475150 contain the supplementary crystallographic data for this paper. These data are provided free of charge by The Cambridge Crystallographic Data Centre.

**Fluorescence lifetime measurements**: An optical parametric oscillator was pumped by a mode‐locked Mira titanium sapphire laser (Coherent Lasers Ltd), generating 180 fs pulses at 75 MHz and emitting light at a wavelength of 580–630 nm. The laser was pumped by a solid state continuous wave 532 nm laser (Verdi V18, Coherent Laser Ltd), with the oscillator fundamental output of 473±2 nm or 405±2 nm. The laser beam was focused to a diffraction limited spot through a water immersion ultraviolet‐corrected objective (Nikon VC x60, NA1.2) and specimens illuminated at the microscope stage of a modified Nikon TE2000‐U with UV transmitting optics. The focused laser spot was raster‐scanned using an XY galvanometer (GSI Lumonics). Fluorescence emission was collected without de‐scanning, bypassing the scanning system, and passed through a coloured glass (BG39) filter. The scan was operated in normal mode and line, frame and pixel clock signals were generated and synchronised with an external fast microchannel plate photomultiplier tube used as the detector (R3809‐U, Hamamatsu, Japan). These were linked by a time‐correlated single photon counting (TCSPC) PC module SPC830 operating either under single‐ or two‐photon excitation conditions. Lifetime calculations were obtained using SPCImage analysis software (Becker and Hickl, Germany) or Edinburgh Instruments F900 TCSPC analysis software.

## Conflict of interest

The authors declare no conflict of interest.

## Supporting information

As a service to our authors and readers, this journal provides supporting information supplied by the authors. Such materials are peer reviewed and may be re‐organized for online delivery, but are not copy‐edited or typeset. Technical support issues arising from supporting information (other than missing files) should be addressed to the authors.

Supplementary
