# Supplementary material for: Fluorescence‐Lifetime Imaging and Super‐Resolution Microscopies Shed Light on the Directed‐ and Self‐Assembly of Functional Porphyrins onto Carbon Nanotubes and Flat Surfaces
Source: Chemistry. 2017 May 19;23(41):9772–89. doi: 10.1002/chem.201605232 (PMC5724654; doi:10.1002/chem.201605232)
Supplement: Supplementary file 1 — Supplementary [file CHEM-23-9772-s001.pdf]

CHEMISTRY
A European Journal

Supporting Information

Fluorescence-Lifetime Imaging and Super-Resolution Microscopies Shed Light on the Directed- and Self-Assembly of Functional Porphyrins onto Carbon Nanotubes and Flat Surfaces

Boyang Mao+,[a, b] David G. Calatayud+,[a, c] Vincenzo Mirabello+,[a] Navaratnarajah Kuganathan,[d] Haobo Ge,[a] Robert M. J. Jacobs,[e] Ashley M. Shepherd,[e] José A. Ribeiro Martins,[f] Jorge Bernardino De La Serna,[g] Benjamin J. Hodges,[a] Stanley W. Botchway,[g] and Sofia I. Pascu*[a]

chem_201605232_sm_miscellaneous_information.pdf

Table of content

1.General details for materials characterization

2. Single crystal X-ray diffraction

3. Mass spectrometry

4. HPLC

5. NMR spectroscopy including Diffusion-Ordered NMR Spectroscopy

6. Tapping mode AFM (TMAFM) to identify the morphology information of the Zn(II)-porphyrin (1) aggregation on supports

7. Solid state investigation by TEM

8. Solid state investigation by SEM

9. Solid state investigation by AFM

10. FTIR Spectroscopic characterization

11. Raman Spectroscopic characterization

12. DFT calculations

13. UV-vis titrations and fitting of association binding constant.

14. Single-photon laser confocal fluorescence

15. Two-photon laser confocal fluorescence

16. STED Super Resolution Microscopy

17. The coronene challenge experiment

18. SWNTs-Porphyrin UV-vis titration

1. General details for materials characterization

Transmission electron microscopy (TEM) images were obtained with Gatan Dualvison digital camera on a JEOL 1200EXII transmission electron microscope coupled with Energy-dispersive X-ray spectroscopy (point resolution, 0.16 nm).

Raman spectroscopy was carried out on a Renishaw inVia Raman spectroscopy. The specimens were either in solid state or dispersed in pure water (MilliQ) or water: ethanol 1:1 mixture. During the measurement, the carbon nanomaterials samples were deposited on an aluminum plate substrate. The input wavelength was set at 514 nm. More than 10 times accumulations were generally applied in Raman spectroscopy measurements, and the beam was focused in at least three different positions across the specimen and these spectra were averaged to obtain batch-representative peaks and most reliable results.

Fluorescence spectroscopy measurement was carried out on a Perkin Elmer Luminescence spectrophotometer LS 55. The concentration of sample applied to fluorescence spectroscopy can be vary between 10^{-5}-10^{-7} M depend on the strength of the fluorescence. UV-vis spectroscopy was carried out by using a Perkin-Elmer Lambda 35 spectrometer.

The Atomic Force Microscopy measurements were carried out a Digital Instruments Multimode Atomic Force Microscope with IIIa controller. All AFM measurements were obtained under tapping mode and using Silicon Probes (Nascatec GmbH model NST-NCHFR). The AFM samples were deposited onto freshly cleared mica substrate by spin coating (Laurell Technologies WS-400, 3000rpm).

Fourier transform Infrared spectra were obtained by using a Perkin-Elmer 1000 FT-IR spectrometer.

XP spectra of calcined samples were collected using a VG Escalab II spectrometer using aluminum Kα radiation (1486.6 eV) and a hemispherical analyzer for detection of electrons. The Pass Energy was set at 50 eV for survey scan of the sample, and 20 eV for the more intense scans of specific areas. The resulting spectra were analyzed using CasaXPS peak fitting software, and sample charging corrected setting the C 1s (C-C) signal at 284.5 eV. Solution multinuclear

NMR spectra were recorded on a Bruker Advance 500MHz spectrometer. 1H and 13C chemical shifts are referenced to tetramethylsilane. ^{1}H and ^{13}C chemical shift assignments

were made by using standard COSY (cosygpqf), HMQC (hmqcgpqf) and DOSY (ledbpgp2) pulse sequences outlined within the Bruker library. Diffusion-oriented experiments were processed by using Topspin3.5 and then fitted on BRUKER Dynamic Center 2.3 software.

2. Single crystal X-ray diffraction

Table S1. Crystal and structure refinement data for Zn(II)-porphyrin dimer

Empirical formula	$C_{124} H_{156} N_8 S_4 Zn_2$
Formula weight	2017.59
Crystal system	Orthorhombic
Space group	Pbca
a/Å	17.629(5)
b/Å	17.624(5)
c/Å	34.458(5)
α/º	90
β/º	90
γ/º	90
Volume/ $Å^3$	10705.9(46)
Z	4
Density (calculated)/ Mg/m^3	1.252
Absorption coefficient mm^{-1}	0.535
F(000)	4320
GOF	1.0912
Reflections collected	21909
Independent reflections	6174 [R(int) = 0.04]
Final R indices [I>2sigma(I)]	R1 = 0.0611, wR2 = 0.0682
Largest diff. peak and hole/ $e.Å^{-3}$	0.79 and -0.65

Table S2. Selected bond lengths [Å] and angles [°] for Zn(II)-porphyrin dimer.

Zn(1)-N(1)	2.054(6)	Zn(1)-N(2)	2.032(6)
Zn(1)-N(3)	2.046(6)	Zn(1)-N(4)	2.043(6)
S(1)-S(2)#1	2.029(4)	S(1)-C(31)	1.817(9)
S(2)-C(32)	1.814(11)	N(1)-C(1)	1.36(1)
N(1)-C(4)	1.391(9)	N(2)-C(6)	1.383(9)
N(2)-C(9)	1.362(9)	N(3)-C(11)	1.371(9)
N(3)-C(14)	1.375(9)	N(4)-C(16)	1.39(1)
N(4)-C(19)	1.368(9)	C(1)-C(2)	1.452(11)
C(1)-C(20)	1.395(11)	C(2)-C(3)	1.348(11)
C(2)-C(39)	1.523(11)	C(3)-C(4)	1.46(1)
C(3)-C(21)	1.513(12)	C(4)-C(5)	1.406(11)
C(5)-C(6)	1.41(1)	C(5)-C(25)	1.51(1)
C(6)-C(7)	1.463(11)	C(7)-C(8)	1.35(1)
C(7)-C(22)	1.526(11)	C(8)-C(9)	1.44(1)
C(8)-C(45)	1.51(1)	C(9)-C(10)	1.41(1)
C(10)-C(11)	1.39(1)	C(11)-C(12)	1.44(1)
C(12)-C(13)	1.38(1)	C(12)-C(51)	1.52(1)
C(13)-C(14)	1.44(1)	C(13)-C(23)	1.51(1)
C(14)-C(15)	1.423(11)	C(15)-C(16)	1.410(11)
C(15)-C(35)	1.492(11)	C(16)-C(17)	1.483(11)
C(17)-C(18)	1.355(11)	C(17)-C(24)	1.515(12)
C(18)-C(19)	1.438(11)	C(18)-C(57)	1.520(11)
C(19)-C(20)	1.381(11)		
N(1)-Zn(1)-N(2)	87.3(2)	N(1)-Zn(1)-N(3)	176.6(2)
N(2)-Zn(1)-N(3)	93.0(2)	N(1)-Zn(1)-N(4)	91.6(2)

N(2)-Zn(1)-N(4)	174.4(2)	N(3)-Zn(1)-N(4)	87.7(2)
S(2)-S(1)-C(31)	105.4(3)	S(1)-S(2)#1-C(32)#1	103.3(4)
Zn(1)-N(1)-C(1)	124.6(5)	Zn(1)-N(1)-C(4)	129.3(5)
C(1)-N(1)-C(4)	106.0(6)	Zn(1)-N(2)-C(6)	129.7(5)
Zn(1)-N(2)-C(9)	124.3(4)	C(6)-N(2)-C(9)	105.9(6)
Zn(1)-N(3)-C(11)	123.9(5)	Zn(1)-N(3)-C(14)	129.5(5)
C(11)-N(3)-C(14)	106.1(6)	Zn(1)-N(4)-C(16)	128.9(5)
Zn(1)-N(4)-C(19)	124.7(5)	C(16)-N(4)-C(19)	106.4(6)
N(1)-C(1)-C(2)	111.1(7)	N(1)-C(1)-C(20)	124.8(7)
C(2)-C(1)-C(20)	124.1(7)	C(1)-C(2)-C(3)	106.6(7)
C(1)-C(2)-C(39)	124.8(7)	C(3)-C(2)-C(39)	128.6(7)
C(2)-C(3)-C(4)	107.3(7)	C(2)-C(3)-C(21)	122.8(7)
C(4)-C(3)-C(21)	129.9(7)	N(1)-C(4)-C(3)	109.1(7)
N(1)-C(4)-C(5)	123.6(7)	C(3)-C(4)-C(5)	127.3(7)
C(4)-C(5)-C(6)	125.5(7)	C(4)-C(5)-C(25)	116.4(7)
C(6)-C(5)-C(25)	118.0(7)	N(2)-C(6)-C(5)	124.1(7)
N(2)-C(6)-C(7)	109.1(6)	C(5)-C(6)-C(7)	126.8(7)
C(6)-C(7)-C(8)	107.0(7)	C(6)-C(7)-C(22)	130.2(7)
C(8)-C(7)-C(22)	122.8(7)	C(7)-C(8)-C(9)	106.6(7)
C(7)-C(8)-C(45)	128.4(7)	C(9)-C(8)-C(45)	124.8(7)
N(2)-C(9)-C(8)	111.4(6)	N(2)-C(9)-C(10)	124.7(6)
C(8)-C(9)-C(10)	123.5(7)	C(9)-C(10)-C(11)	128.5(7)
N(3)-C(11)-C(10)	124.8(7)	N(3)-C(11)-C(12)	110.6(6)
C(10)-C(11)-C(12)	124.3(7)	C(11)-C(12)-C(13)	106.5(6)
C(11)-C(12)-C(51)	125.2(7)	C(13)-C(12)-C(51)	128.2(7)
C(12)-C(13)-C(14)	106.3(6)	C(12)-C(13)-C(23)	121.1(7)

C(14)-C(13)-C(23)	132.3(7)	N(3)-C(14)-C(13)	110.4(7)
N(3)-C(14)-C(15)	123.6(7)	C(13)-C(14)-C(15)	125.9(7)
C(14)-C(15)-C(16)	124.9(7)	C(14)-C(15)-C(35)	118.8(7)
C(16)-C(15)-C(35)	116.3(7)	N(4)-C(16)-C(15)	124.5(7)
N(4)-C(16)-C(17)	109.1(7)	C(15)-C(16)-C(17)	126.3(7)
C(16)-C(17)-C(18)	105.9(7)	C(16)-C(17)-C(24)	131.1(8)
C(18)-C(17)-C(24)	122.9(8)	C(17)-C(18)-C(19)	108.0(7)
C(17)-C(18)-C(57)	127.5(7)	C(19)-C(18)-C(57)	124.5(8)
N(4)-C(19)-C(18)	110.6(7)	N(4)-C(19)-C(20)	124.8(7)
C(18)-C(19)-C(20)	124.5(7)	C(1)-C(20)-C(19)	128.7(7)

Symmetry transformations used to generate equivalent atoms:
#1 -x+2,-y-1,-z+1

3. Mass spectrometry

Mass spectrometry data were obtained on a Bruker Daltonics electrospray ionisation – time-of-flight (ESI-TOF) mass spectrometry at University of Bath. All mass spectrometry samples were prepared by dissolving in methanol, acetonitrile or distilled water. For porphyrin molecule sample characterisation, a few drops of formic acid were introduced to the solution to obtain better ionisation. The concentration of measured samples should be in a range of 1 µg/mL to 10 µg/mL. Positive loop injection and negative loop injection are the two method used in the paper. Mass spectrometry data can be further analysed by Bruker Daltonics Data Analysis 4.0 software.

Free base porphyrin:

Mass spectrum ESI-MS calculated for $C_{66}H_{87}N_4O_2S_2^+$ $[M+H]^+$ 1030.62, found 1031.63.

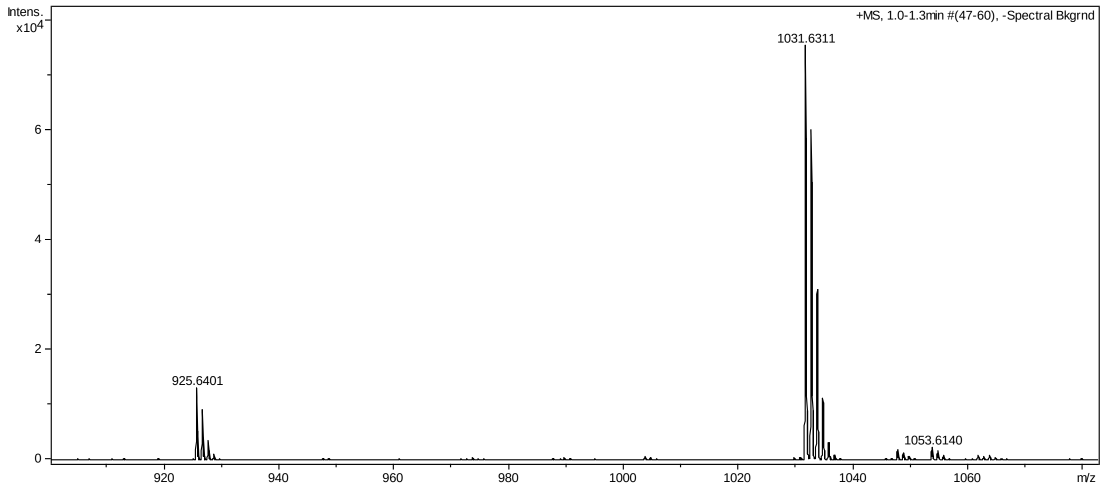

Figure S1. Mass spectrum of free base porphyrin

Zn(II)-porphyrin (protected, Compound 1)

Mass spectrum ESI-MS calculated for $C_{66}H_{83}N_4O_2S_2Zn^-$ $[M-H]^-$ 1091.53, found 1091.59.

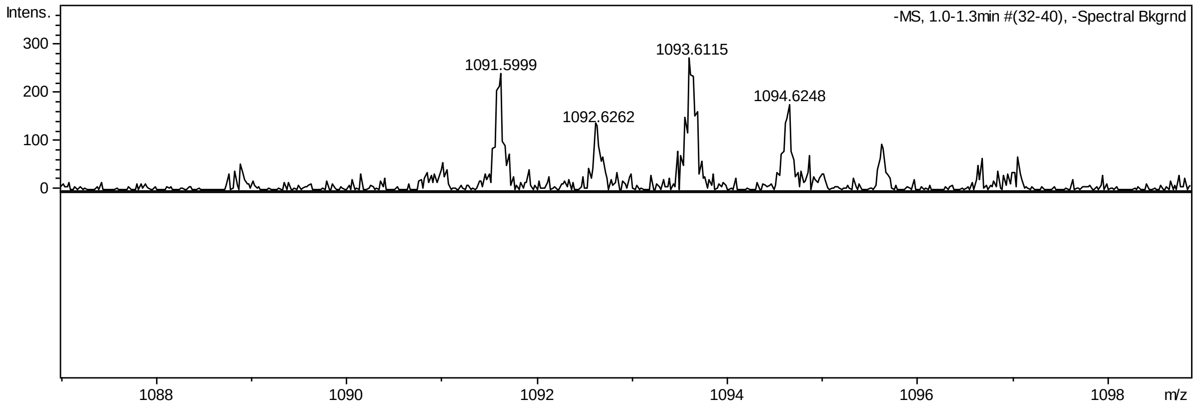

Figure S2. Mass spectrum of Zn(II)-porphyrin, compound **1**

De-protected Zn(II)-porphyrin (Compound 3):

Mass spectrum ESI-MS calculated for $C_{62}H_{80}N_4S_2ZnNa^+$ $[M+Na]^+$ 1031.51, found 1031.62.

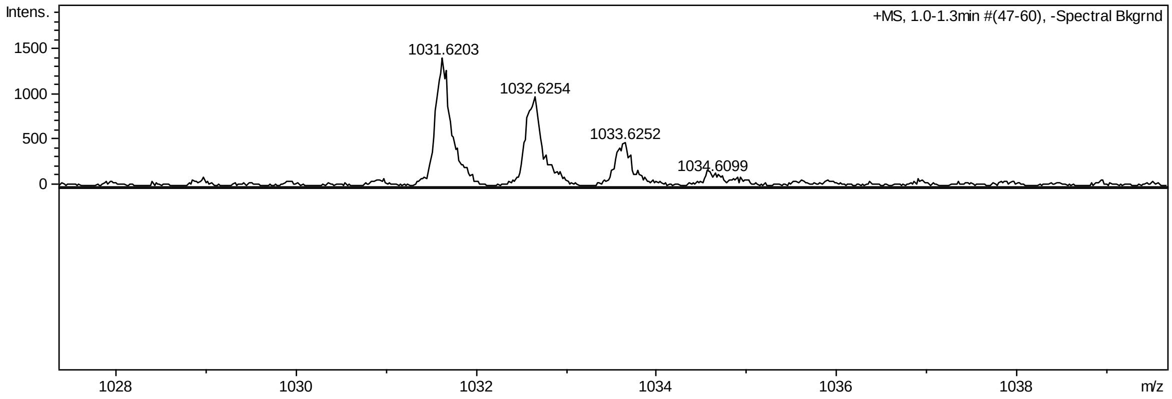

Figure S3. Mass spectrum of deprotect Zn(II)-porphyrin, compound **3**

4. HPLC

To further investigate the synthesis of Zn(II)-porphyrin and free base porphyrin, HPLC characterisations of the resulting materials were carried out. The measured results are shown in Figure S42. The peak appeared in between 2-2.5 min were due to the injection of samples in DMSO, but that at 2.5-3.5 min be arguable into ligand for $M(OH)_n$ species (n=2, M=Zn(II)). Free base porphyrin did not show the peak in the 2.5-3.5 min, and presented a main peak at 14.697 min, which means the free base porphyrin was majorly pure. For Zn(II)-porphyrin, similar measured result can be observed: there was a main absorption peak at 14.707 min.

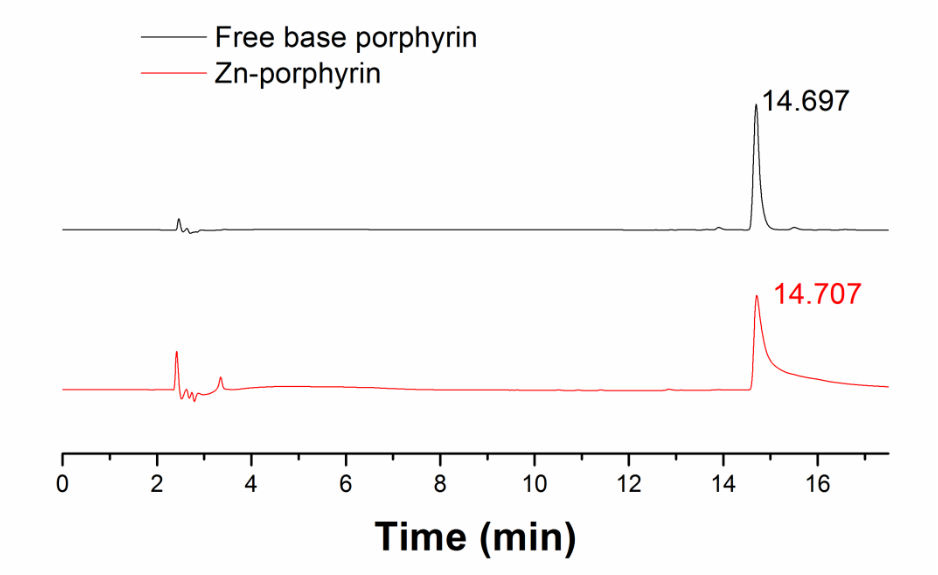

Figure S4. HPLC of Zn(II)-poprhyin and free base porphyrin

5. NMR

Solution multinuclear NMR spectra were recorded on a Bruker Avance 500MHz spectrometer. ^{1}H and ^{13}C chemical shifts are referenced to tetramethylsilane. ^{1}H and ^{13}C chemical shift assignments were made by using standard COSY (cosygpqf), HMQC (hmqcgpqf) and DOSY (ledbpgp2) pulse sequences outlined within the Bruker library. Diffusion-oriented experiments were processed by using Topspin3.5 and then fitted on BRUKER Dynamic Center 2.3 softwave.

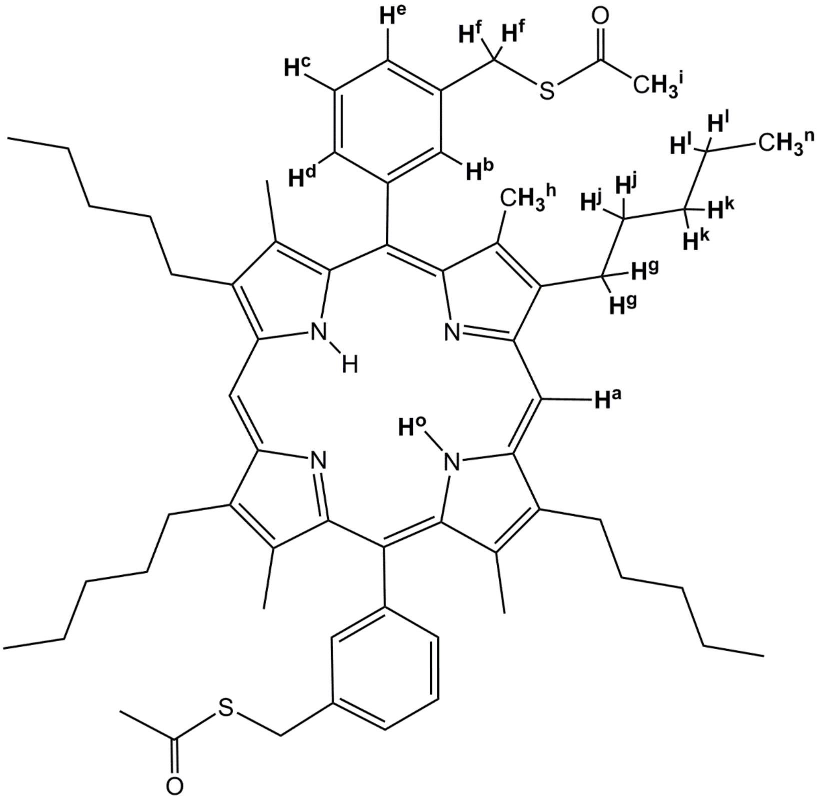

Scheme S1. For clarity, magnetically equivalent nuclei within the same NMR spin system were only labeled once.

^{1}H NMR (500 MHz, 298 K, $CDCl_3$) δ 10.26 (s, H^a, 2H), 8.05 (bs, H^b, 2H), 8.00 – 7.93 (m, H^c, 2H), 7.78 – 7.65 (m, H^d, 2H), 4.43 (s, H^f, 4H), 4.01 (t, H^g, J_{gj} = 8.0 Hz, 8H), 2.50 (bs, H^h, 12H), 2.42 (s, H^i, 6H), 2.22 (m or pquint, J_{jk} = 8.2 Hz, H^j, 8H), 1.76 (m or pquint, J_{kl} = 7.4, H^k, Hz, 8H), 1.51 – 1.41 (m, H^l, 8H), 1.43 – 1.34 (m, H^m, 8H), 0.94 (m or bt, J_{mn} = 7.0 Hz, , H^n ,12H), -2.39 (s, H^o, 2H) ppm. **^{13}C NMR** (126 MHz, 298 K, $CDCl_3$) δ 195.0 (-S***C***OCH_3^{i}), 145.0, 143.39, 142.6, 141.5 (Pyrrole ***C***), 133.4 (***C***H^b), 132.0 (***C***H^c), 128.7 (***C***H^d), 127.9 (***C***H^e), 97.0 (***C***H^a), 33.8 (***C***H^f), 33.3 (***C***H^i), 32.0 (***C***H^l), 30.4 (***C***H^g), 30.0 (***C***H^k), , 22.8 (***C***H^m), 14.8 (***C***H^h), 14.2 (***C***H^n) ppm.

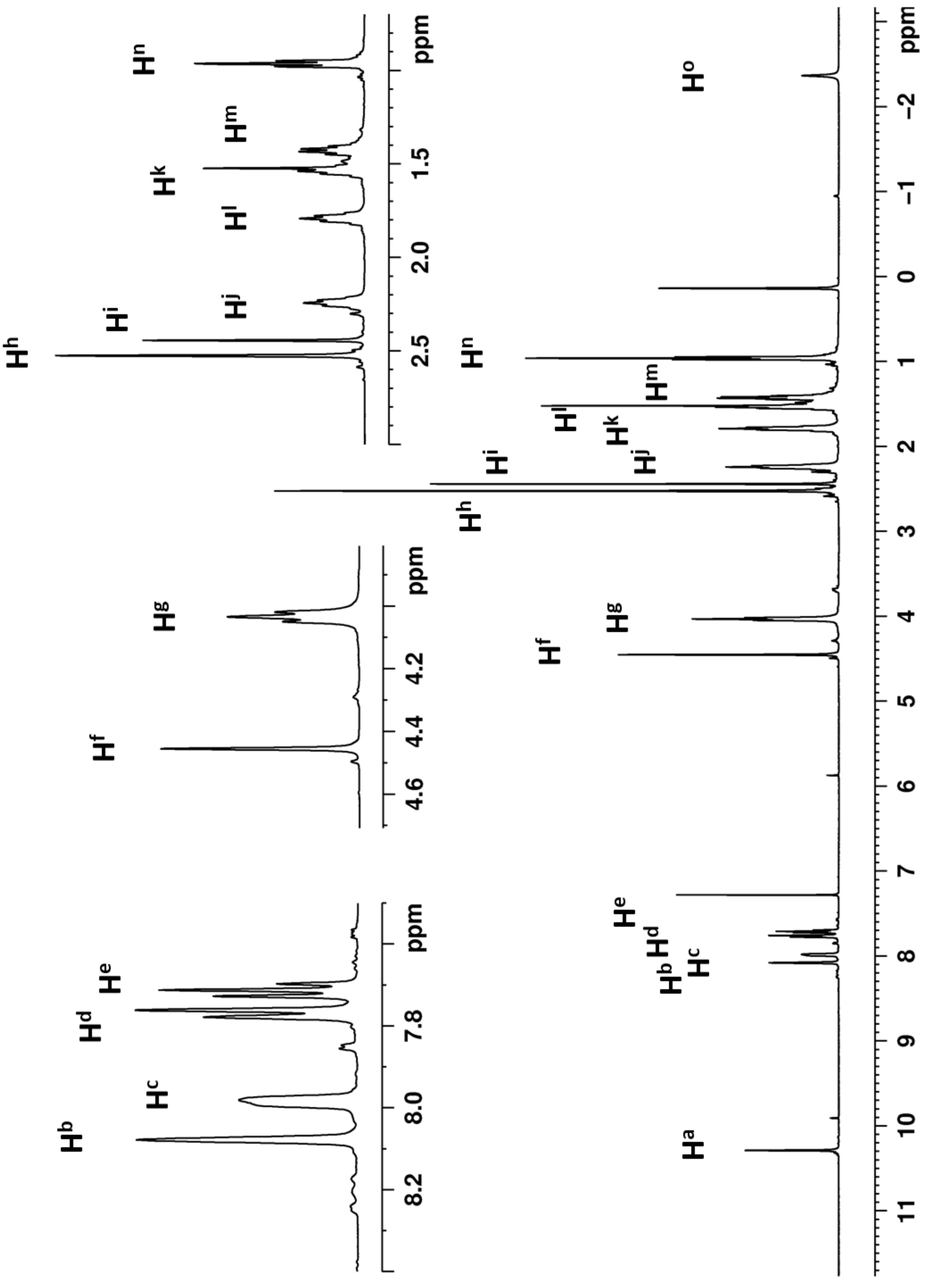

Figure S5. ^{1}H NMR spectrum (500 MHz, 298 K, $CDCl_3$) of free base porphyrin precursor of (**1**).

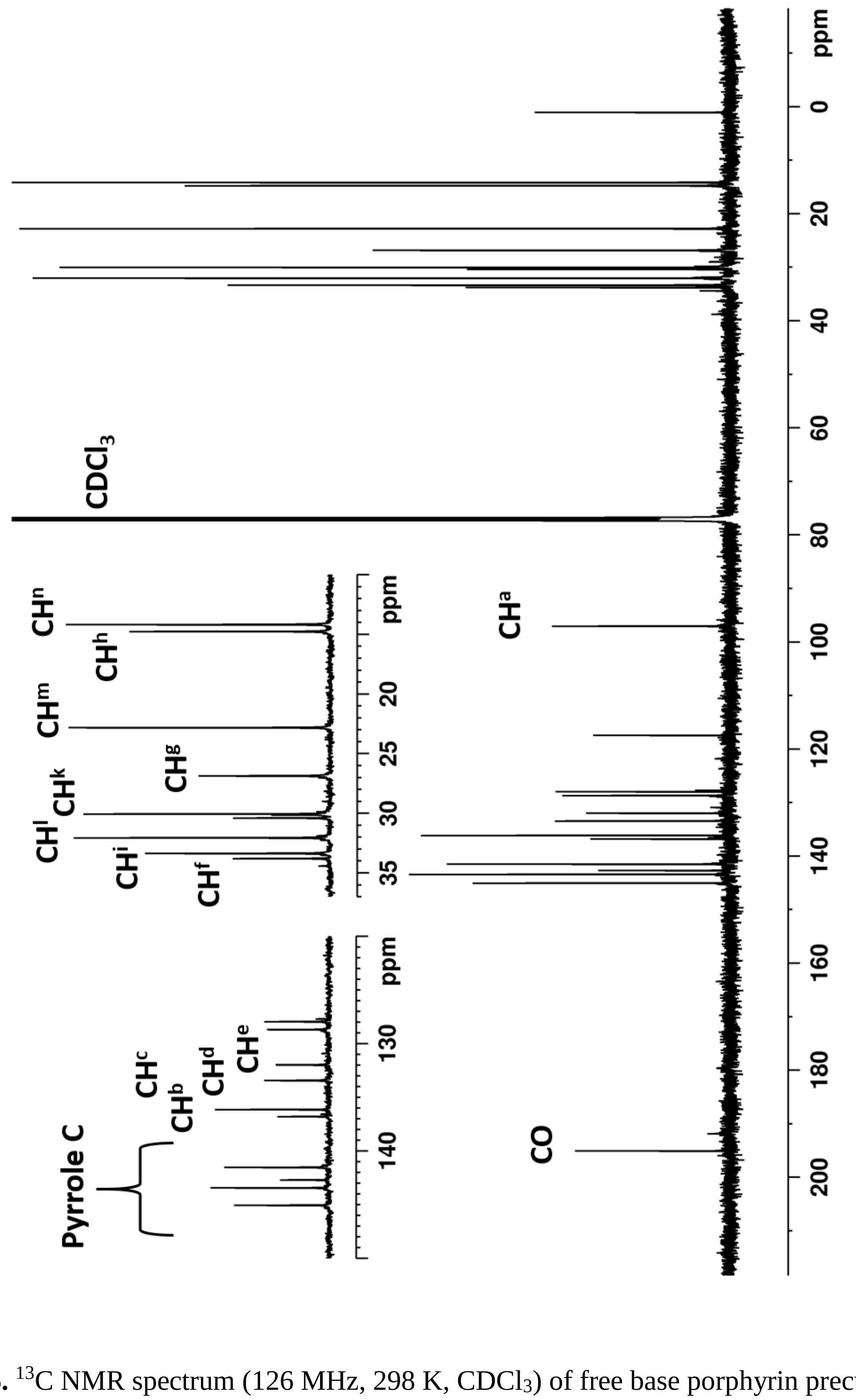

Figure S6. ^{13}C NMR spectrum (126 MHz, 298 K, $CDCl_3$) of free base porphyrin precursor of (**1**).

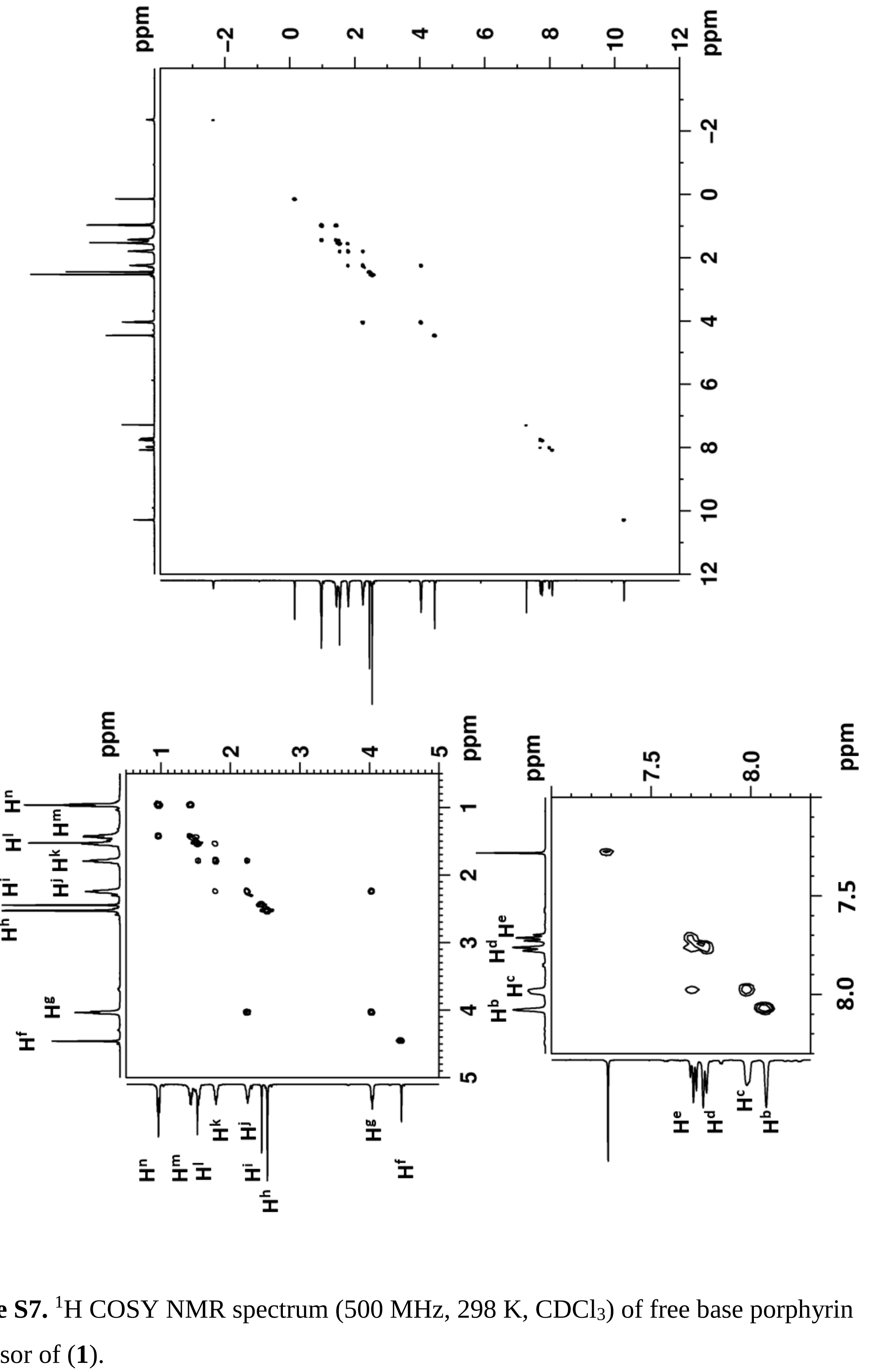

Figure S7. ^{1}H COSY NMR spectrum (500 MHz, 298 K, $CDCl_3$) of free base porphyrin precursor of (**1**).

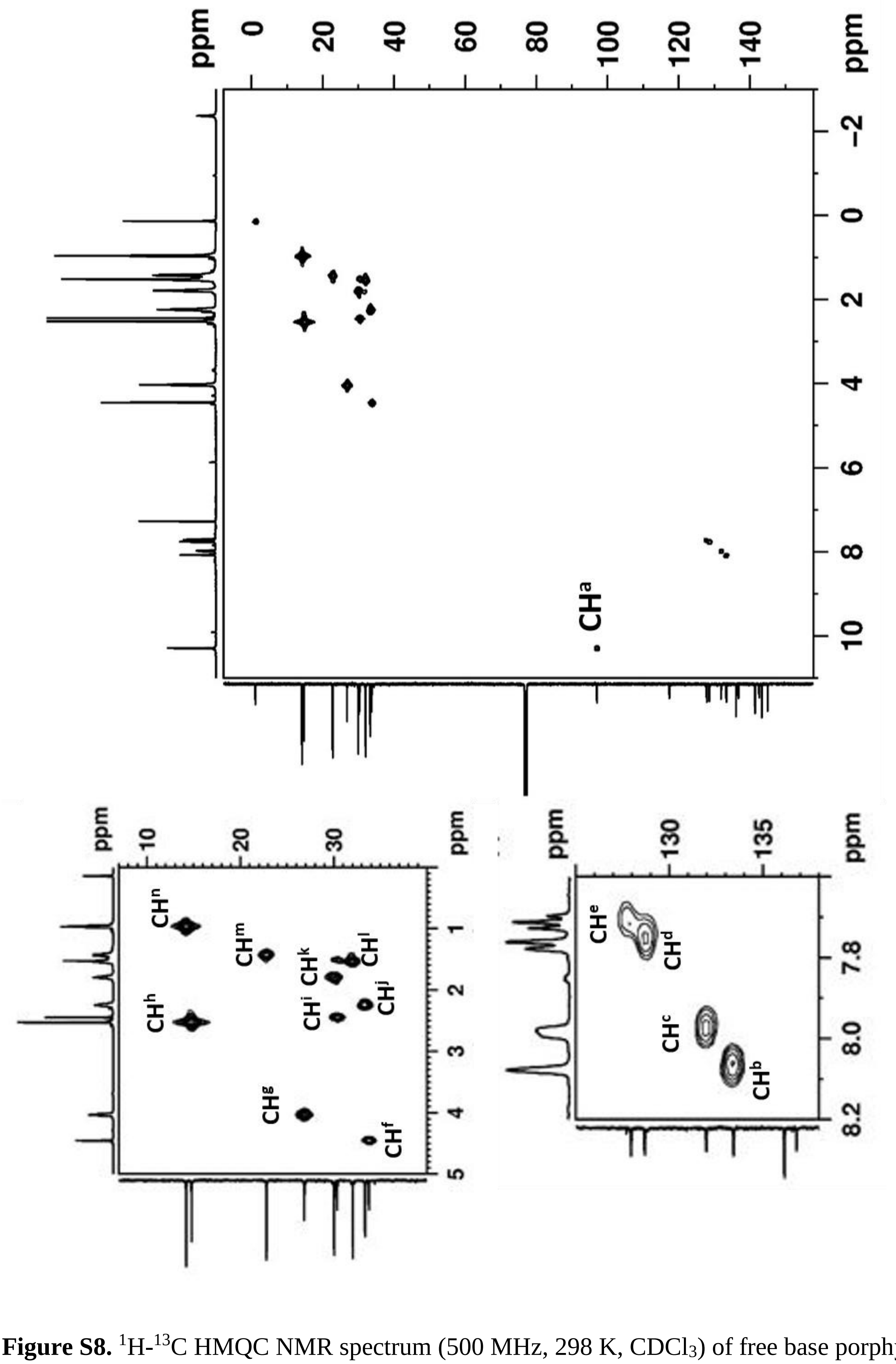

Figure S8. ^{1}H-^{13}C HMQC NMR spectrum (500 MHz, 298 K, $CDCl_3$) of free base porphyrin precursor of (**1**).

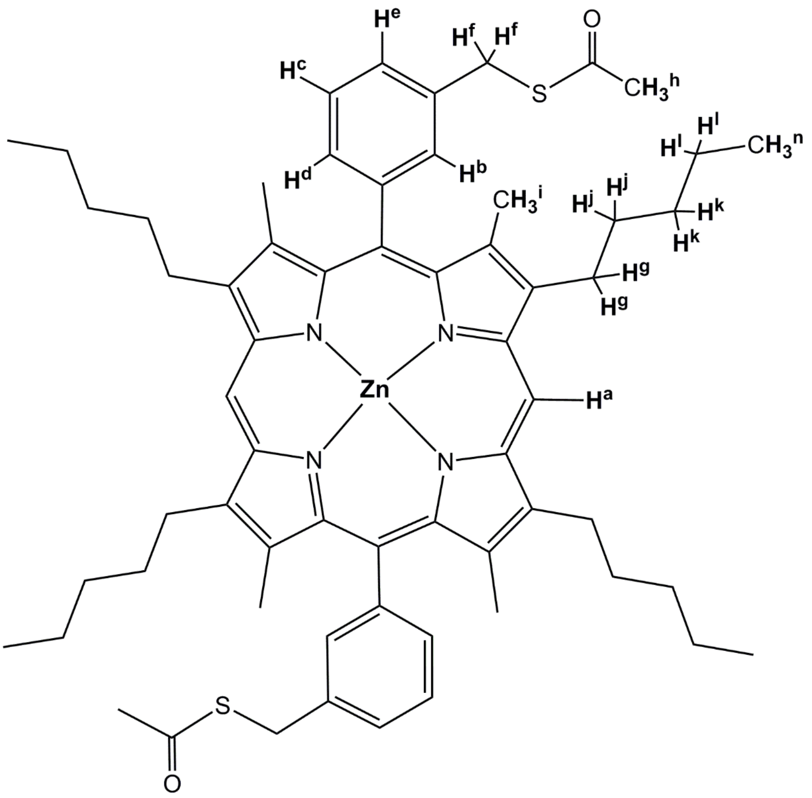

Scheme S2. For clarity, magnetically equivalent nuclei within the same NMR spin system were labeled once.

^{1}H NMR (500 MHz, 298 K, $CDCl_3$) δ 10.04 (s, H^a, 2H), 7.98 (bs, H^b, 2H), 8.00 – 7.84 (m, H^c, 2H), 7.78 – 7.68 (m, H^d, 4H), 4.41 (s, H^f, 4H), 3.92 b(t, H^g, J_{gj} = 7.7 Hz, 8H), 2.38 (s, H^h, 6H), 2.42 (s, H^i, 12H), 2.11 (m or pquint, J_{jk} = 7.3 Hz, H^j, 8H), 1.71 (m or pquint, J_{kl} = 7.7 Hz, H^k, 8H), 1.52 – 1.43 (m, H^l, 8H), 1.40 – 1.30 (m, H^m, 8H), 0.88 (t, J_{mn} = 7.4 Hz, H^n ,12H) ppm.

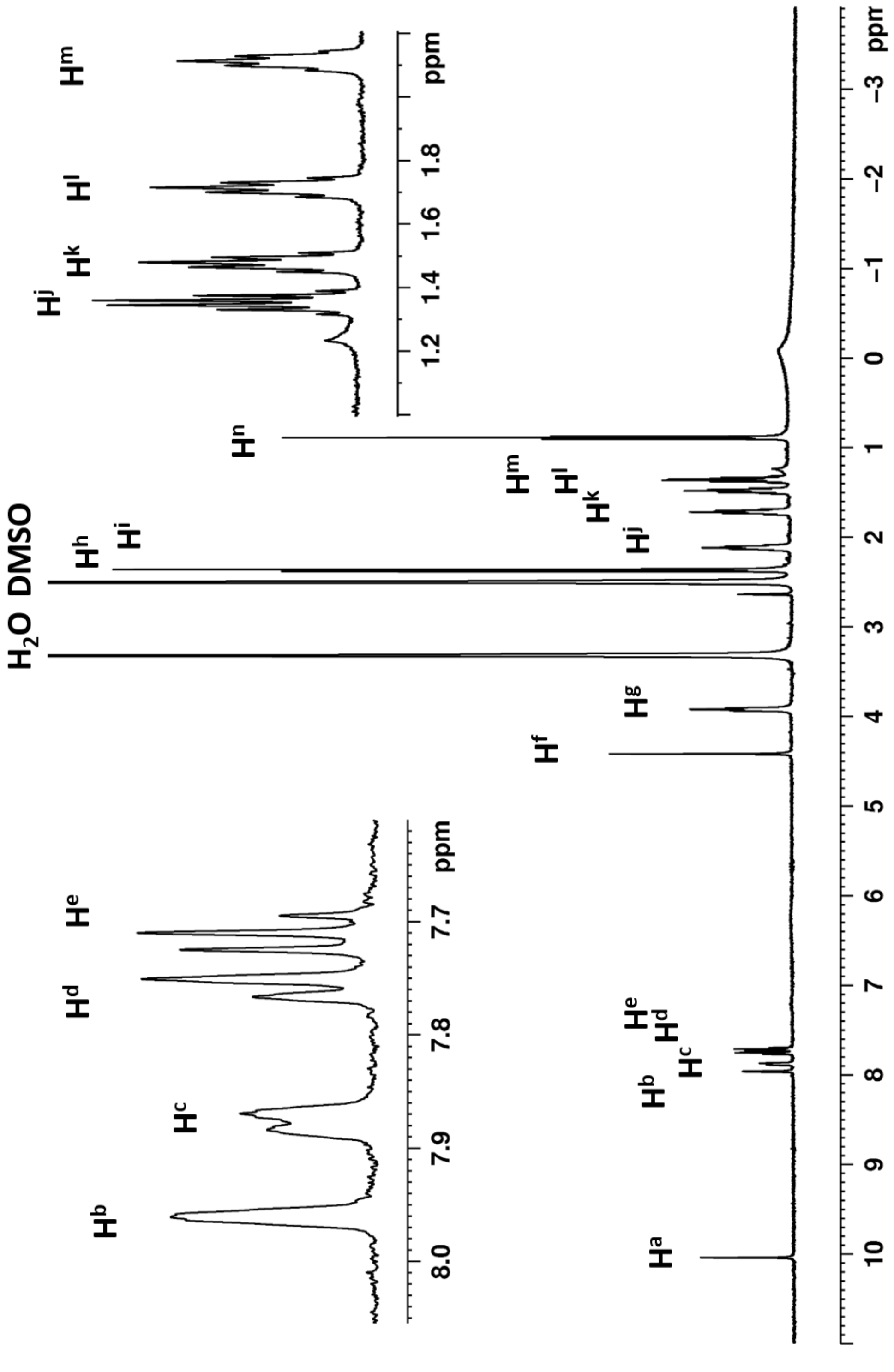

Figure S9. ^{1}H NMR spectrum (500 MHz, 298 K, $CDCl_3$) of (**1**).

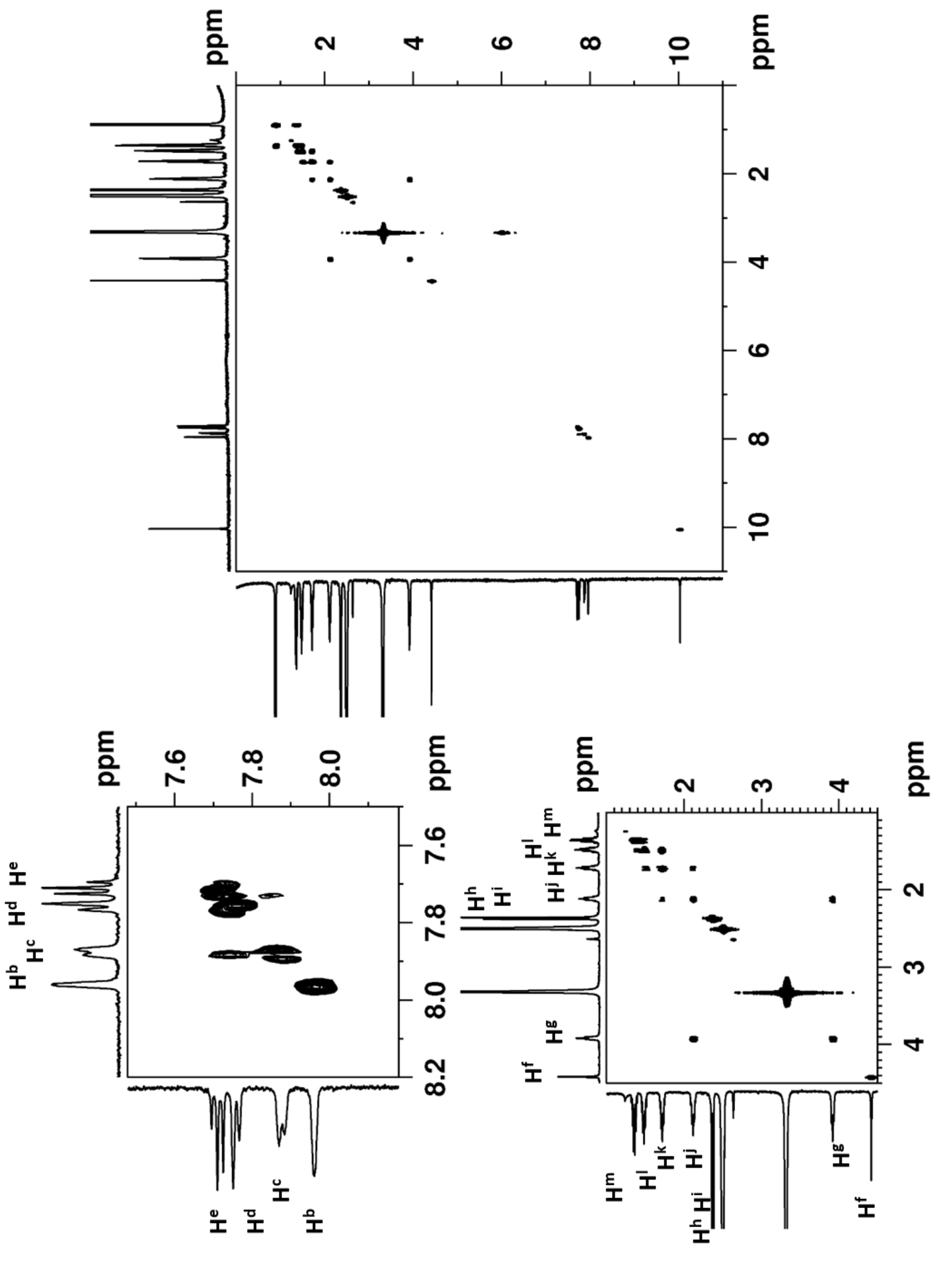

Figure S10. ^{1}H COSY NMR spectrum (500 MHz, 298 K, $CDCl_3$) of (**1**).

Diffusion-Ordered NMR Spectroscopy

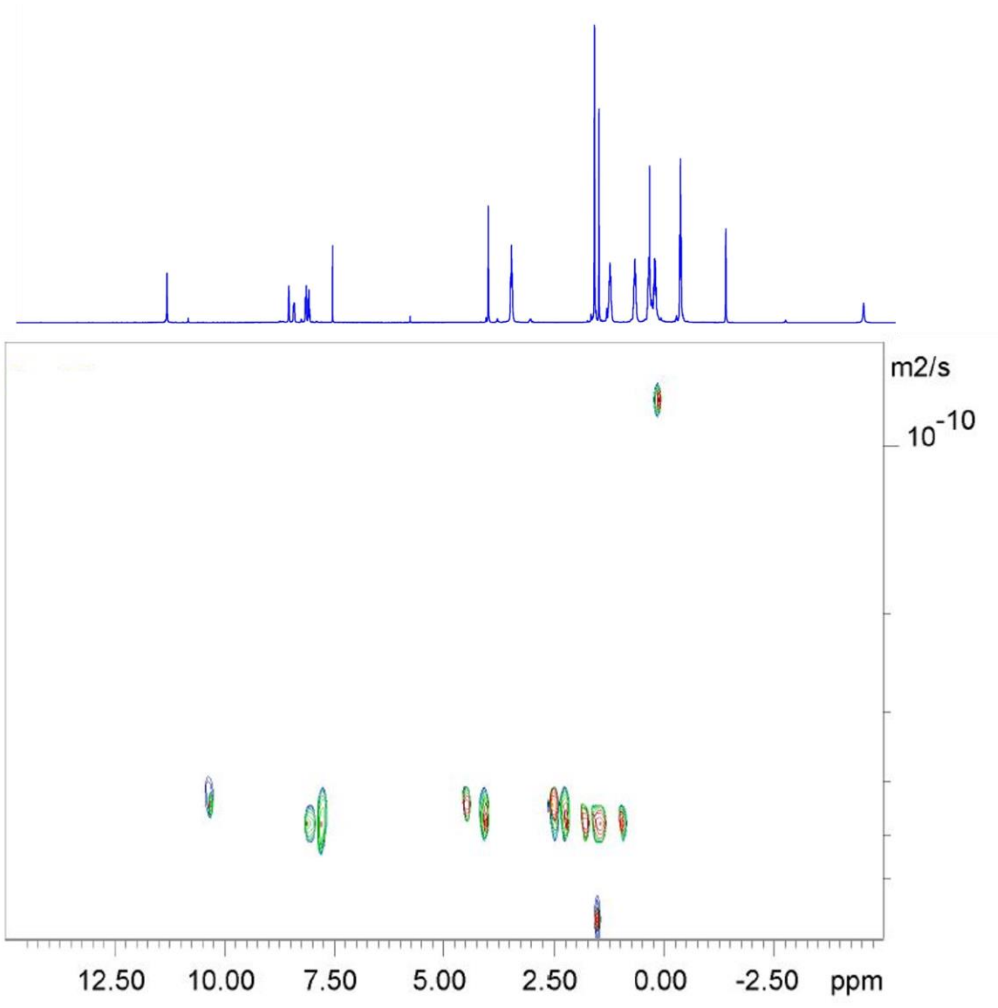

Figure S11. ^{1}H DOSY NMR spectrum (500 MHz, 298 K, $CDCl_3$) of free base porphyrin precursor of (**1**). Diffusion coefficient = 4.57x 10^{-10} m^2 s^{-1} suggestion lack of meaningful aggregation levels in solutions at the ca 5 mM conc. needed to record a spectrum.

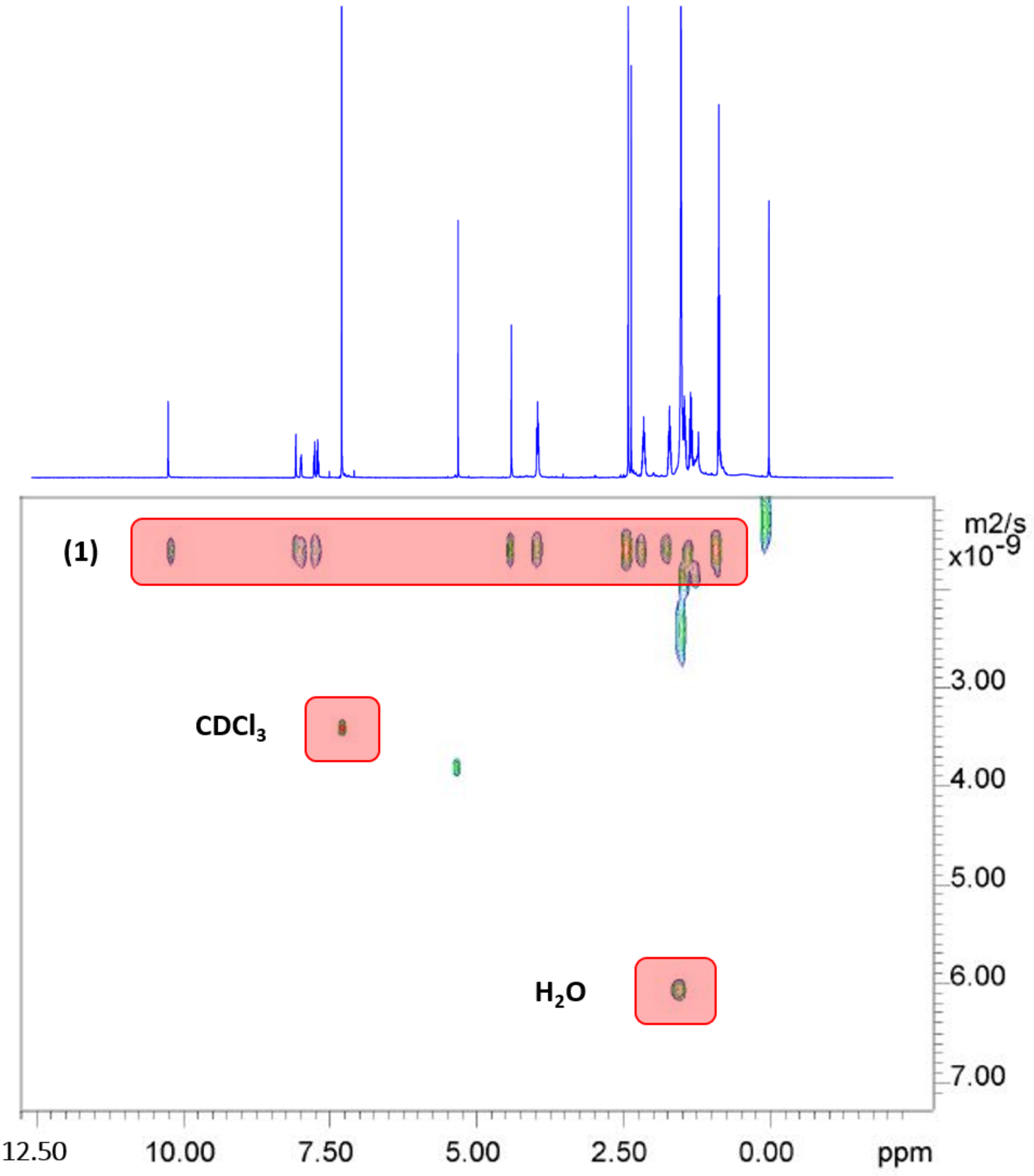

Figure S12. ^{1}H DOSY NMR spectrum (500 MHz, 298 K, $CDCl_3$) of (**1**). Diffusion coefficient = 1.62x 10^{-9} m^2 s^{-1} which seems to indicate a limited ability of this Zn(II) prophyrin to aggregate in solution even at the ca 5 mM conc. needed to record a spectrum.

6. Tapping mode AFM (TMAFM) to identify the morphology information of the Zn(II)-porphyrin (1) aggregation on supports

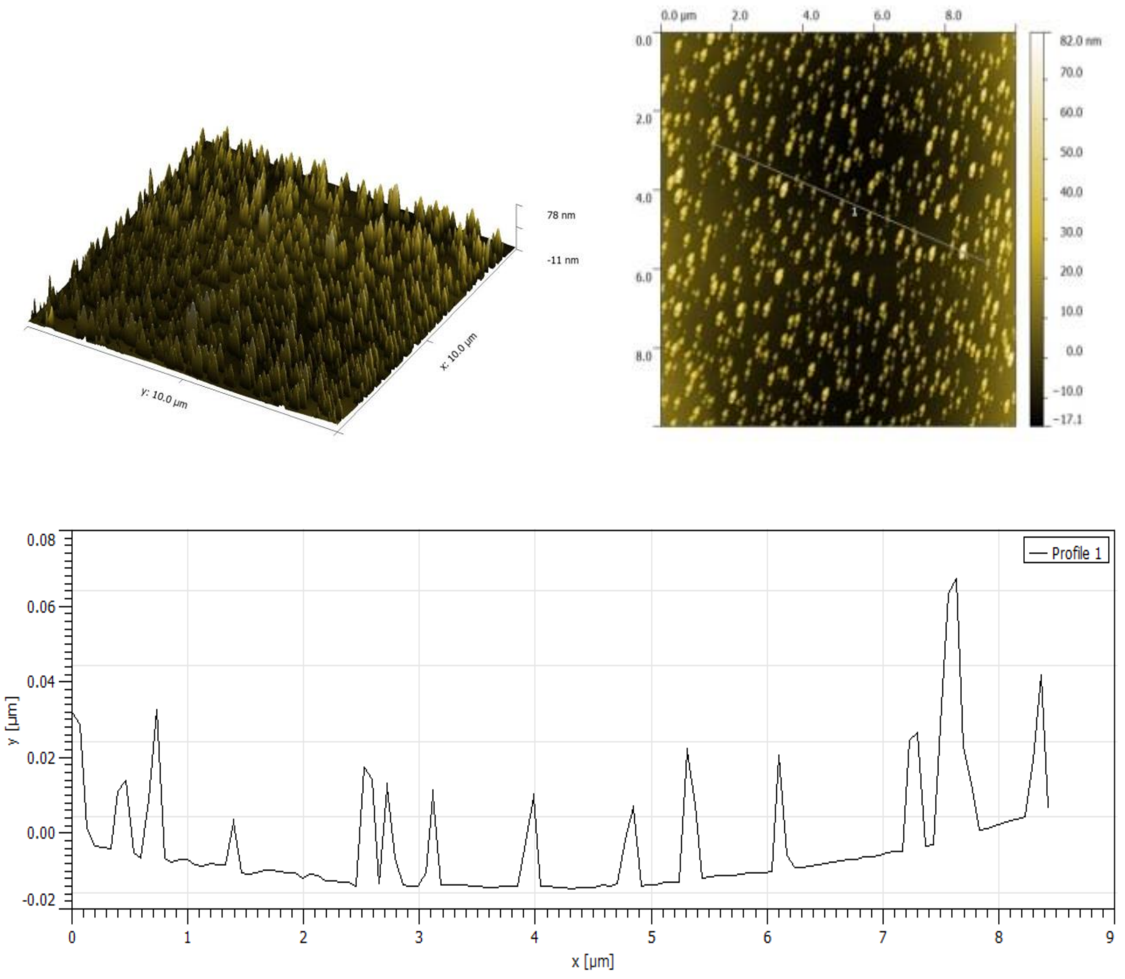

Figure S13: (a) 3D TMAFM image of Zn(II)-porphyrin (compound **1**) self-assembly on Mica after spin coating, The TMAFM measurement of Zn(II)-porphyrin sample was prepared by using a spin coating at 3000 rpm and coating the Zn(II)-porphyrin solution 18.3 µM onto a 2 cm^2 Mica substrate. **(b)** Tapping mode AFM image of Zn(II)-porphyrin self-assembly crystal; **(c)** Profile analysis showing the heights of line area.

7. Solid state investigation by TEM

Characterisation of the Covalently linked Zn(II)-porphyrin and SWNTs complex.

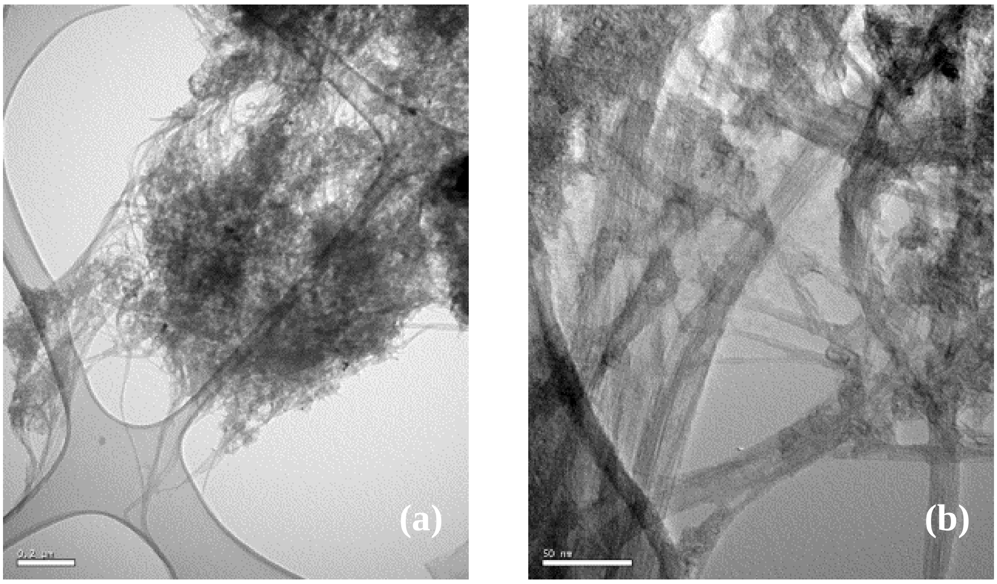

Figure S14: TEM image of **(a)** non-covalently linked SWNTs@Zn(II)-porphyrin complex (**2**), the scale bar is 200nm; **(b)** non-covalently linked SWNTs@Zn(II)-porphyrin complex (**2**), the scale bar is 50nm.

Transmission electron microscopy (TEM) provided direct morphology information of SWNTs surface area at the level of individual SWNTs or their thin bundles. The figure S10 above ((a) and (b)) show the surface morphology information of non-covalently linked compound **1**@SWNTs complex. From TEM images, there were strong contrast areas contributed by heavy element Zinc within the compound **1**, which were obviously visible compared with the lighter contrast regions (due to the lower scattering elements present, i.e. hydrogen, nitrogen and carbon from the SWNTs). Figure S10(b) shows more detailed surface morphology information that after complexation with compound **1**, the relatively clear and smooth SWNTs surface became rugged and there were some aggregations formed on it. Perceptibly, the side-wall of SWNT was significantly 'roughened' by the coverage of another material, which indicated the presence of compound **1** that coated on the surface of SWNT.[1],[2],[3],[4] The length of the SWNTs was not altered and the intact SWNTs structure indicated the controlled time sonication didn't introduce any surface defect. The TEM images can also indicate that the SNWTs were better dispersed in ethanol with a few bundles persist.

Additional TEM images of non-covalently linked SWNTs@Zn(II)-porphyrin are presented in main text.

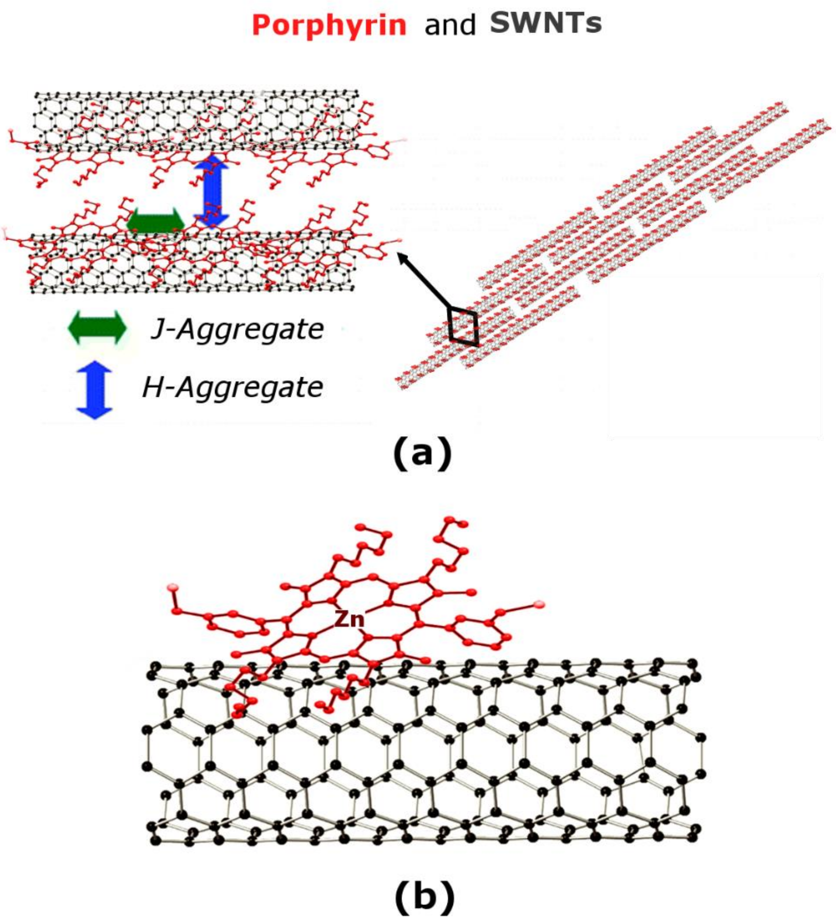

Figure S15: (a) Illustration of supramolecular assembly between porphyrins and single-walled carbon nanotubes in agreement to that emerging from Taku Hasobe's research;[5] **(b)** Schematic diagram showing the attachment of a non-covalently linked porphyrin **1** within the complex.

The compound **1** may contribute to the formation some complex bundles, as seen by TEM. Taku Hasobe's research_ENREF_32,[5] suggests that in this, and related, porphyrin@SWNTs systems, the porphyrin molecules can be used as a molecular glue by virtue of their π-π stacking on the surface of SWNTs to combine two functionalised SWNTs together and form a larger self-assembled structure. The work by Taku Hasobe indicated that the attaching angle of porphyrin and SWNTs was 0 degrees. This indicates that the combination of porphyrin and SWNTs occurs via a face-face orientation and the two systems in the donor-acceptor nano-complex are very closely held together, and may well apply to the systems studied herein too.

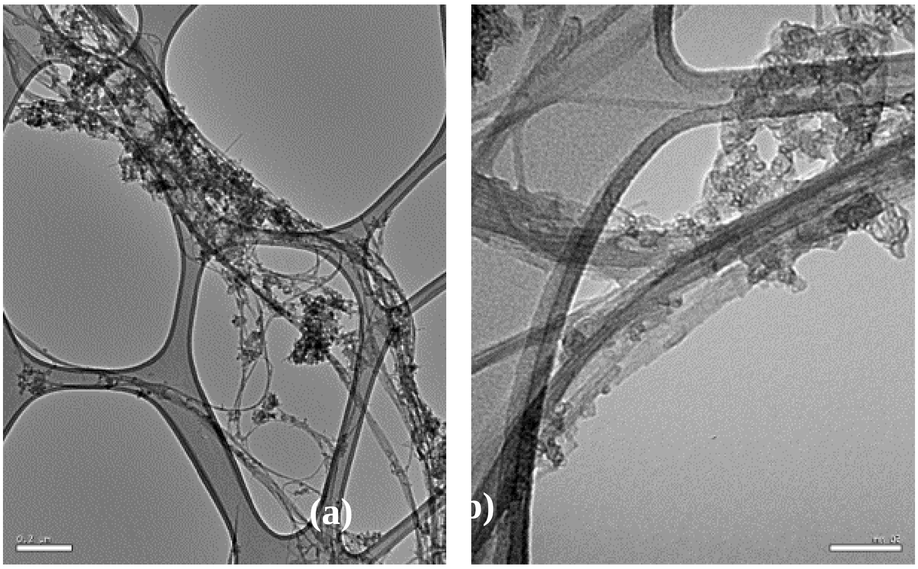

Figure S16: TEM image of (a) covalently linked SWNTs@Zn(II)-porphyrin complex (**6**), the scale bar is 200 nm; (b) covalently linked SWNTs@Zn(II)-porphyrin complex, the scale bar is 50 nm.

8. Solid state investigation by SEM

SEM measurement of SWNTs and its complex can present more surface information in a large dimension. The SEM measurements were carried out by dropping dispersed complex solution (concentration 18.3 μM), which was then treated with sonication and centrifugations, onto a HPGO SEM substrate.

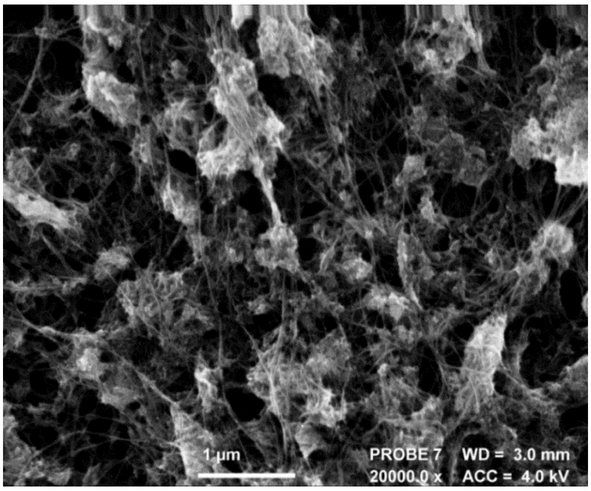

Figure S17: SEM image of non-covalently linked SWNT@Zn(II)-porphyrin complex (**2**), scale bar is 1000 nm.

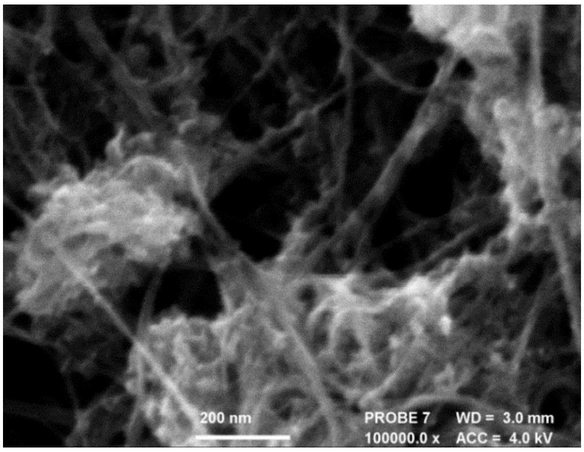

Figure S18: SEM image of non-covalently linked SWNT@Zn(II)-porphyrin complex (**2**), scale bar is 200nm.

The entire size of the network observed on HOPG of the compound **1**@SWNTs complex was around 200 nm. The zoomed SEM image gave some evidences in support of the the hypothesis proposed that the porphyrin could contribute to the linkage of two SWNTs together. It can be seen from the SEM images in Figure S17 and S18, which correspond to the non-covalently linked compound **1**@SWNTs complex that surface morphology information can be derived, specifically that the coating with compound **1** was uniform and covered the entire aromatic surface of SWNTs. After the complexation, there appeared aggregates onto the SWNTs surface.

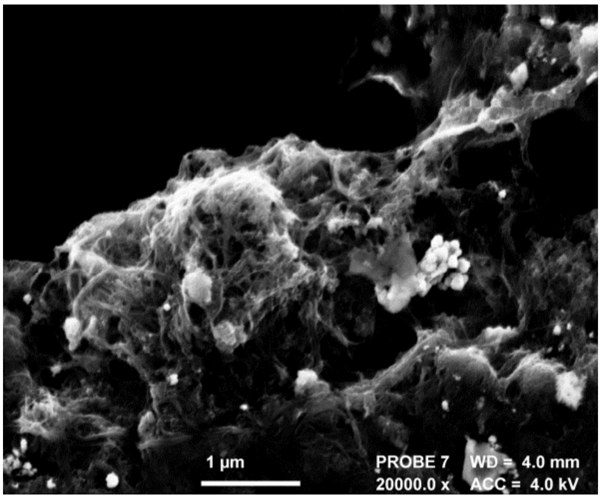

Figure S19. SEM image of covalently linked compound **1**@SWNTs complex, scale bar is 1000nm.

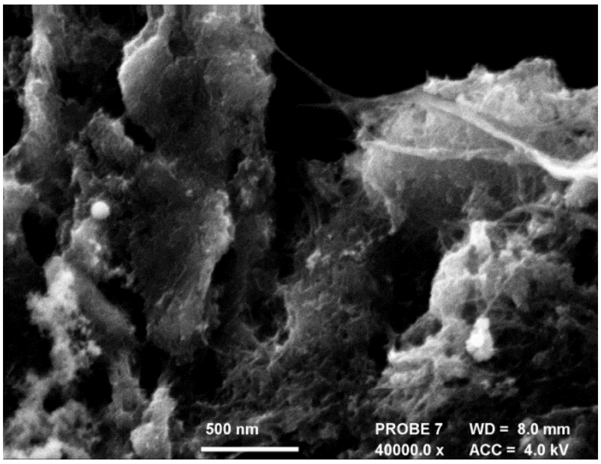

Figure S20: Magnified SEM image of covalently linked compound **1**@SWNTs complex, scale bar is 500nm.

The SEM measurements were carried out by dropping covalently linked complex solution (which was previously treated with prolonged sonication and centrifugation, at least 15 minutes) onto HPGO substrate. It can be seen from the SEM images that the linked Zn(II)-porphyrin consistently covered the surface of SWNTs. Compared to non-covalently linked functionalised complex the covalently linked complex presented a more porous structure.

9. Solid state investigation by AFM

Full surface morphology information of this complex nanohybrid material can be achieved by combining TEM, SEM and AFM information.

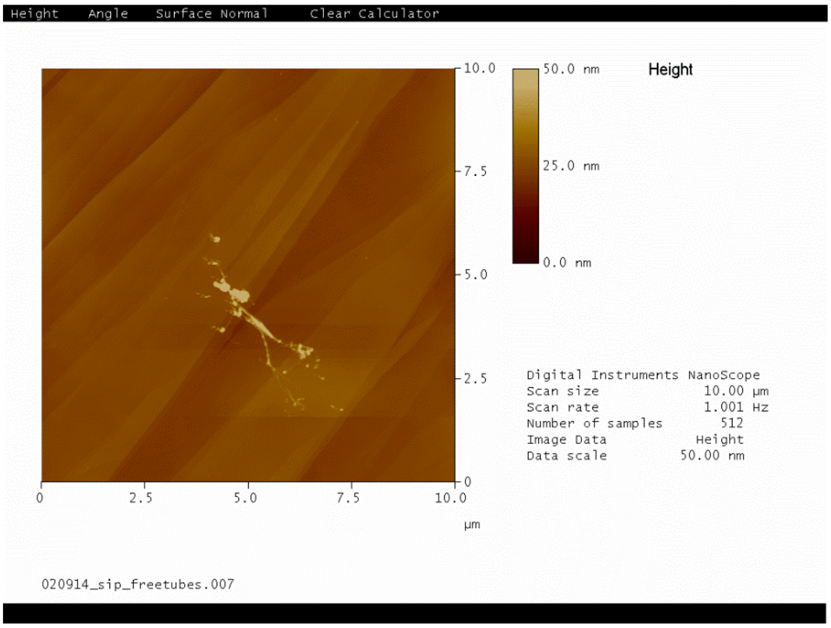

Figure S21: Tapping mode AFM image of raw SWNTs on HOPG

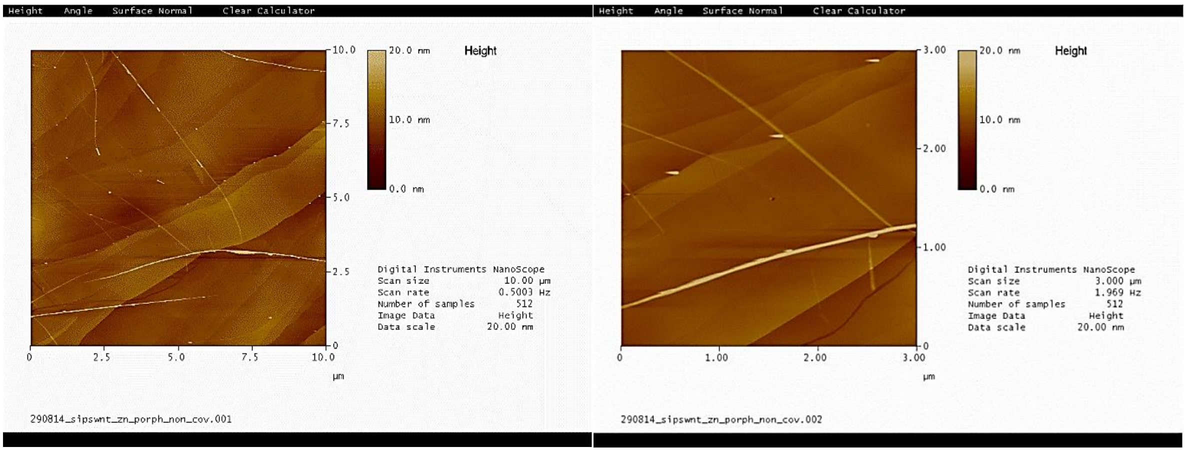

Figure S22: Tapping mode AFM image of non-covalently linked SWNT@Zn(II)-porphyrin complex (**2**) on HOPG.

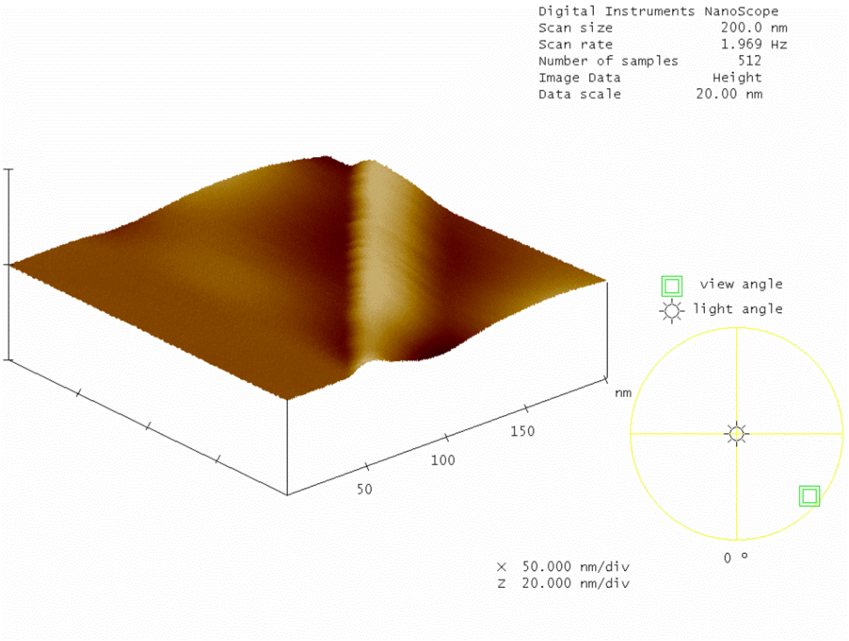

Figure S23: 3D Tapping mode AFM image of non-covalently linked SWNT@Zn(II)-porphyrin complex (**2**).

The AFM spectroscopy measurements of non-covalently linked compound **1**@SWNTs were carried out to further study the surface morphology information of the complexes.

For the non-covalently linked compound **1**@SWNTs complex, AFM measurement was introduced by dropping compound **1**@SWNTs complex dispersion solution onto a HOPG substrate, the mica substrate was also tested in the sample preparation progress with spin coating, but it did not seem to provide meaningful images.

The high qualified complex dispersion was prepared though prolonged sonication and centrifugations. The solvent combination for complex dispersion was 1:1 ethanol and chloroform at ca 1 mg/mL concentration, chosen not only to keep the continuity but also to be easily evaporated after dropping onto the HOPG substrate. From those images shown above, the compound **1** uniformly covers the entire aromatic surface of SWNTs and the surface decorated SWNTs could be relatively better dispersed compared to the raw intact SWNTs, for which it has not been possible to obtain meaningful images. The 3D AFM image indicated the fact that the individual functionalised carbon nanotube has ca. 9 nm height, although taller objects were identified and could not be imaged.

The AFM measurement was carried out via dropping the complex suspension onto a HOPG substrate. Because of the different electron charges and hydrophilic properties, a mica substrate measurement was attempted but failed in AFM sample preparation and characterisation process. Compared with non-covalently linked complex which presented a ca. 9 nm height, the covalently complex presented a slightly higher height. It is reasonable to assume that the height difference between covalently and non-covalently linked complex was due to the different linking space.

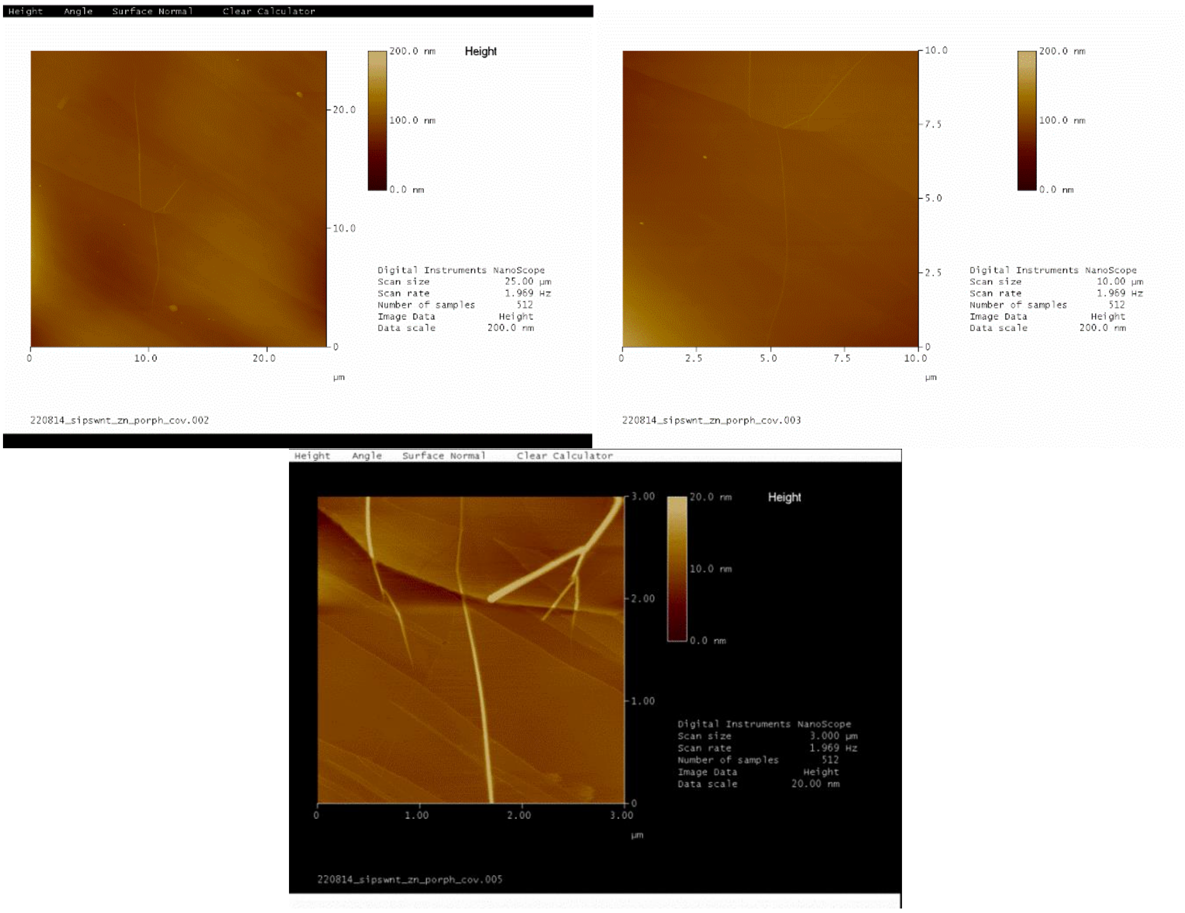

Figure S24: Tapping Mode AFM image of covalently linked compound **1**@SWNTs complex.

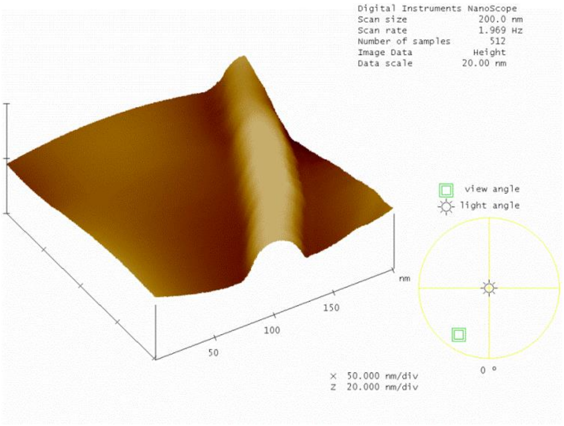

Figure S25: 3-Dimensional Tapping Mode AFM image of covalently linked compound **1**@SWNTs complex.

10. FTIR Spectroscopy characterization

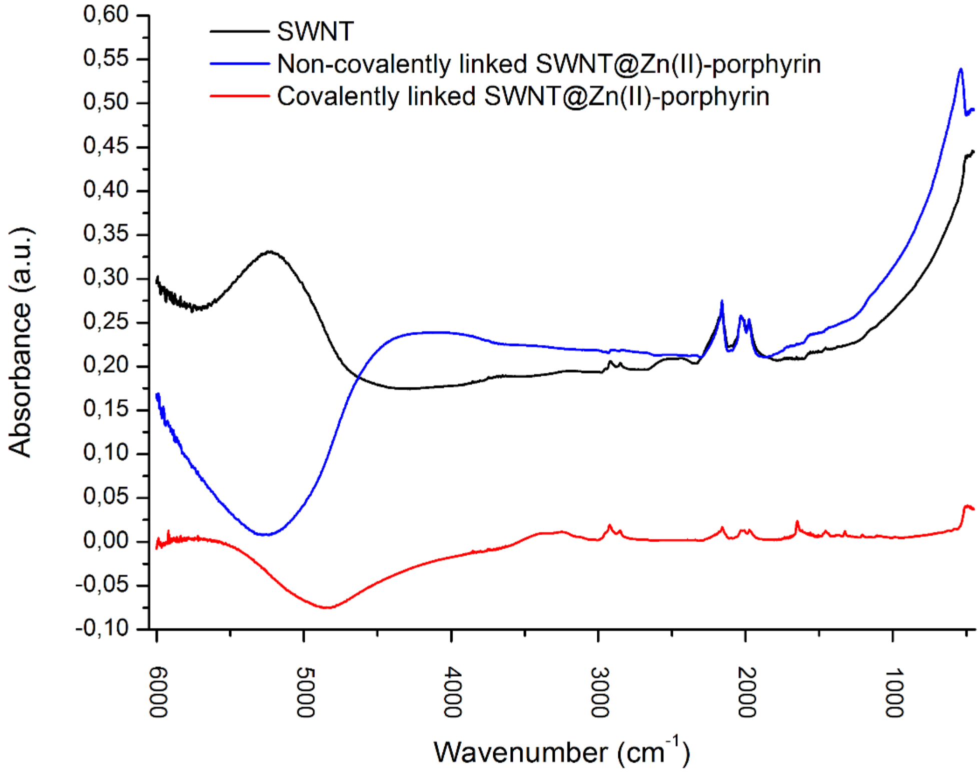

Figure S26: FTIR spectroscopy of SWNTs (recorded on the same samples as those analysed by AFM, above), non-covalently linked compound 1@SWNTs complex and covalently linked compound 1@SWNTs. The solvent mixture FTIR spectrum (1:1 ethanol and chloroform) was subtracted from each spectrum.

11. Raman Spectroscopy characterization

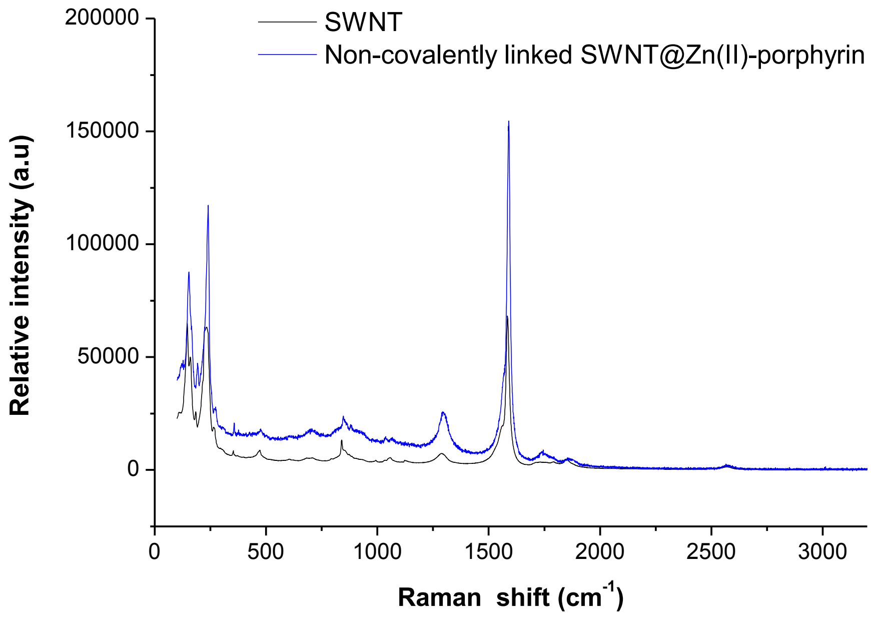

Figure S27: Solid state Raman spectroscopy of SWNTs and non-covalently linked compound **1**@SWNTs complexes (830 nm).

The Raman spectra of non-covalently linked compound **1**@SWNTs complex and intact SWNTs were recorded by dispersing samples in $CHCl_3$: EtOH (1:1) and using λ_{ex} = 830nm as at this wavelength the fluorescence emission from compound **1** will not interfere with the Raman spectroscopy charactering result.

For intact SWNTs, the G band at 1581 cm^{-1} (primary graphitic mode, corresponds to a splitting of the E2g stretching mode of graphite and reflects the structural intensity of the sp^2-hybridized carbon atoms) and G band at 1289 cm^{-1} (defect band, indicates the disordered graphite structure introduced by surface defects) of pure SWNTs can be observed. The I_D/I_G ratio of the intact SWNTs was 0.11 which was a relatively small ratio, indicating there were small amount of impurities or surface defects on the SWNTs.

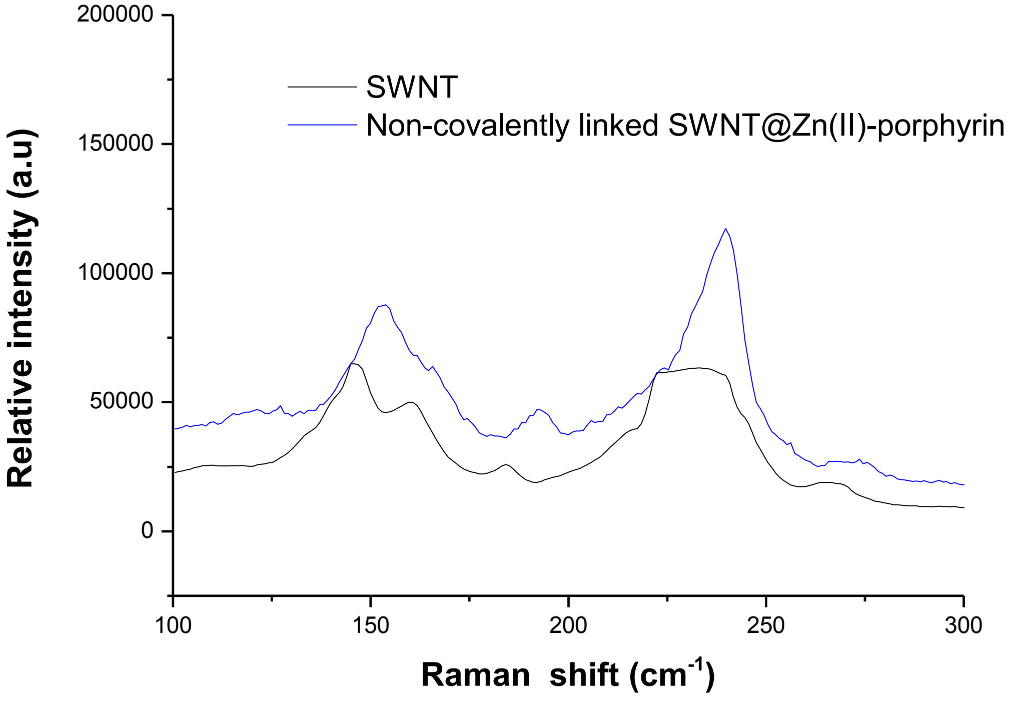

Figure S28: Selected RBM region in the solid state Raman spectroscopy of SWNTs and non-covalently linked compound **1**@SWNTs complex.

The Raman spectra of non-covalently linked compound **1**@SWNTs complex shows a G band located at 1588 cm^{-1} and a D band located at 1292 cm^{-1}. A I_D/I_G band intensity ratio of ca 0.17 was observed from the Raman spectra of non-covalently linked compound **1**@SWNTs complex. Comparing with the I_D/I_G band intensity ratio with free SWNTs, the ratio of non-covalently linked complex presented a nearly 50% increase, which indicated functionalisation of SWNTs with compound **1** has occurred.

The Raman spectra also show Radial breathing mode (RBM) peaks which are located between 140 cm^{-1} and 300 cm^{-1}. The enlarged regions in the Raman spectra above show that the free SWNTs samples contained carbon nanotubes with a broad diameter ranged from 0.8 nm to 1.8 nm, in which there were two significant and sharping RBM peaks at 234 cm^{-1} and 146 cm^{-1}. These sharp peaks indicate the nanotubes have diameters of 1.02 nm and 1.64 nm which is consistent with the DFT calculations performed.

The non-covalently linked compound **1**@SWNTs complex show two maxima RBM peaks at 240 cm^{-1} and 154 cm^{-1}, which indicates that in $CHCl_3$: EtOH (1:1) solution, the non-

covalently linked compound **1**@SWNTs complex dispersions contain tubes with 0.99 nm and 1.55 nm diameters.

The diameter information reflected by Raman RMB mode corresponded to the radial breath diameter of those tubes. It can be found that after functionalisation and coated with compound **1**, the breathing modes of carbon nanotube are being modified. Tt can be reasonably assumed that the compound **1** in the complex adheres strongly to the tubes surface generating a slightly red shift at SWNTs RBM within compound **1**@SWNTs complex: it further supports the hypothesis that the supramolecular complex emerges from this interaction between SWNTs and compound **1**.

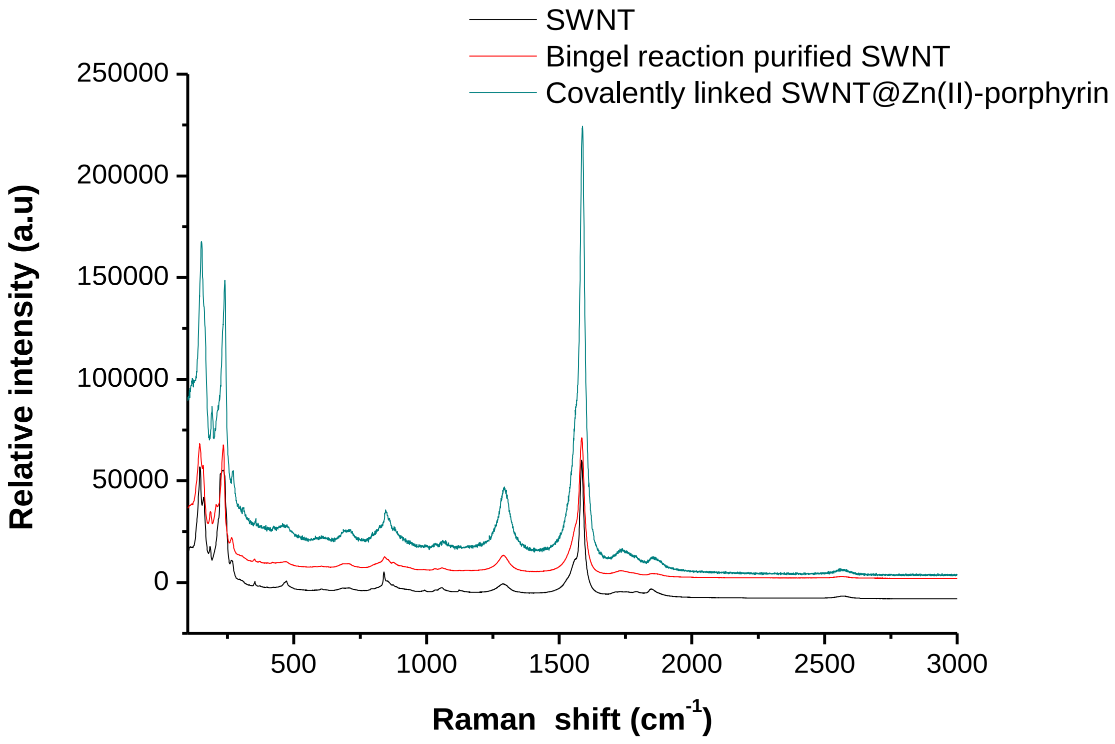

Figure S29: Raman spectroscopy of SWNTs, Bingel reaction functionalised SWNTs precursor and covalently linked compound **1**@SWNTs complex (excitation at 830 nm).

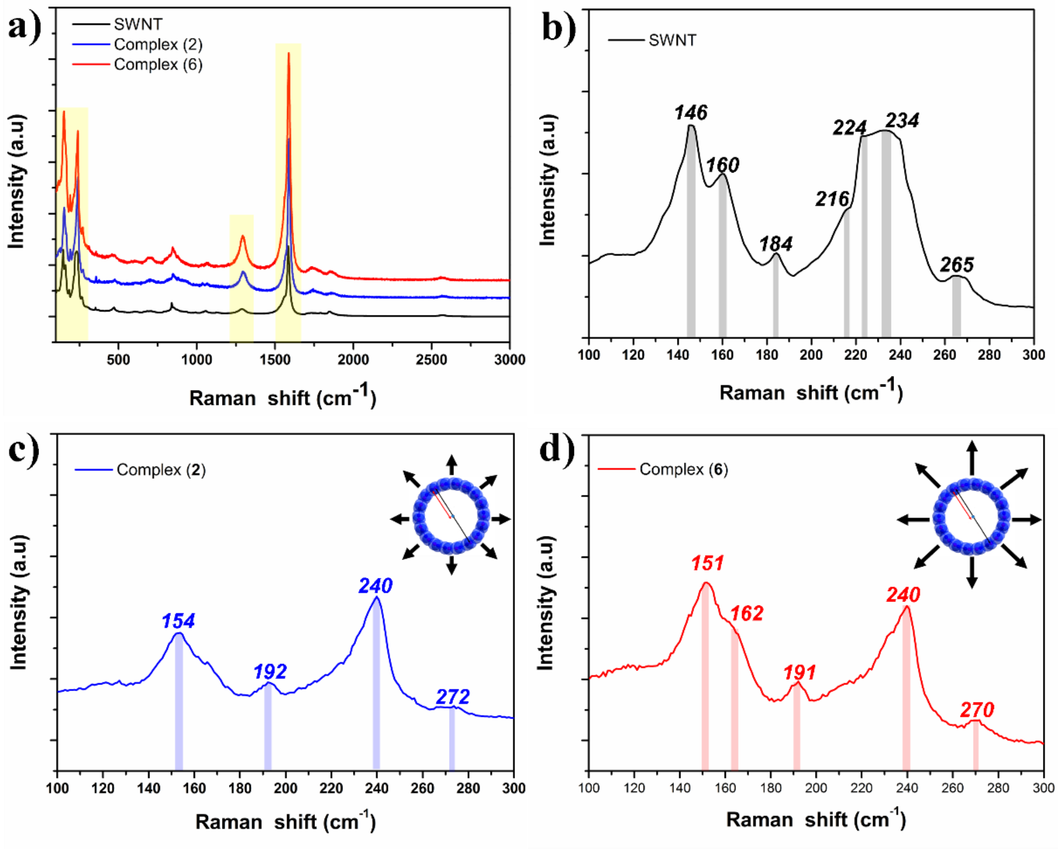

Figure S30: Overview of the Raman spectroscopy of SWNTs, Bingel reaction functionalised SWNTs (Complex **2**) and non-covalently linked compound **1**@SWNTs complex (Complex **6**).

Table S3: A comparison of ID/IG band intensity observed for the different types of single-walled carbon nanotubes and their complexes.

**The type of single-walled nanotube or their complex**	**I_D/I_G**
Single-walled nanotubes (raw material)	0.11
Non-covalently linked compound **1**@SWNTs complex	0.17
SWNTs functionalised by the Bingel reaction	0.16
Covalently linked compound **1**@SWNTs complex	0.19

The intact SWNTs Raman spectra presents the G band at 1581 cm^{-1} (primary graphitic mode) and G band at 1289 cm^{-1} (defect band). Meanwhile, the Raman spectra of precursor emerging after the Bingel reaction containing functionalised SWNTs exhibits a G band at 1585 cm^{-1} and a D band at 1289cm^{-1}. It is commonly accepted that the I_D/I_G band intensity ratio reflects the degree of crystallinity of graphitic carbon structure in carbon nanotubes. It can be seen that the I_D/I_G band intensity ratio of Bingel reaction functionalised SWNTs (0.16)

has been an increase compared to the I_D/I_G band intensity ratio of the intact free SWNTs (0.11), which indicated that Bingel reaction constitutes a source of surface defects and induces the formation of some disordered graphite structures onto the surface of SWNTs.

Compared to non-covalently linked compound **1**@SWNTs complex, the Raman spectra of covalently linked complex exhibited a G band locates at 1588 cm^{-1} and a D band locates at 1293 cm^{-1} and a slightly increased I_D/I_G band intensity ratio of 19%. Compared the I_D/I_G band intensity ratio of intact free SWNTs with the ratio of both non-covalently linked complex (0.17) and covalently linked complex (0.19), it has been confirmed that the supramolecular interaction and the covalently linked interactions have occurred.

The free SWNTs contains tubes with a broad diameter ranging from 0.8 nm to 1.8 nm, where there were two significant and sharping RBM peaks appeared at 234 cm^{-1} and 146 cm^{-1}. The spectrum of Bingel reaction functionalised SWNTs remained almost unchanged with respect to free SWNTs, exhibited two maxima RMB peaks at 234 cm^{-1} and 145 cm^{-1}, pointing to the tubs with calculated diameters of 1.02 nm and 1.64 nm. Compared to the intact free SWNTs, the maxima RBM peaks of covalently linked compound **1**@SWNTs complex presented a red shifted peaks at 240 cm^{-1} and 152 cm^{-1}. Those RBM peaks indicating the tubes with calculated diameter of 0.99 nm and 1.57 nm were mainly presented. The diameter information reflected by Raman RMB mode was corresponded to the radial breaking diameter of carbon nanotubes. It could be found that after modifications by Bingel reaction, the diameter of modified SWNTs did not present significant changes compared with free (intact) SWNTs used. Taken the Bingel reaction I_D/I_G band intensity ratio into consideration, it can be reasonably claimed that the Bingel reaction introduce cyclopropane structure onto the surface of SWNTs, but this disorder defects did not significantly affect the tubes breath-ability.

After the covalent linkage with Zn(II)-porphyrin, the RBM mode was affected, e.g. the ‘breath ability’ of the functionalised carbon nanotube was reduced. This could be lead to the reasonable assumption that the Zn(II)-porphyrin in the complex was successfully linked on the tube surface. A decrease of the calculated diameter covalently linked compound **1**@SWNTs complex indicated there were interactions within SWNTs and Zn(II)-porphyrin and also the formation of covalently linked SWNTs@Zn(II)-porphyrin complex. Compared with the non-covalently and covalently linked strategies, both complexes showed shifts at the RBM bands as described in Figure S30, and the calculated diameters (see above) for both complexes were similar.

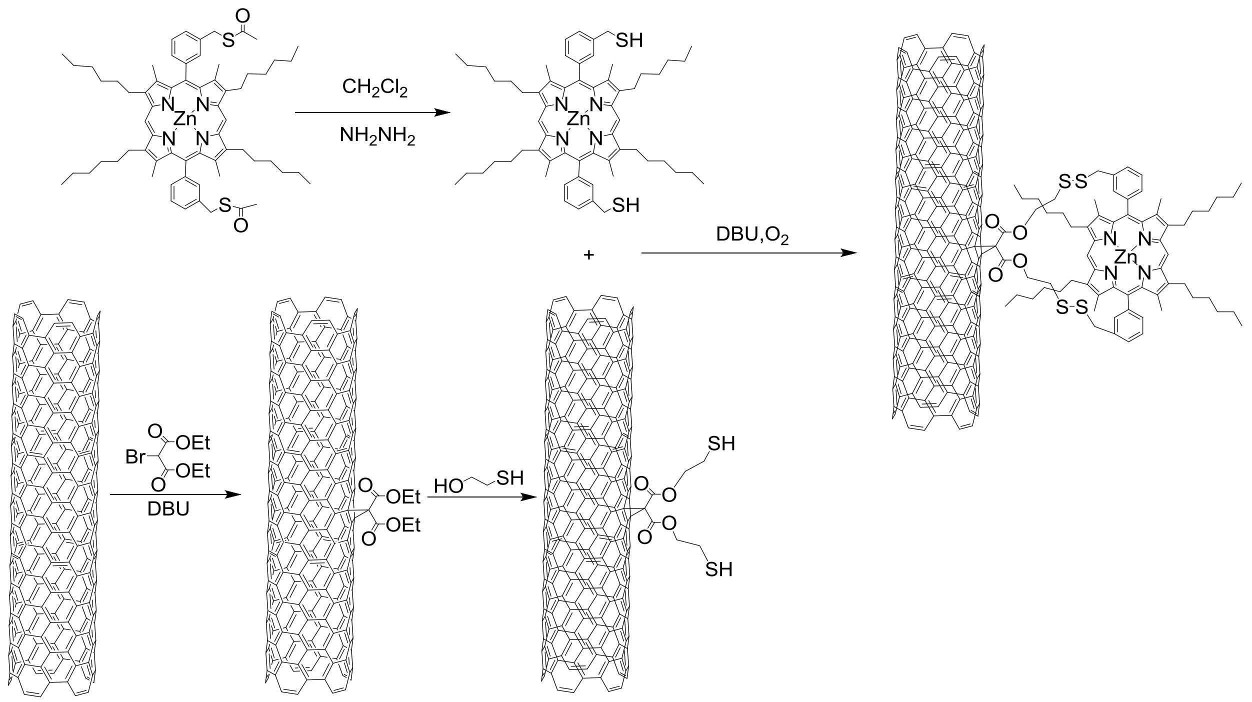

Scheme S3: Schematic diagram of generation of covalently linked compound **1**@SWNT complex.

12. Gas phase DFT calculations

Table S4. Energies and electronic occupancy of the highest occupied and lowest unoccupied molecular orbitals of Zn(II)-porphyrin from gas phase optimisations (DFT).

Molecular Orbital	Occupancy	E (eV)
**LUMO + 5**	0	-0.26
**LUMO + 4**	0	-0.32
**LUMO + 3**	0	-0.50
**LUMO + 2**	0	-0.53
**LUMO + 1**	0	-1.86
**LUMO**	0	-1.87
**HOMO**	2	-4.82
**HOMO – 1**	2	-4.93
**HOMO – 2**	2	-5.90
**HOMO – 3**	2	-5.94
**HOMO – 4**	2	-5.97
**HOMO – 5**	2	-6.09

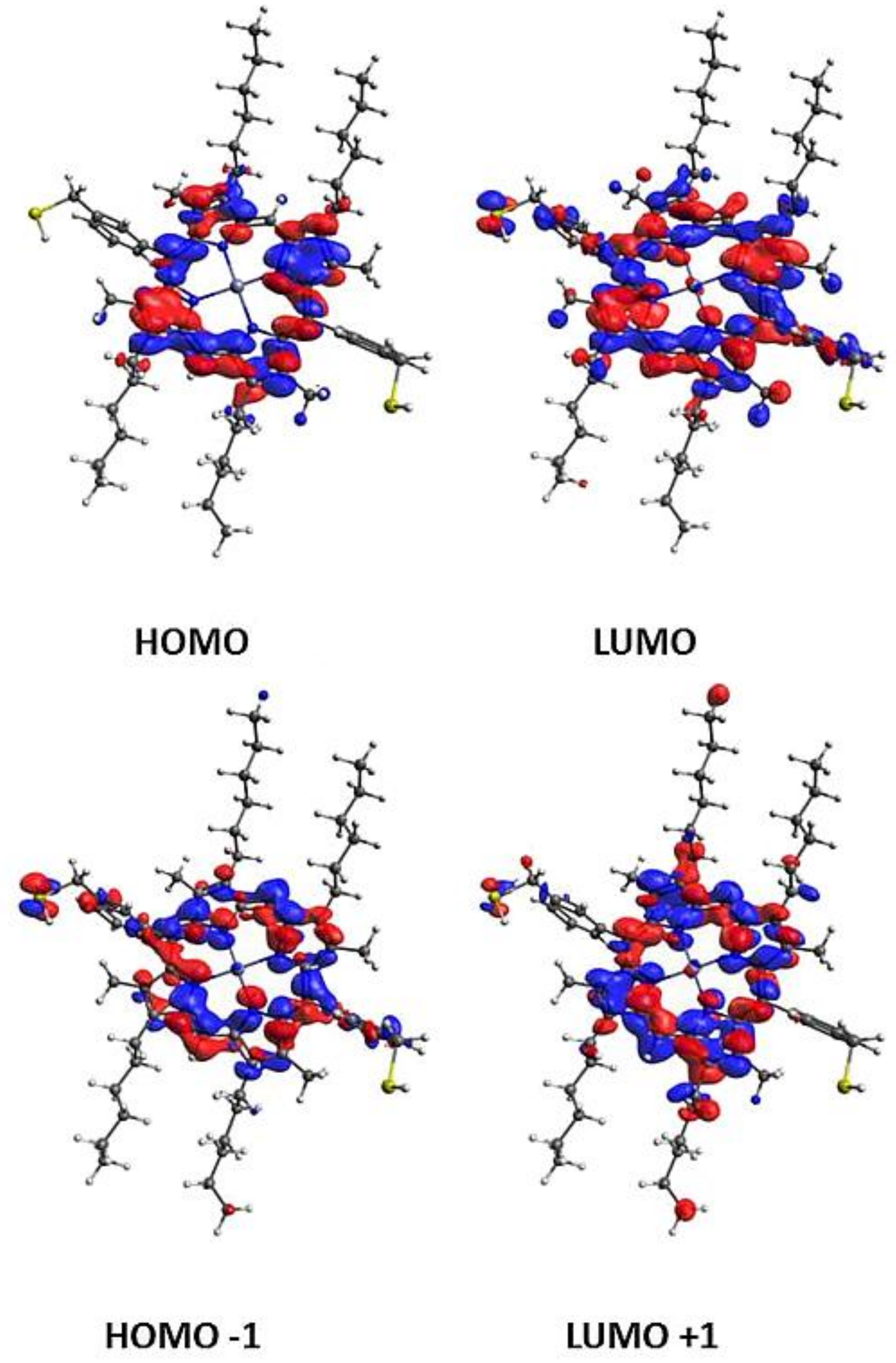

Figure S31. Two highest occupied and lowest unoccupied molecular orbital of deprotected Zn(II)-porphyrin (3) from gas phase DFT calculations.

13. UV-vis titrations and fitting of association binding constant.

In order to confirm that the π-π interactions between porphyrin and carbon single-walled nanotubes and quantify their supramolecular assembling, a host-guest UV-Vis titration[6] was carried out by using coronene, $C_{24}H_{12}$, coronene, (termed Guest) as model for sp^2 carbon system and Zn-Porphyrin (termed Host). The formation of these HG 1:1 stoichiometry can be described by the following bimolecular equilibrium:

$$H + G \rightleftharpoons HG$$

The stepwise binding 1:1 constants therefore can be described as:

$$K_{1:1}\frac{[HG]}{[H][G]}$$

The mathematical model associated with UV-vis titrations and used to obtain the binding constant is developed by correlating the [HG] to the variation of UV-Vis absorbance intensity (ΔA).

$$\Delta A = \varepsilon_{\Delta HG}[HG]$$

In this experiment, a stock solution of Zn-Porphyrin (5×10^{-6} M) and Coronene (5×10^{-6} M) were prepared in a mixture of chloroform and toluene (1:1). Separately, a solution of Zn-Porphyrin (5×10^{-6} M) was prepared in a same mixture solution. Stock solution was gradually added (65 μL) into the cuvette containing Zn-Porphyrin, so that the concentration of Zn-Porphyrin for each UV-Vis experiment was kept constant. The UV-Vis absorption of Porphyrin increased as the equivalents of coronene were progressively added. These absorption values were collected in a data set (excel files or input files). Then, such data set was analyzed by MATLAB m-files and fitted for 1:1, Zn-Phorphyrin:coronene binding isotherm. This software resulted in $K_{1:1}$ and statistic parameters such as statistic error (SEy) and covariance of fitting (Cov_f). Binding constants and statistic parameters are reported in Table S5

Table S5. Binding constant $K_a^{1:1}$ is in M^{-1}. Standard error of estimated data (SE*y*) and convariance of fit (Cov_f) are reported and calculated according reference[6]

	$K_{1:1}$ (M^{-1})	SEy	Cov_f
Zn-Phorphyrin	46200	0.0038	0.0032

The UV-vis titration data indicated enhanced absorptions relative to the peak at 421 nm and 412 nm. A broader peak at 546 nm was also observed in the non-covalently linked

complex filtrate, which corresponds to the secondary absorption band of intact free porphyrin. **Figure S27** shows the UV-vi absorption titration of coronene and Zn(II)-porphyrin while the insert image is the magnified UV-vis titration spectrum. Plots of the experimental ΔA of Zn(II)-Porphyrin at λ_{abs} = 412 nm (blue markers), λ_{abs} = 421 nm (red markers) and fitting curves (blue and red solid lines for 1:1 fitted isotherm) are also reported hereby.

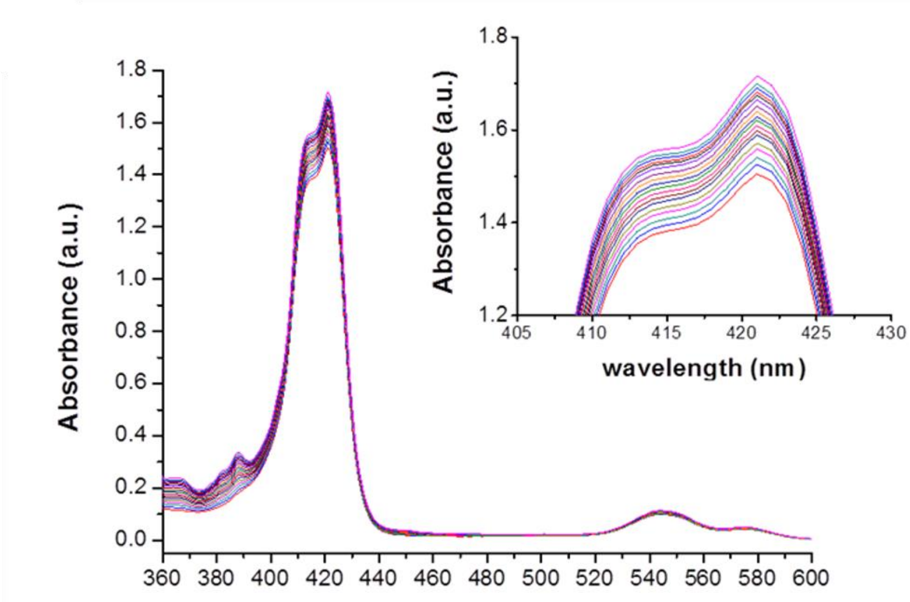

Figure S32. (a). UV-visible titration experiment of coronene (5×10^{-6} M) into a Zn(II)-porphyrin solution (5×10^{-6} M). The solutions were prepared in a 1:1 mixture of chloroform and toluene. The inset image is the magnified UV-vis titration spectrum.

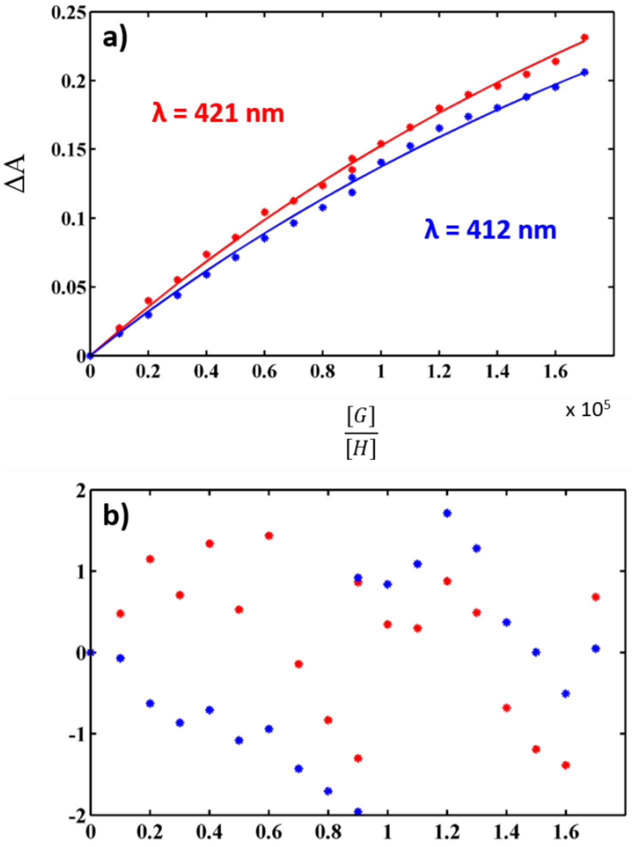

Figure S33. a) Solid markers show variation of the variation of absorbion intesity duiring the UV-vis titration . Red and blue lines show the fitting 1:1 binding module for λ = 421 nm and λ = 412 nm respectively. b) Standard deviation of the set absorbion, $([\Delta A]_{exp})$ - $([\Delta A]_{exp})$, values.

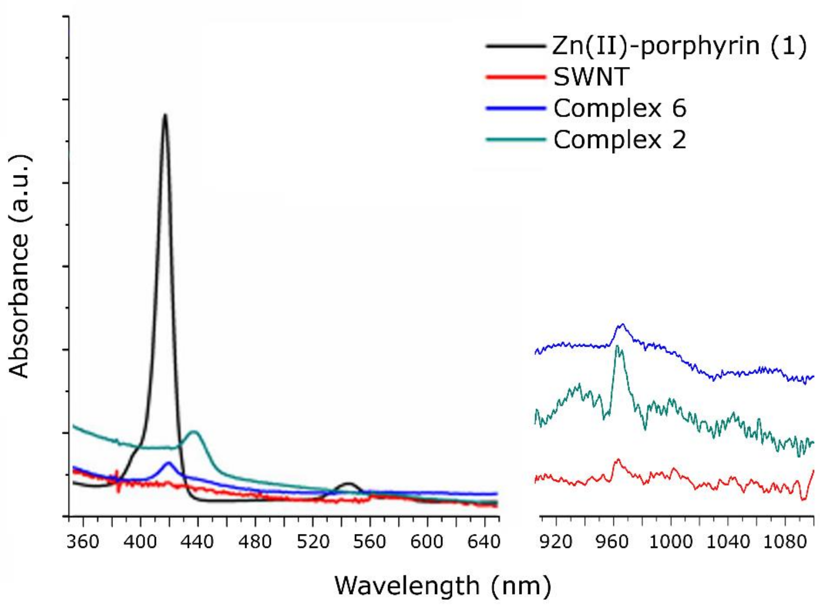

Figure 34. **U**V-vis and NIR spectra of Zn(II)-porphyrin, intact SWNTs, non-covalent Zn(II)-porphyrin@SWNTs nanohybrids (2) and covalent Zn(II)-porphyrin@SWNTs nanohybrids

14. Single-photon laser confocal fluorescence

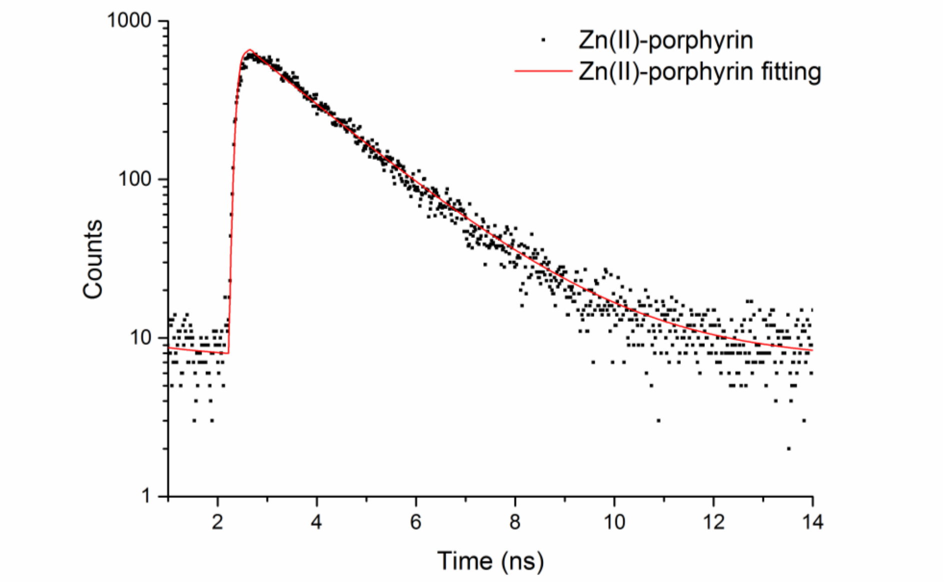

Figure S35. Time-Correlated Single Photon Counting (TCSPC) decay curves for Zn(II)-porphyrin in ethanol:chloroform, 1 μM (using single photon excitation λ_{ex} = 473 nm). χ^2 value of this curve is 1.49. The lifetime of measured Zn(II)-porphyrin sample are τ_1 = 1609.4 ps (90.7 %) and τ_2 = 2537.3 ps (9.3%) respectively.

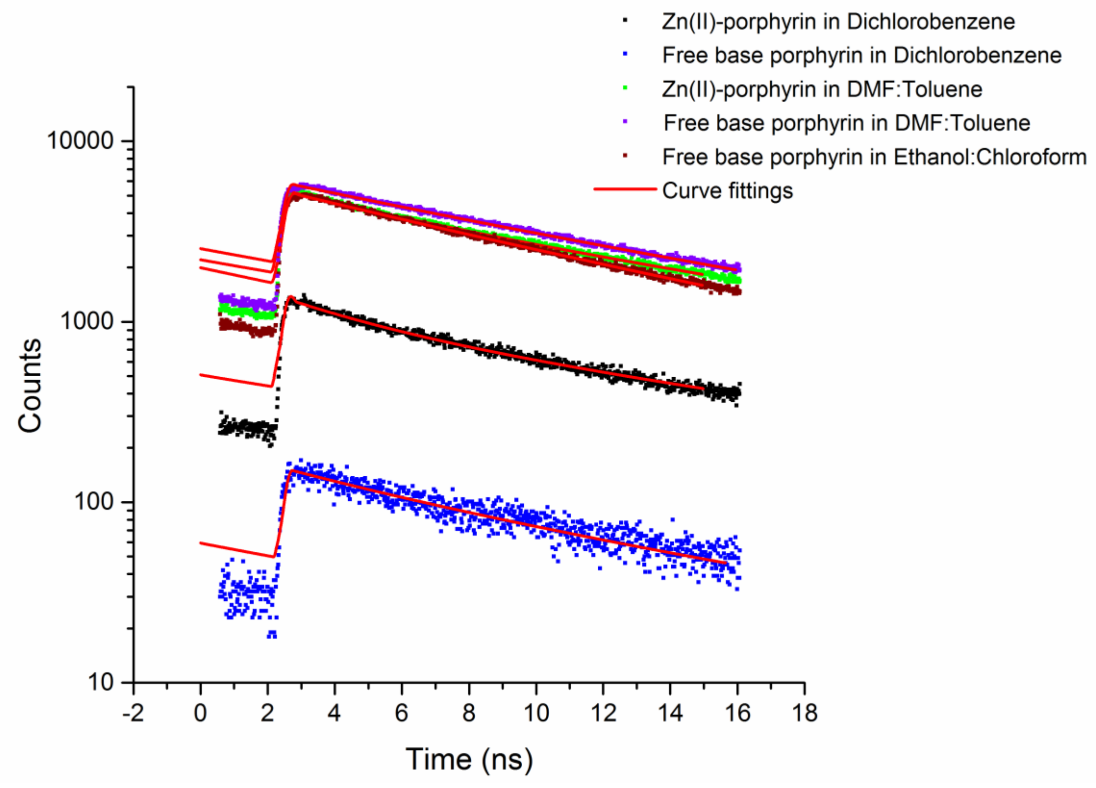

Figure S36. Time-Correlated Single Photon Counting (TCSPC) decay (using single photon excitation λ_{ex} = 473 nm) curves for: Zn(II)-porphyrin 1 μM in dichlorobenzene,. χ^2 value of this curve is 1.17. The lifetime of measured Zn(II)-porphyrin sample are τ_1 = 3325.5 ps

(48.2 %) and τ_2 = 17902.8 ps (51.8%) respectively; Zn(II)-porphyrin in DMF:toluene, χ^2 value of this curve is 1.32. The lifetime of measured Zn(II)-porphyrin sample are τ_1 = 5187.4 ps (45.2 %) and τ_2 = 19894.5 ps (54.8%) respectively; free base porphyrin in dichlorobenzene, χ^2 value of this curve is 1.38. The lifetime of measured free base porphyrin sample are τ_1 = 4715.6 ps (41.5 %) and τ_2 = 14503.3 ps (58.5 %) respectively; free base porphyrin in DMF:toluene, χ^2 value of this curve is 1.39. The lifetime of measured free base porphyrin sample are τ_1 = 7513.2 ps (50.29 %) and τ_2 = 18813.3 ps (49.7 %) respectively and free base porphyrin in ethanol:chloroform, χ^2 value of this curve is 1.47. The lifetime of measured free base porphyrin sample are τ_1 = 5848.0 ps (17.0 %), τ_2 = 8929.2 ps (57.8 %) and τ_3 = 25176.1 ps (25.2 %) respectively.

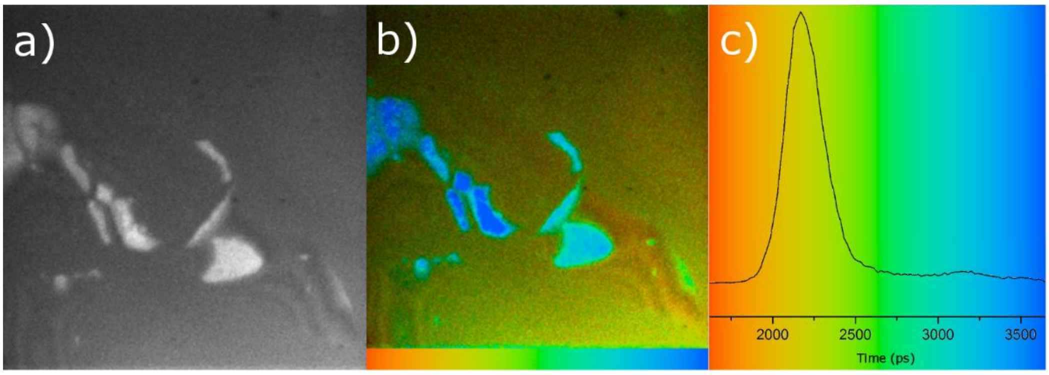

Figure S37. Single-photon laser confocal fluorescence imaging in thin films (λ_{ex}= 405 nm): (a) free base porphyrin thin film intensity image, (b) excited state lifetime mapping and scale bar (c) corresponding lifetime distribution curve across the image.

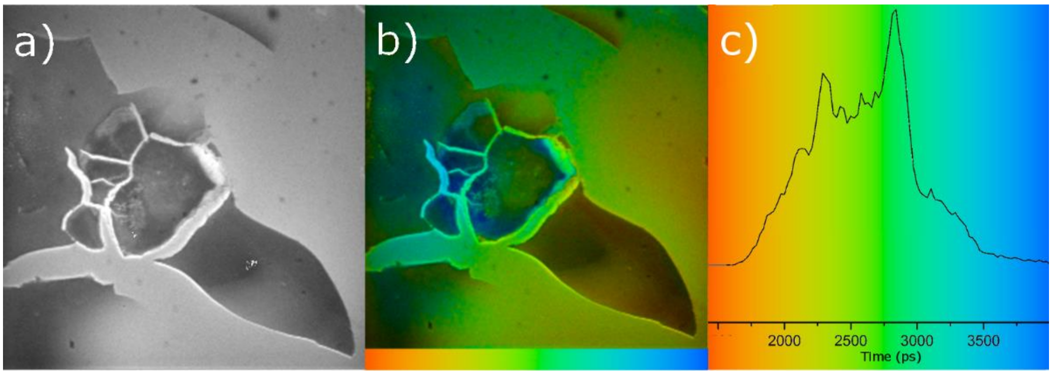

Figure S38. Single-photon laser confocal fluorescence in thin films (λ_{ex}= 473 nm): (a) free base porphyrin thin film intensity image, (b) excited state lifetime mapping and scale bar (c) corresponding lifetime distribution curve across the image.

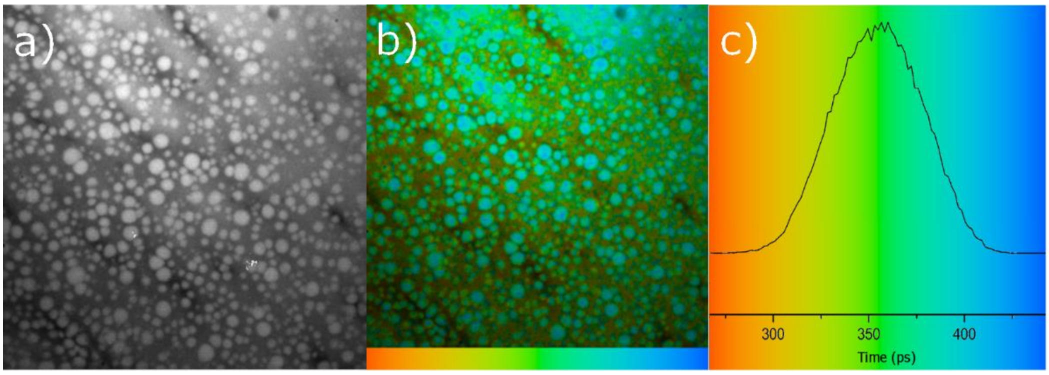

Figure S39. Single-photon laser confocal fluorescence (λ_{ex}= 473 nm): (a) Zn(II)-porphyrin thin film intensity image, (b) excited state lifetime mapping and scale bar (c) corresponding lifetime distribution curve across the image.

15. Two-photon laser confocal fluorescence

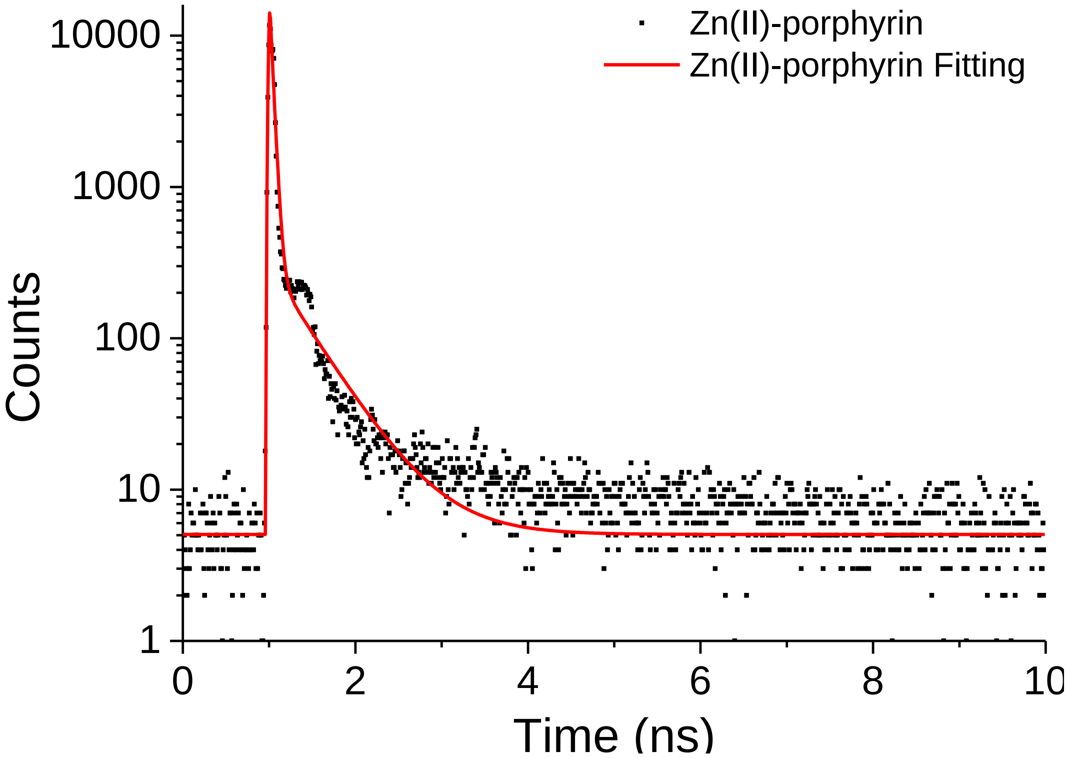

Figure S40. Time-Correlated Single Photon Counting (TCSPC) point decay curves for a solution of Zn(II)-porphyrin 1 μM in DMF:Toluene (Two photon excitation λ_{ex} = 910 nm); laser power was in between 4.0 – 7.0 mW.

Figure S14 represents the TCSPC decay curve for Zn(II)-porphyrin in Chloroform, 1 μM, from a two-photon excitation confocal microscopy. The data suggested that there were

significant porphyrin aggregations, which accounts for the short lifetime decay and high fluorescence intensity. The lifetime of measured Zn(II)-porphyrin sample are τ_1 = 35 ps (98.7 %) and τ_2 = 476.1 ps (1.3%) respectively (910 nm), but chi^2 was larger than 1.5. The experiment was therefore carried out at 810 nm 2P excitation instead, which was found to be significantly more suitable for this porphyrin system (vide infra).

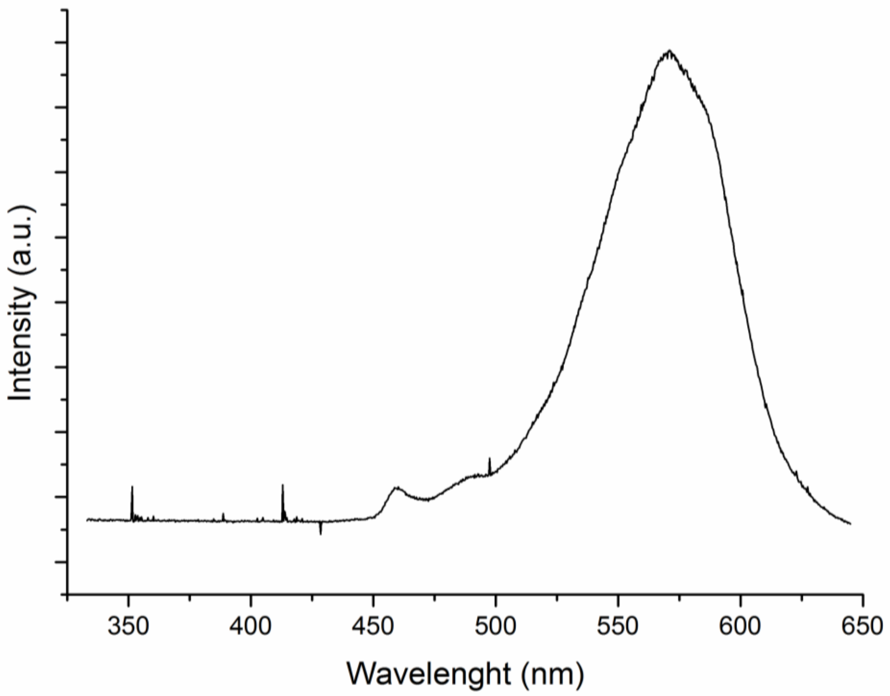

Figure S41. Two-photon laser confocal fluorescence spectrum of Zn(II)-porphyrin, 1 μM in DMF:Toluene (λ_{ex}= 910 nm).

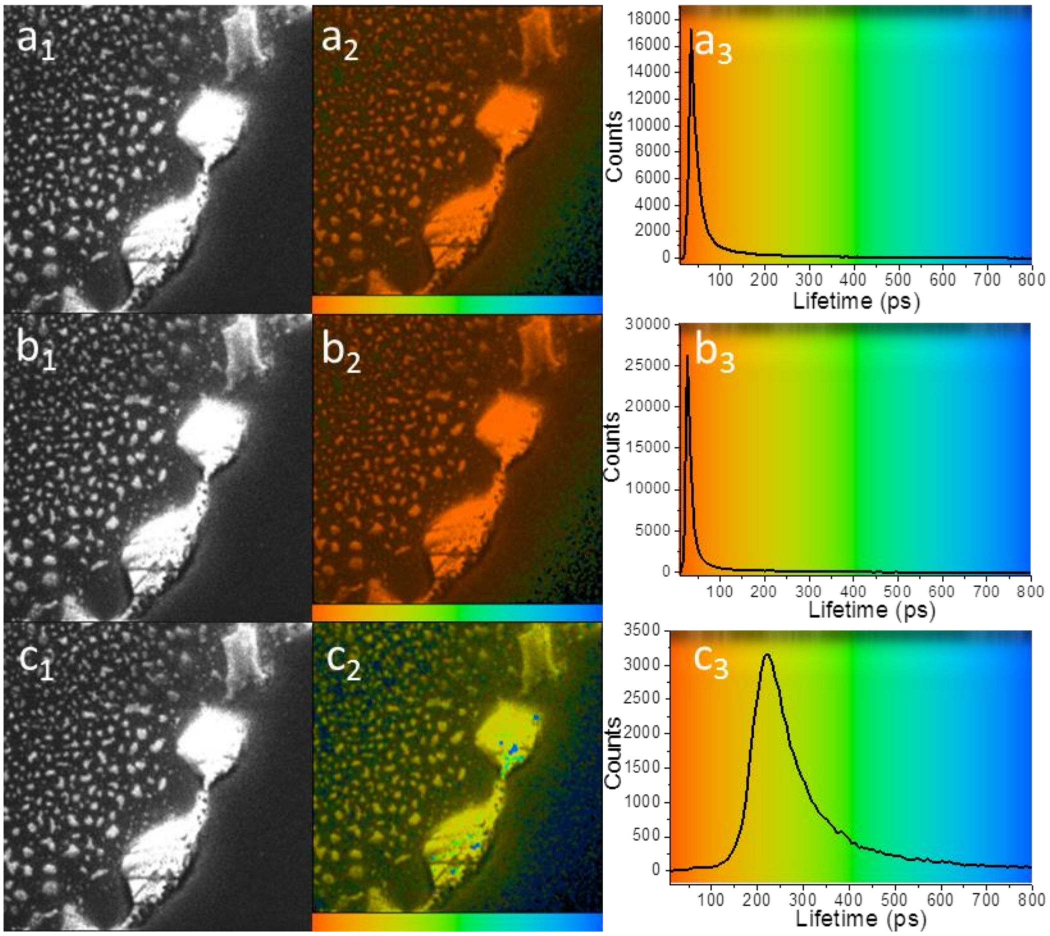

Figure S42. Two-photon FLIM (λ_{ex}= 810 nm): (a_1) Free base porphyrin intensity image of τ_m, (b_1) excited state lifetime mapping and scale bar of τ_m (c_1) corresponding lifetime distribution curve across the image of τ_m. (a_2) Free base porphyrin intensity image of τ_1, (b_2) excited state lifetime mapping and scale bar of τ_1 (c_2) corresponding lifetime distribution curve across the image of τ_1. (a_3) Free base porphyrin intensity image of τ_2, (b_3) excited state lifetime mapping and scale bar of τ_2 (c_3) corresponding lifetime distribution curve across the image of τ_2.

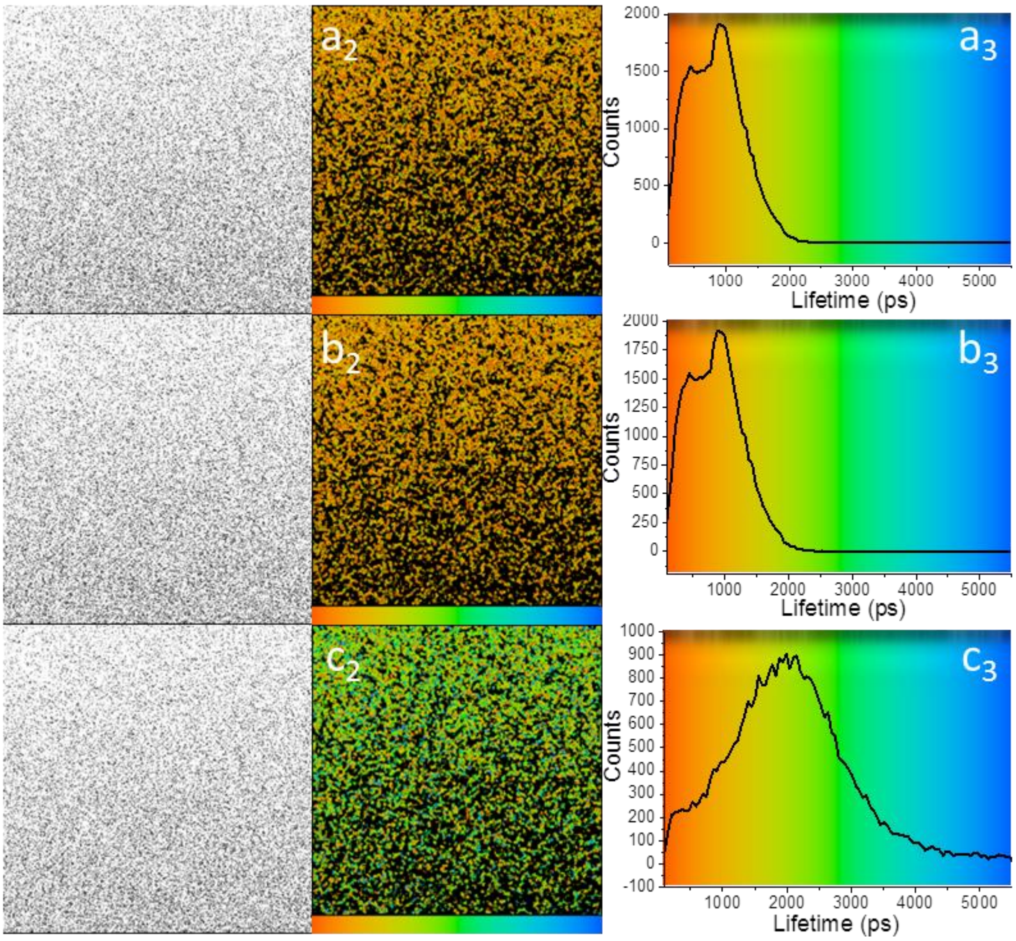

Figure S43. Two-photon FLIM (λ_{ex}= 810 nm): (a_1) Zn(II)-porphyrin intensity image of τ_m, (b_1) excited state lifetime mapping and scale bar of τ_m (c_1) corresponding lifetime distribution curve across the image of τ_m. (a_2) Zn(II)-porphyrin intensity image of τ_1, (b_2) excited state lifetime mapping and scale bar of τ_1 (c_2) corresponding lifetime distribution curve across the image of τ_1. (a_3) Zn(II)-porphyrin intensity image of τ_2, (b_3) excited state lifetime mapping and scale bar of τ_2 (c_3) corresponding lifetime distribution curve across the image of τ_2.

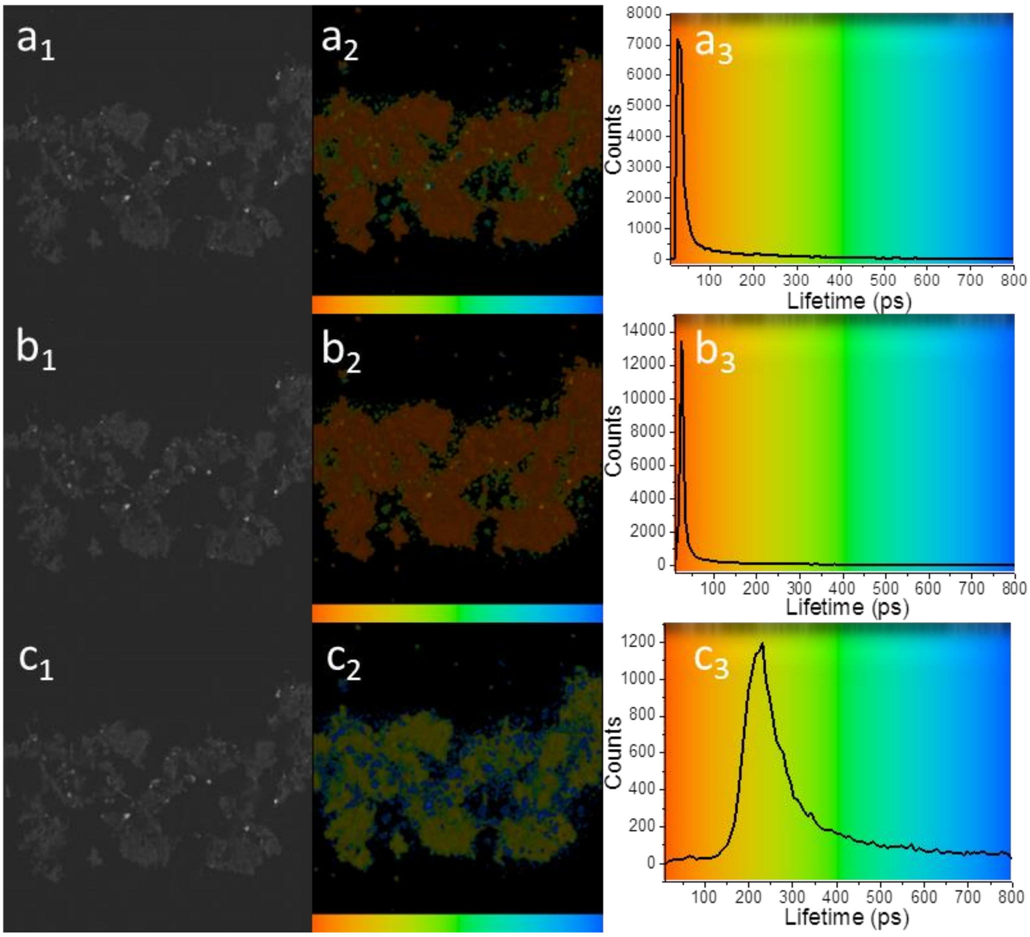

Figure S44. Two-photon FLIM (λ_{ex}= 810 nm): (a_1) Covalent functionalised porphyrin intensity image of τ_m, (b_1) excited state lifetime mapping and scale bar of τ_m (c_1) corresponding lifetime distribution curve across the image of τ_m. (a_2) Covalent functionalised porphyrin intensity image of τ_1, (b_2) excited state lifetime mapping and scale bar of τ_1 (c_2) corresponding lifetime distribution curve across the image of τ_1. (a_3) Covalent functionalised porphyrin intensity image of τ_2, (b_3) excited state lifetime mapping and scale bar of τ_2 (c_3) corresponding lifetime distribution curve across the image of τ_2.

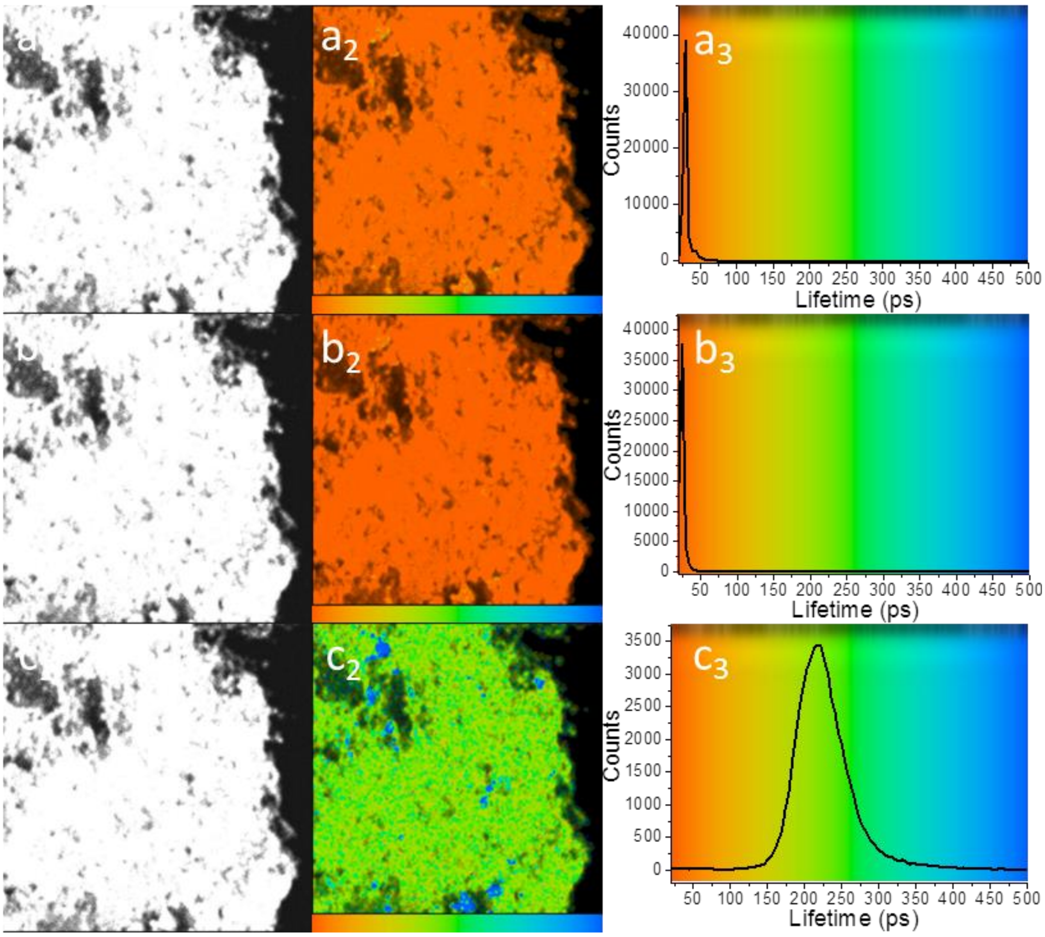

Figure S45. Two-photon FLIM (λ_{ex}= 810 nm): (a_1) Non-covalent functionalised porphyrin intensity image of τ_m, (b_1) excited state lifetime mapping and scale bar of τ_m (c_1) corresponding lifetime distribution curve across the image of τ_m. (a_2) Non-covalent functionalised porphyrin intensity image of τ_1, (b_2) excited state lifetime mapping and scale bar of τ_1 (c_2) corresponding lifetime distribution curve across the image of τ_1. (a_3) Non-covalent functionalised porphyrin intensity image of τ_2, (b_3) excited state lifetime mapping and scale bar of τ_2 (c_3) corresponding lifetime distribution curve across the image of τ_2.

Table S6. Two-photon solid state FLIM for free base porphyrin, Zn(II)-porphyrin (1), non-covalent Zn(II)-porphyrin@SWNTs complex (2) and Zn(II)-porphyrin@SWNTs (covalent) complex (6), 810 nm excitation.

	χ^2	a_1 (%)	τ_1 (ps)	a_2 (%)	τ_2 (ps)	τ_m (ps)
Free base porphyrin	1.30	84.1	244.0±26.0	15.9	3369.7±460.1	742.0±155.9
1	1.17	87.3	554.0±55.0	12.7	1715.6±231.6	765.3±96.6
2	1.49	98.8	35.0±4.5	1.2	870.8±75.1	44.9±6.1
6	1.15	98.7	28.0±11.8	1.3	6880.1±228.0	120.5±67.1

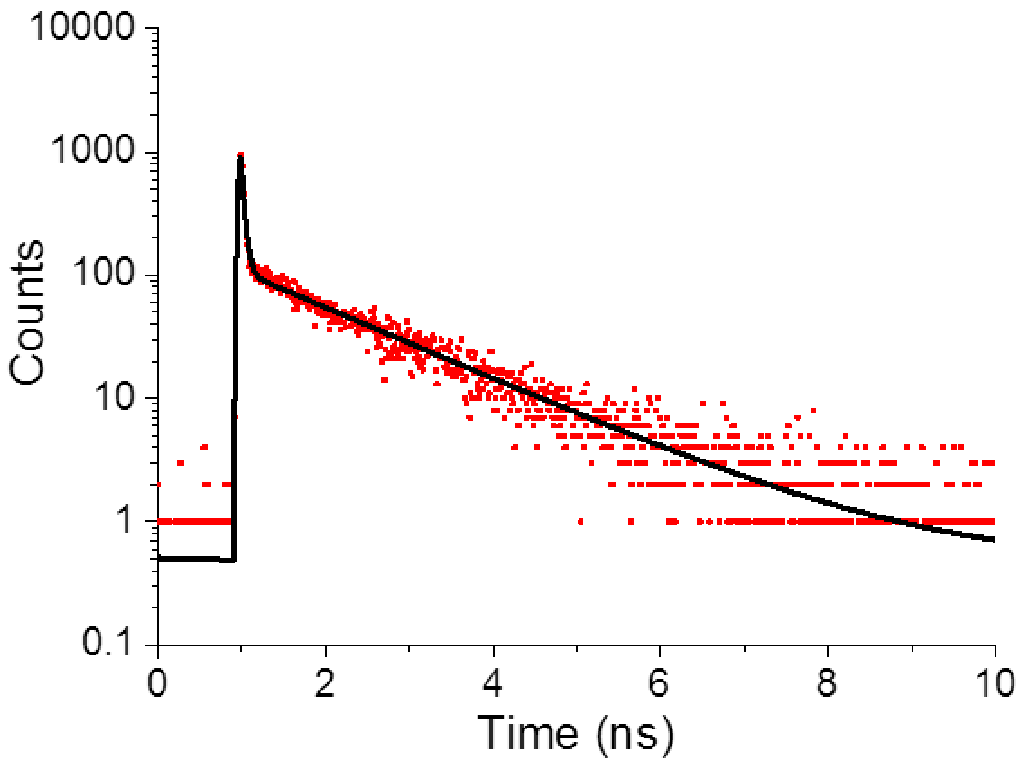

Figure S46. Time-Correlated Single Photon Counting (TCSPC) (2 photon excitation, λ_{ex}= 810 nm) of Zn(II)-porphyrin 1 μM in DMF:Toluene. Laser power was 5.4 mW.

Table S7. Two-photon solution state TCSPC for Zn(II)-porphyrin (810 nm),

	χ^2	τ_1 (ps)	a_1 (%)	τ_2 (ps)	a_2 (%)	τ_m (ps)
Zn(II)-porphyrin	1.50	39±5.0	93.2	1483.5±2.8	6.8	136.67

16. STED Super Resolution Microscopy

3D STED Microscopy was carried out on a Leica TCS SP8 3X Gated STED (Leica, Mannheim, Germany).[7] The microscope is equipped with a pulsed supercontinuum white light excitation laser at 80Mhz (NKT, Denmark), and a continuous wavelength STED lasers at 592nm and a pulsed at 775nm. Experiments with Zn(II)Porphyrin (**1**) were done exciting at 488nm, their emission was depleted at 775nm, and the emission was collected around 580nm employing Leica HyD detectors. For the STED 3D reconstruction, each individual XY plane of the whole structure was scanned using the same conditions as mentioned above. Each individual XY plane image was deconvolved using the Huygens Software package (SVI, Netherlands), the final surface rendered 3D reconstruction was done employing the 3D imaging Leica software, LAX.

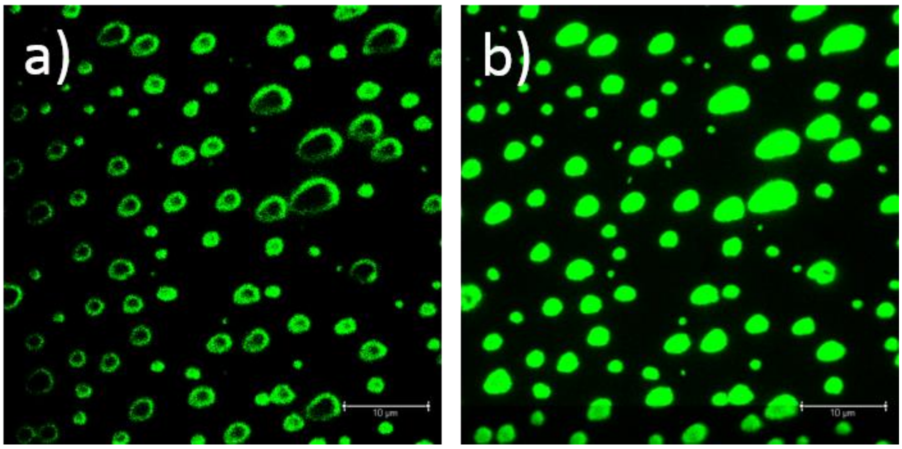

Figure S47. Stimulated emission depletion (STED) conditions (a) and confocal fluorescence microscopy (b) of Zn(II)-porphyrin (**1**) thin film of a $CHCl_3$ solution drying out on a horizontal surface of borosilicate glass. a) STED 3D rendering, λ_{ex} = 488.0 nm STED = 775 nm, intensity = 150mW, λ_{em} = 580; b) λ_{ex} = 488, λ_{em} = 580. Scalebar: 10 µm.

17. The coronene challenge experiment

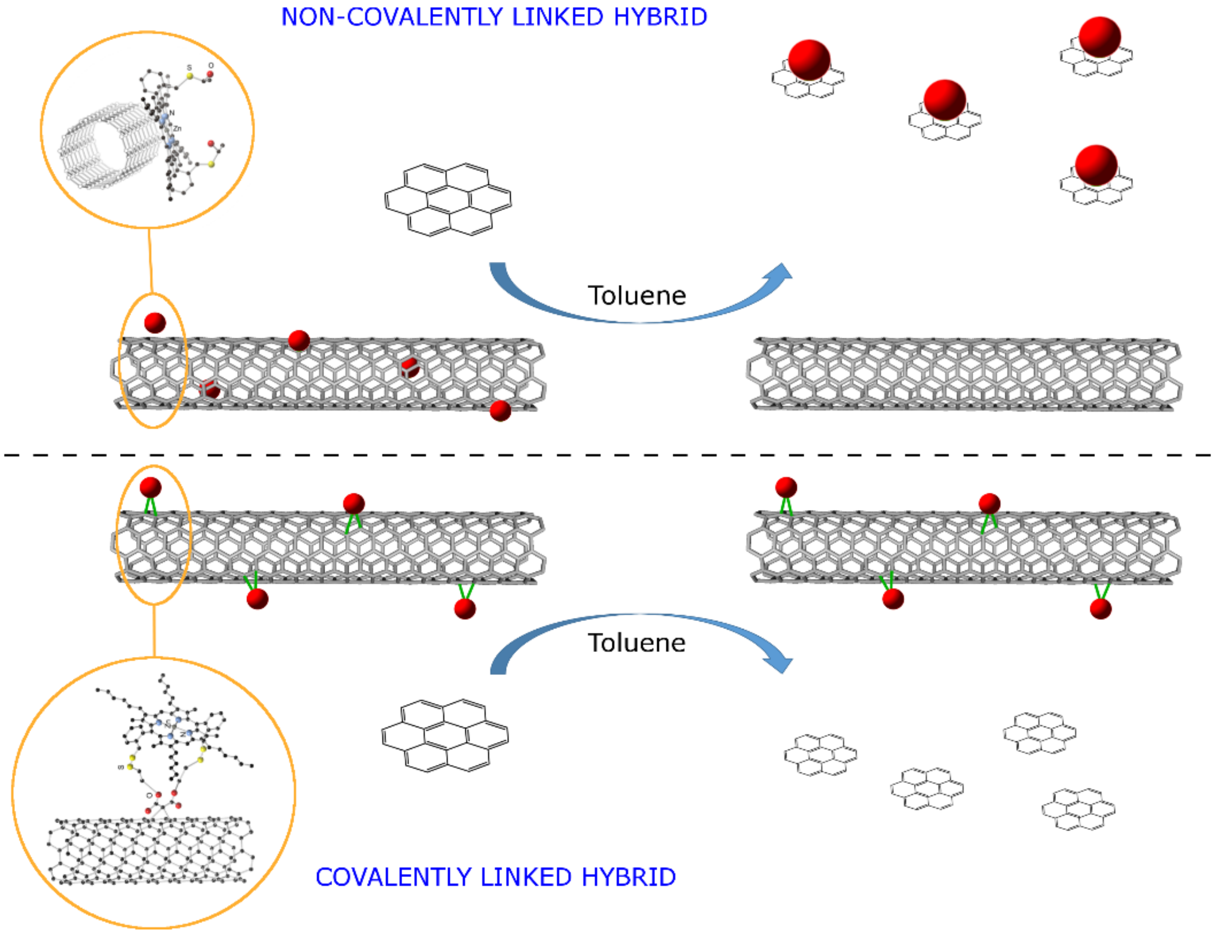

Figure S49. Addition of coronene (1μM solution in toluene) and washing process of the 1:1 toluene : Chloroform suspentions non-covalent and covalent linked Zn(II)-porphyrin@SWNTs (**2**) and (**6**).

As discussed in the main text, coronene was introduced as a competitive candidate likely to disrupt the interactions between the SWNTs and Zn(II)-porphyrin systems This experiment sheds light on the nature of Zn-porphyrin-SWNTs and clarify its energy transfer process. Due to the strong delocalized electron density, coronene was expected to display a stronger interaction with porphyrin molecule compared with SWNTs. Coronene was added into the non-covalent and covalent linked Zn(II)-porphyrin@SWNTs suspensions and then the mixtures were washed with toluene several times. The final products were, then,collected by filtration (**Scheme 1**). The solids were redispersed in a 1:1 ethanol and chloroform. Both the filtrates and solids were kept for further analysis, as discussed below. In particularly, **Figure 50a** shows the

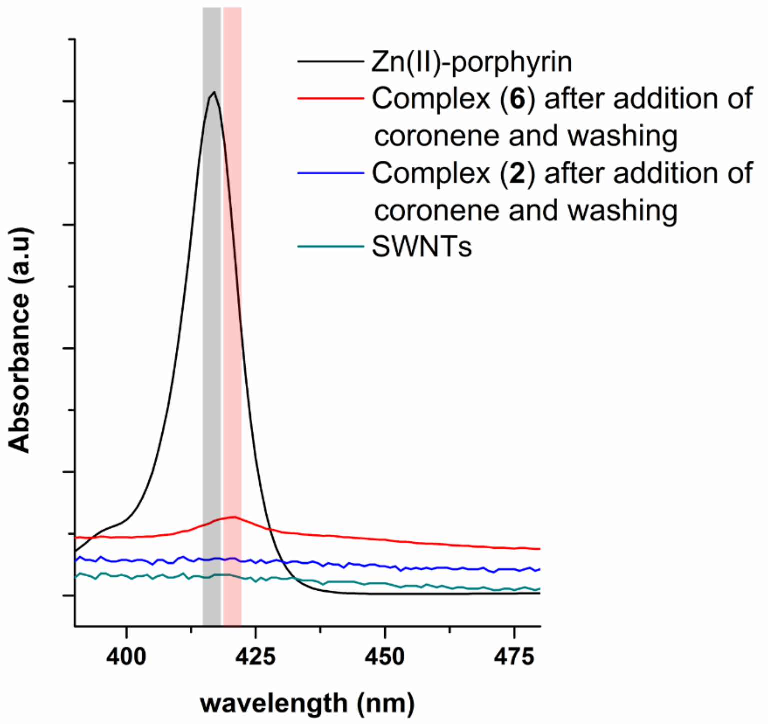

(a)

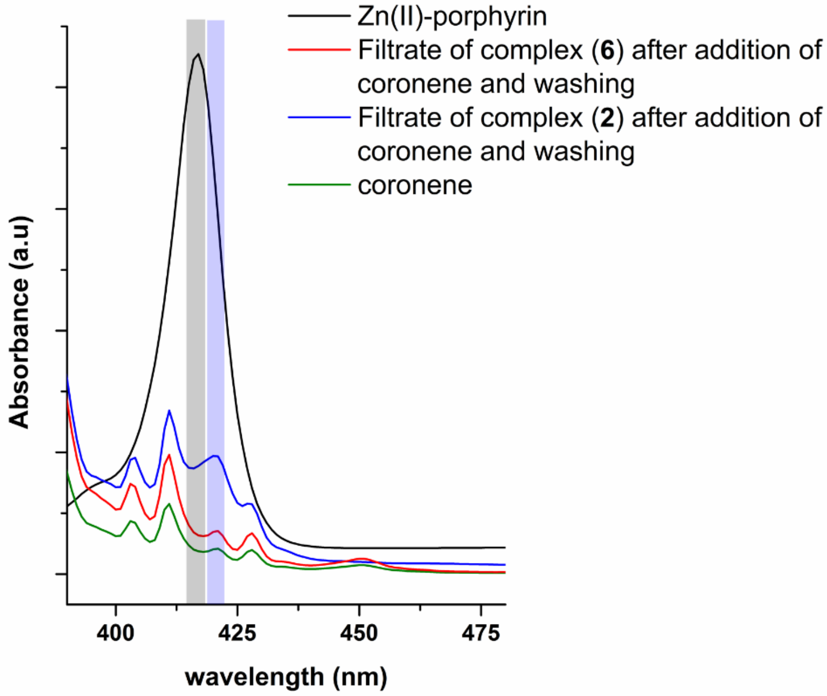

(b)

Figure 50. Competition experiment: **a)** UV-vis spectra of Zn(II)-porphyrin, pristine SWNTs, non-covalent Zn(II)-porphyrin@SWNTs nanohybrids (**2**) and coronene, covalent Zn(II)-porphyrin@SWNTs nanohybrids (**6**) and coronene. After competitive exchanges with coronene, all the products were collected by filtration and dispersed in a 1:1 chloroform

mixture (1 μM); **b)** UV-vis spectra of the filtrate of non-covalent Zn(II)-porphyrin@SWNTs (**2**) and covalent Zn(II)-porphyrin@SWNTs nanohybrids (**6**) after competitive exchanges with coronene

UV-vis spectra of the Zn(II)-porphyrin, non-covalent and covalent linked Zn(II)-porphyrin@SWNTs and SWNTs after treatment with coronene and toluene. The characteristic absorption band at 436 nm of the non-covalent Zn(II)-porphyrin@SWNTs (**2**) complex disappeared and the final products exhibit a similar UV-vis absorption spectrum as the intact SWNTs suspension. This suggests that after treating the system with coronene and washing with toluene, the Zn(II)-porphyrin was successfully displaced from the SWNTs surface. In contrast, the characteristic absorption profile of the covalent Zn(II)-porphyrin@SWNTs complex (**6**) can be seen even after coronene treatment. **Figure 50b** shows the UV-vis measurement of Zn(II)-porphyrin, covalent and non-covalent linked Zn(II)-porphyrin@SWNTs filtrates after treatment with coronene/toluene and as well as free coronene. UV-vis absorption spectrum of the non-covalent linked Zn(II)-porphyrin@SWNTs filtrate shows that the absorption band at 420 nm was enhanced and the absorption band at 450 nm disappears followed by the appearance of broader peak also emerged at 546 nm. The latter corresponds to the secondary absorption band of intact Zn(II)-porphyrin. These results demonstrate that coronene and Zn(II)-porphyrins were linked together to form a new non-covalent complex. Zn(II)-porphyrin can, therefore, be displaced from the SWNTs walls only when coronene is added. Such experiments point out the difference of the chemical nature of the covalent and non-covalent interactions between porphyrin and SWNTs.

18. SWNTs-Porphyrin UV-vis titration

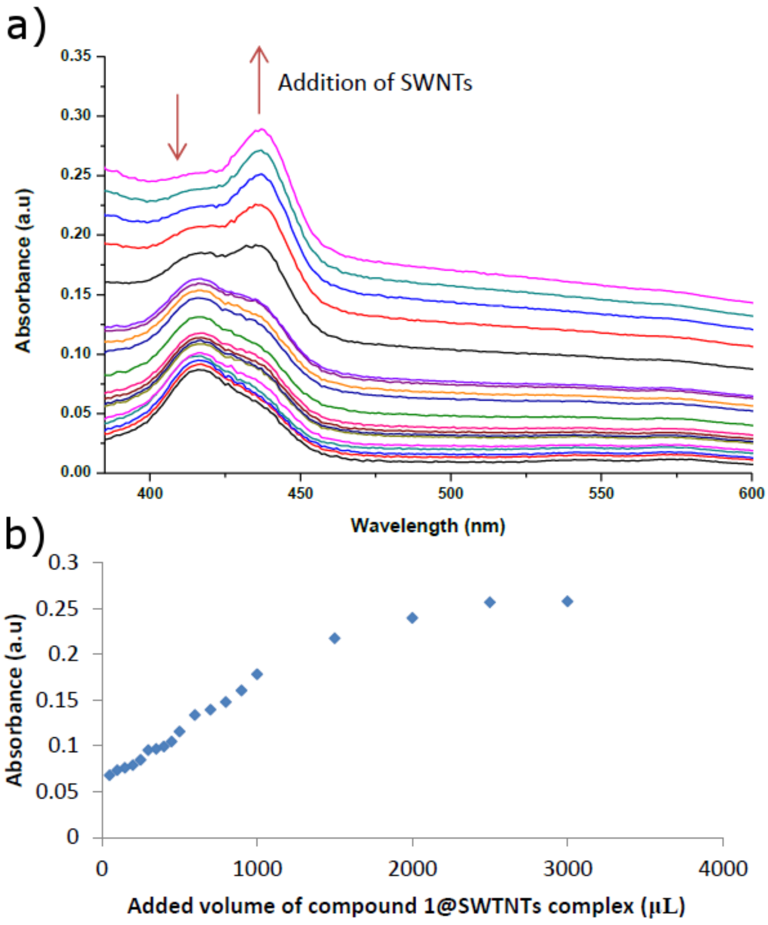

Figure S51. a) UV-visible absorption enhance behaviour of non-covalently linked Zn(II)-porphyrin@SWNT (**2**) nanohybrids; **b)** UV-visible absorbance intensity with respect to added volume (430 nm). Zn(II)-porphyrin is dispersed in a 1:1 mixture of o-DCB and chloroform (3 mL, 1 μM) and titrated with a dispersed mixture of Zn(II)-porphyrin (**1**) (0.5 μM) and SWNTs in a 1:1 o-DCB and chloroform suspension

As shown in **Figure S51a** and **S52b**, the addition of SWNTs into Zn(II)-porphyrin solution results in an enhancement of absorption intensity, while a separate absorption band emerges at 430 nm. The appearance of a new band suggests that by adding SWNTs into Zn(II)-porphyrin (**1**) solution, an electron donor-acceptor interaction system, involving porphyrin (**1**) and SWNTs is formed and driven by π-π stacking interactions. During the assembling process, the Soret band of unreacted Zn(II)-porphyrin (**1**) disappeared as a result of the formation of a supramolecular non-covalent Zn(II)-porphyrin@SWNTs complex (**2**). The charge transfer band at 430 nm observed upon UV-Vis titration also corresponds to the maximum UV-Vis absorption of the non-covalently linked complex (**2**) shown in **Figure 11** of the manuscript. The maximum UV-Vis absorption for the complex (**6**) was observed at 420 nm due to a red-shift of the Soret band and no charge transfer bands were observed.

References

[1] P. He and M. Bayachou, *Langmuir* **2005**, *21*, 6086-6092.
[2] B. Zhao, H. Hu, A. Yu, D. Perea and R. C. Haddon, *J. Am. Chem. Soc.* **2005**, *127*, 8197-8203.
[3] Y. Qin, L. Liu, J. Shi, W. Wu, J. Zhang, Z.-X. Guo, Y. Li and D. Zhu, *Chem. Mater.* **2003**, *15*, 3256-3260.
[4] Y. Lin, D. E. Hill, J. Bentley, L. F. Allard and Y.-P. Sun, *J. Phys. Chem. B.* **2003**, *107*, 10453-10457.
[5] T. Hasobe, S. Fukuzumi and P. V. Kamat, *J. Am. Chem. Soc.* **2005**, *127*, 11884-11885.
[6] P. Thordarson, *Chemical Society Reviews* **2011**, *40*, 1305-1323.
[7] P. Clausen Mathias, S. Galiani, l. Serna Jorge Bernardino de, M. Fritzsche, J. Chojnacki, K. Gehmlich, B. C. Lagerholm and C. Eggeling in *Pathways to optical STED microscopy, Vol. 1* **2014**.
